# Supplementary material for: AMORE-Isoprene v1.0: a new reduced mechanism for gas-phase isoprene oxidation
Source: Geosci Model Dev. Author manuscript; Available in PMC 2025 Jan 27. (PMC11770595; doi:10.5194/gmd-16-1801-2023)
Supplement: Supplement1 [file NIHMS2038935-supplement-Supplement1.pdf]

Supplement of Geosci. Model Dev., 16, 1801–1821, 2023
https://doi.org/10.5194/gmd-16-1801-2023-supplement
© Author(s) 2023. CC BY 4.0 License.

Geoscientific Model Development
Open Access

Supplement of

AMORE-Isoprene v1.0: a new reduced mechanism for gas-phase isoprene oxidation

Forwood Wiser et al.

Correspondence to: V. Faye McNeill (vfm2103@columbia.edu)

The copyright of individual parts of the supplement might differ from the article licence.

Supporting Information for AMORE-Isoprene v1.0: A new reduced mechanism for gas-phase isoprene oxidation

Code availability. CMAQv5.3.3 is available at https://github.com/USEPA/CMAQ and archived at doi: 10.5281/zenodo.5213949. The exact CMAQ code used in this work and CMAQ output is available at doi: 10.23719/1527975.

Code and data for the AMORE algorithm is available at https://github.com/fcw2110/AMORE_supplementary_files and archived at doi: 10.5281/zenodo.7106505.

S1. Full Isoprene Mechanism Update

The Caltech full Isoprene mechanism (Bates and Wennberg 2018) was updated for this work. The original mechanism did not contain complete oxidation pathways for all of the species present in the mechanism. The reasoning behind this was that several species did not have published or known oxidation schemes. Since this mechanism was not designed for use in 3D models, the incomplete chemistry was not an issue.

However, for this work, complete chemistry was needed, as we were attempting to utilize the accuracy of the full mechanism for the reduction process. To do so, we needed to update the full mechanism to contain oxidation pathways for all species. Most of the work for this process was already done by Bates et al. in the preparation of their reduced plus isoprene scheme. However, they completed oxidation pathways only after lumping many multifunctional isoprene species together. They used SAR and existing mechanisms (MCM) to complete the chemistry for these species.

We utilized the information from their reduced plus isoprene scheme to create a complete full mechanism. To do so, we first created a correspondence between lumped species in the reduced plus mechanism and species in the full mechanism without an oxidation pathway. From there, the reactions of the lumped species were replicated for the un-lumped species. If a lumped species was a reactant, then a new reaction was created for each species that it represented. If a lumped species was a product, then the reaction coefficient was divided evenly into the represented species set, thus conserving carbon flux between the two mechanisms.

In addition to incomplete isoprene chemistry, the Caltech full mechanism did not complete oxidation pathways for species considered outside of the isoprene scheme. The oxidation chemistry for these additional species was taken from the MCM scheme.

The updated Caltech full isoprene mechanism is ideal for box model simulations for the purpose of mechanism reduction, as it can be considered more accurate for dynamic oxidant concentrations and common oxidation products such as formaldehyde and carbon monoxide.

All of the changes made to the mechanism at this stage were additive. No reactions were removed from the mechanism. Compared to the original mechanism, the degradation of highly oxidized isoprene derived species increases the production of carbon monoxide, formaldehyde, glyoxal, and methylglyoxal. The most significant change is in carbon monoxide production over long run times. These changes also reduce the overall concentration of oxidized isoprene species, such as isoprene nitrates, over long time frames. The plots below show the impact of the changes for a single simulation. Effects are similar for other simulation conditions.

Figure S1

Simulation settings: 200 ppb H_2O_2, sza/photo constant = 1, NO_2 = 0.2 ppb, ISOP = 10 ppb

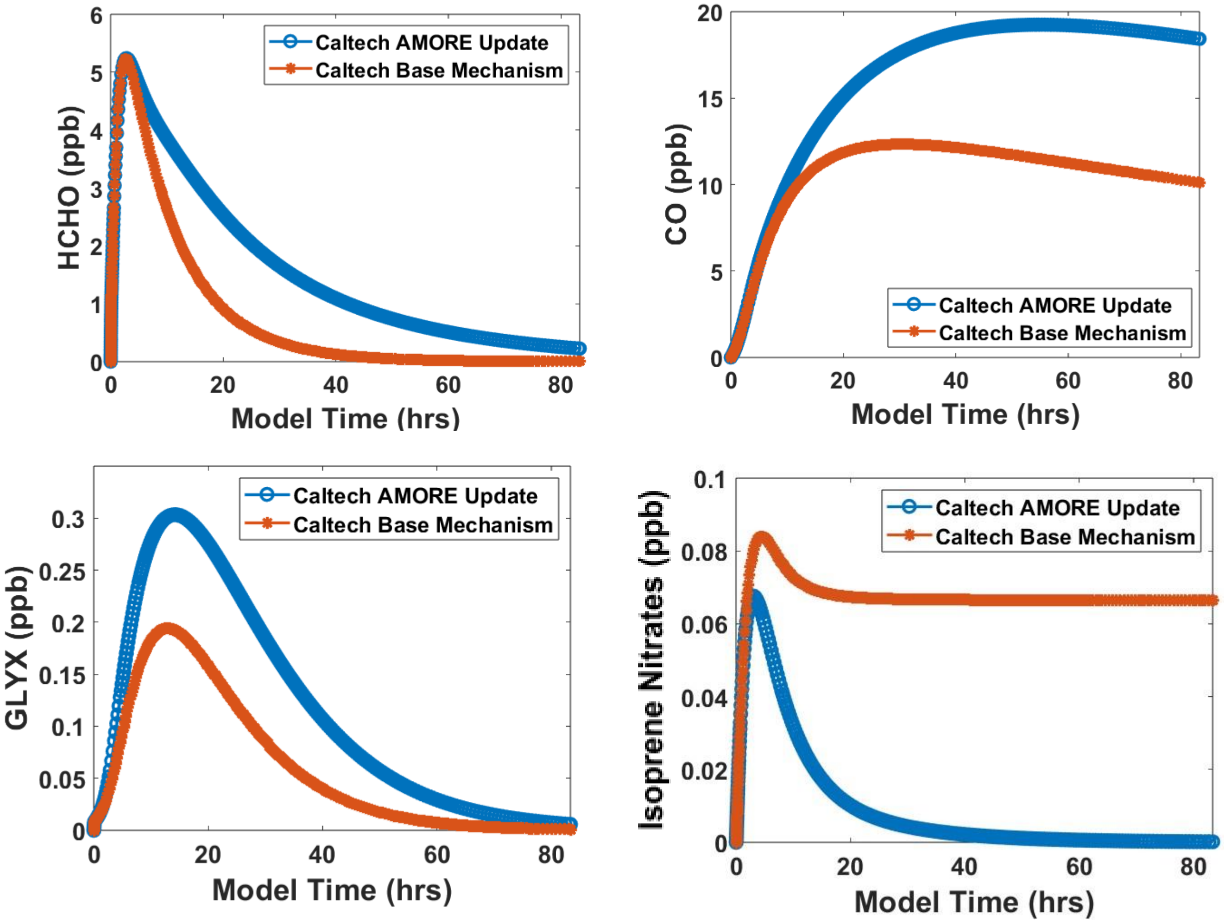

The changes to the degradation of small molecules had a significant effect on nitrogen radical cycling. In particular, the addition of degradation pathways for the $CH3CO_3$ radical had a major effect on NO_2 cycling and ozone production. The impact of this particular addition is shown in the plots below, wherein the base caltech mechanism, updated caltech mechanism, and updated mechanism without $CH3CO_3$ degradation are shown.

Figure S2 Simulation settings: 200 ppb H_2O_2, sza/photo constant = 1, NO_2 = 5 ppb, ISOP = 10 ppb

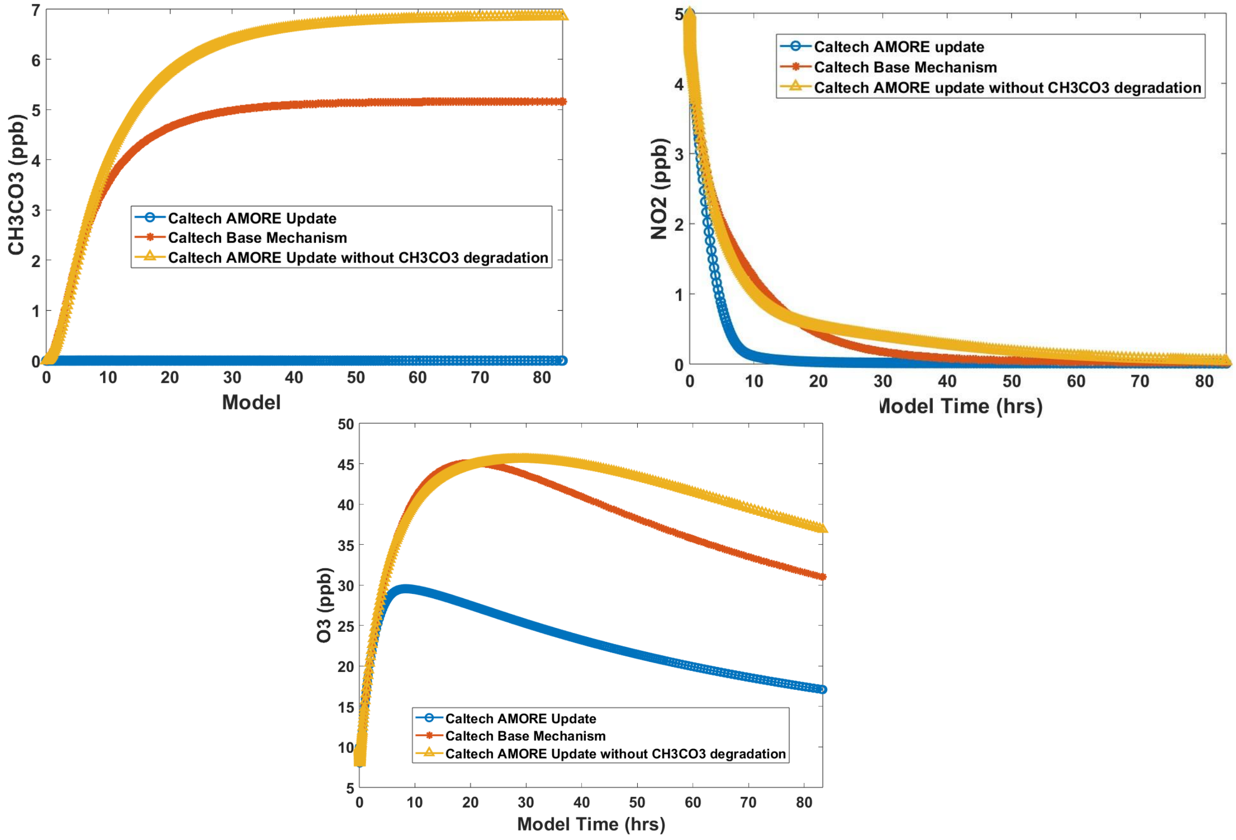

As shown in the graphs, the degradation of $CH3CO_3$ leads to a significant reduction in NO_2 concentrations, and therefore a reduction in ozone concentrations.

The Caltech full isoprene mechanism was used as the basis for the Caltech Reduced mini mechanism, which has become one of the default mechanisms for the GEOSChem 3D model and has undergone validation by its authors (Bates and Jacob, 2019). The Caltech reduced mini mechanism is still 108 species and therefore a more highly reduced version could be useful for large-scale modeling. It is important to note that the extensions of the oxidation chemistry we made to the baseline Caltech mechanism are analogous to the extensions made by the Caltech group prior to reduction when they created the Caltech reduced plus and Caltech reduced mini mechanisms.

Most experimental datasets, including the Paulot data discussed in section 3.2 and shown in in Figure 8 (9 in revision) as well as data found in the EUROCHAMP (Muñoz and Gómez-Alvarez (2008)) and FIXCIT (Nguyen et al. (2014)) databases, do not effectively enable an intercomparison between the Caltech full mechanism and our extended Caltech base mechanism. This is because no changes were made to the first three generations of isoprene chemistry or the IEPOX chemistry, and the species these experimental studies tracked were

mostly confined to that part of the mechanism. Therefore, agreement with, e.g., the Paulot et al. IEPOX chamber data shown in Figure 8 (9 in revision) is the same for the Caltech Full and our extended mechanism. The main impacts of the extended oxidation chemistry are on later-generation oxidation products, carbon monoxide, formaldehyde, ozone, and nitrogen radicals, that is, species which, for the most part, were not tracked in these experiments.

We used data from two EUROCHAMP experiments to compare the update to the Caltech baseline. The experiment with the most data for isoprene and stable operating conditions suitable for simulation was the high O_3 experiment (Muñoz (2021b)). This experiment, along with the high NO experiment (Muñoz (2021a)), were the only ones identified that had simultaneous measurements of NO_x, isoprene, ozone, formaldehyde, and carbon monoxide. For the high NO experiment, the NO_2 concentrations reported in the data had multiple peaks and troughs, suggesting unreported loss and addition throughout the experiment, making it unsuitable for simulation. However, with some assumptions, reasonable agreement was observed between the two mechanisms and the reported MVK, isoprene, and formaldehyde data. The Caltech and the extended Caltech mechanisms performed similarly.

For the high O_3 experiment, agreement between the two mechanisms and measured ozone and isoprene was strong. Both mechanisms were biased high for formaldehyde compared to the experimental data, with similar accuracy.

Figure S3
Simulation Conditions:
P = 1000 mbar
T = 295 K
ISOP = 210 ppb
O_3 = 210 ppb
H_2O_2 = 10 ppb
CO = 200000 ppb
NO_x = 0 ppb
Photolysis = 0

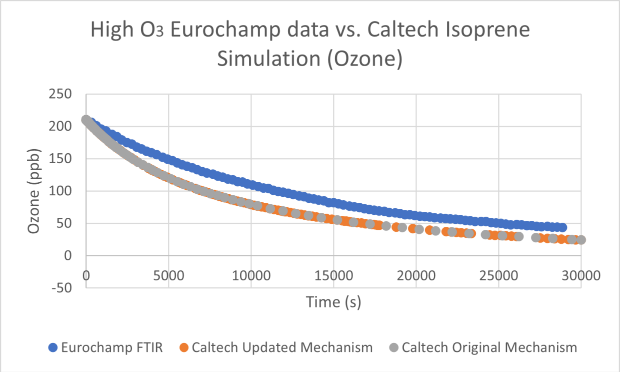

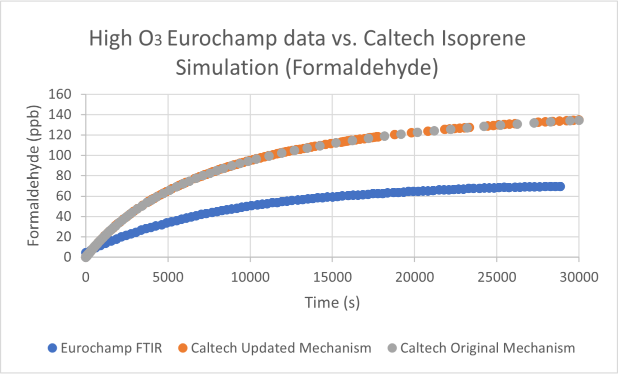

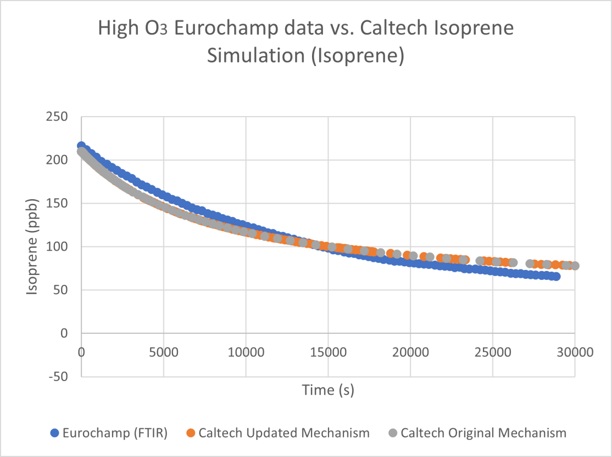

In the supplementary files, there is a folder entitled “Caltech Full Mechanism (AMORE update)” which contains all of the files related to this mechanism. The “READ_ME” file gives a brief description of each file in the folder.

S2. Priority Species List

Below is a list of priority species which were included in the final mechanism and tested for accuracy under multiple conditions.

Table S1

Name	Name in Mechanism
Isoprene	ISO
Isoprene epoxydiols (lumped)	IEPOX
Isoprene nitrates (lumped)	ISON
Formaldehyde	HCHO
Methyl vinyl ketone	MVK
Methyl glyoxal	MGLY
Glyoxal	GLY
Methacrolein	MACR
Peroxyacetyl nitrate	PAN
Peroxyacetyl radical	ACO3
Methyl radical	MO2
Hydroxyl radical	OH
Hydroperoxyl radical	HO2
Nitric oxide	NO
Nitrogen dioxide	NO2
Nitrate radical	NO3
Ozone	O3

S3. Directed Relation Graph method discussion

The Directed Relation Graph (DRG) method has been employed in the past to reduce chemical mechanisms. This method was initially trailed for AMORE and it was determined that the method was unsuitable for use in this project. The reasoning behind this is discussed below.

The DRG method starts by representing the full mechanism as a graph. This aspect of the method was retained in the AMORE algorithm. In the DRG method, all species are represented as nodes within the mechanism graph. Edges are created between species in which the first species contributes directly to the production of the second species. The edge is directed from the first species to the second species.

Each edge is assigned a weighting based on the fraction of production of the end species that is contributed by the start species. This weighting quantifies the relationship between the two species and serves as a natural metric of which edges are more important.

The DRG reduction method works by removing edges from lowest weight onward until a desired mechanism size is reached. Initially, the removal of edges leads to the severing of ties between two species, usually by removing the reactions or components of reactions that they share. However, once a species has been isolated from all other species, it is effectively removed as well. Thus, edge removal leads to the removal of both reactions and species. There are several methods that elaborate on this process, by complexifying the edge weighting scheme. Hothever, the primary issue with DRG for the application to the isoprene mechanism is not the weighting scheme, but rather the method of removal.

In the DRG method, model reduction only occurs through the removal of species and reactions. Thus, this method assumes that there is a sufficient number of unimportant species and reactions that can be identified and removed without loss of accuracy. The implication for the isoprene mechanism, where our target mechanism is one to two orders of magnitude smaller than our original mechanism, is that upwards of 90% of the species and reactions can be removed without significant impact to the species we are trying to retain. Clearly, this assumption is not valid for this application. Additionally, given that important species are divided amongst multiple longer paths, the minimum achievable mechanism size that still maintained a connection between isoprene and the species listed in SI section 2 using the DRG method was still larger than the desired mechanism size of <10 intermediate species.

Given this issue, there was no apparent way to implement the DRG method in creating the AMORE-Isoprene mechanism. The AMORE algorithm differs from the DRG method by attempting to summarize the core elements of the entire mechanism first by creating a much smaller representative structural graph, and then filling in the coefficients by probing the full mechanism. This is a categorically different approach because it does not rely on removing unimportant parts of the mechanism.

S4.1 Cycle to node reduction algorithm

One of the key components of the AMORE-Isoprene algorithm is the Yield Estimation Algorithm (YEA) (section 2.3.1). The YEA is used to estimate the time independent yields of any isoprene derived species assuming all reactions have gone to completion and all oxidant concentrations are held constant. The primary source of difficulty and inaccuracy in this method is the treatment of cycles. Cycles are instances in which directed edges form a loop between species, meaning that carbon can return to its starting point within the cycle.

In a non-cyclical section of the graph, carbon moves in one direction, and can be tracked simply by measuring the branching ratio of the set of products from a given reaction. Within cycles, carbon may move within the cycle multiple times and the distribution of carbon to products connected to the cycle is not obvious. However, the distribution of carbon is uniquely determined by the input conditions (under constant oxidant concentrations) and thus should be able to be incorporated into a model.

The goal of the cycle sub algorithm is to accurately estimate the yield of species that are sinks for a cycle from a source species for the cycle. Source species are defined as those with directed edges that enter the cyclical section. Sink species are defined as those with directed edges that go from the cycle to the sink species. Source and sink species are outside of the cycle, and thus their carbon is not cycled.

The first step in the cycle sub algorithm is to treat the entire cycle as a single entity that behaves like a normal species, with carbon passing through to other species. Thus, the complicated dynamics that occur within the cycle must be estimated. The equation below shows the y_a of species A that is in or connected to the cycle.

$$\mathrm{k}_{r,net} = k_r \times [reactant] \text{ if reactant} = \text{input oxidant}, k_r \text{ else}$$
$$\mathrm{f}_{a,b} = \sum_{r=1}^{R} k_{r,net} \times c_{b,r}/c_{a,r} \text{ if a} = \text{reactant, b} = \text{product}$$
$$\mathrm{y}_a = \sum_{n=1}^{N} f_{n,a} \text{ if } \mathrm{f}_{a,n} = 0, (f_{n,a}/f_{a,n})\alpha \text{ if } \mathrm{f}_{a,n} > 0$$

R is the number of reactions, r is an individual reaction, k_r is the rate constant for reaction r, $k_{r,net}$ is the effective rate constant under constant oxidant concentration, N is the number of species, n is an individual species, $f_{a,b}$ is the production of b from a, $c_{a,r}$ is the stoichiometric coefficient of a in reaction r, and y_a is the yield of a species within a cycle. Here, α is a tunable parameter which modulates how much is contained within the cycle versus distributed outward. When this parameter is large, flux tends to spread evenly through the cycle before being distributed outward, leading to a more even distribution outward. When this parameter is small, flux is quickly distributed outside of the cycle, leading to an uneven distribution within. This parameter was tuned to most accurately replicate the yields from the F0AM simulation, and a value of α = 0.0000001 was used, signifying a tendency to quickly distribute flux outward.

S4.2 YEA Performance

The performance of the YEA was measured in comparison to the simulated yields from the F0AM 0-D box model. The YEA was used in place of the box model to reduce computation time. Thus, comparison to the box model was used to determine whether the reduction time was worth the accompanied loss in accuracy.

Times were measured on a dell Inspiron 15 with a Ryzen 5000 series processor. The YEA algorithm was able to calculate yields for 10 species under 128 different conditions (1280 yield values) in 4.27 seconds. Running the equivalent F0AM simulations took 132 seconds for the ODE solver, 387 seconds to calculate the yields from the concentration data, and 909 seconds to calculate the yield of isoprene nitrates which is a large grouping of species. This leads to a total run time of 1428 seconds to calculate the equivalent yields in F0AM. The F0AM yield calculation was not optimized for time performance, so they may have significant room for improvement. The YEA was between 30 and 300 times faster than the equivalent F0AM simulation.

The accuracy of the YEA was measured by direct comparison to the F0AM yields. Between all species and conditions, the YEA was off by a factor of 2 on average, with a maximum discrepancy of a factor of 11. Given that yields can span many orders of magnitude, and the AMORE algorithm functions based on changes in yields, this is strong performance for the use case and resulting decrease in time cost. For each measured species, a linear regression analysis was run comparing the F0AM yields to the YEA. The slopes of all regressions were positive as expected, and the R^2 values ranged from 0.01 (MVK) to 0.7 (IEPOXd). Species with higher R^2 values tended to have higher variation in yields. A csv file containing the yields of F0AM, YEA, and the input conditions is given in the supporting files (YEA_analysis_data).

S5. Recombination of paths in the algorithm process:

The figure below outlines the process of the pathway combination algorithm. After the important pathways are identified, they are grouped based on shared oxidants, and then paths are created within these groupings which minimize the number of necessary intermediate steps.

Figure S4

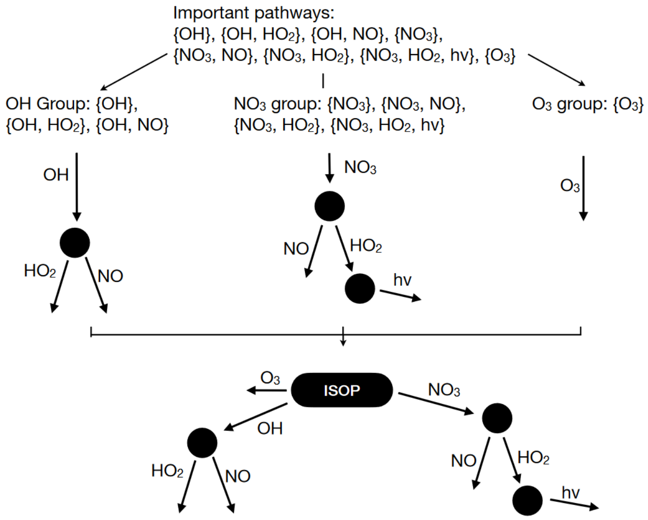

S6. Algorithmically generated mechanism

Below is the algorithmically generated mechanism, with species names assigned manually for clarity. Mechanism structure was determined using the pathway importance algorithm and reaction stoichiometric coefficients were determined using the yield estimation algorithm. Rate laws were not algorithmically determined.

Table S2

Number	Reaction
SR1	ISOP + OH --> ISOPOO
SR2	ISOPOO --> 0.022 IEPOX + 0.040 ISOPN + 0.277 MVK + 0.037 GLY + 0.224 MACR + 0.867 MGLY + 1.12 HCHO
SR3	ISOPOO + NO --> 0.049 IEPOX + 0.344 ISOPN + 0.405 MVK + 0.027 GLY + 0.321 MACR + 0.196 MGLY + 1.468 HCHO
SR4	ISOPOO + HO2 --> 0.072 IEPOX + 0.041 ISOPN + 0.362 MVK + 0.091 GLY + 0.281 MACR + 0.711 MGLY + 1.286 HCHO
SR5	ISOP + NO3 --> INO2
SR6	INO2 --> 2.3 ISOPN + 0.166 MVK + 0.334 GLY + 0.022 MACR + 0.288 MGLY + 0.44 HCHO
SR7	INO2 + HO2 --> IPN

SR8	IPN --> 0.014 IEPOX + 1.94 ISOPN + 0.163 MVK + 0.582 GLY + 0.023 MACR + 0.323 MGLY + 0.415 HCHO
SR9	IPN + hv --> 1.6 ISOPN + 0.166 MVK + 0.264 GLY + 0.026 MACR + 0.115 MGLY + 1.22 HCHO
SR10	INO2+ NO --> 2.127 ISOPN + 0.199 MVK + 0.064 GLYX + 0.029 MACR + 0.084 MGLY + 0.788 HCHO
SR11	ISOP + O3 --> 0.046 IEPOX + 0.135 ISOPN + 0.094 MVK + 0.023 GLY + 0.192 MACR + 0.2 MGLY + 0.974 HCHO

S7. Discussion of isoprene hydroxy nitrates (IHN)

The first version of the optimized AMORE mechanism was run in CMAQ for initial evaluation. The results were generally favorable, however it was noted that the species IHN had very high concentrations, as shown in the figure below, which compares IHN concentrations in AMORE-Isoprene to ISON concentrations, a comparable species in CRACMM base. The concentrations of IHN were much higher than the ISON concentrations, and upon further investigation, it was found that IHN in AMORE-Isoprene was also much higher than in the Caltech full mechanism. Although IHN was not initially considered an important species, newer research has suggested that it is worth retaining IHN accuracy for NO_x cycling (Vazquez et al 2020).

Figure S5

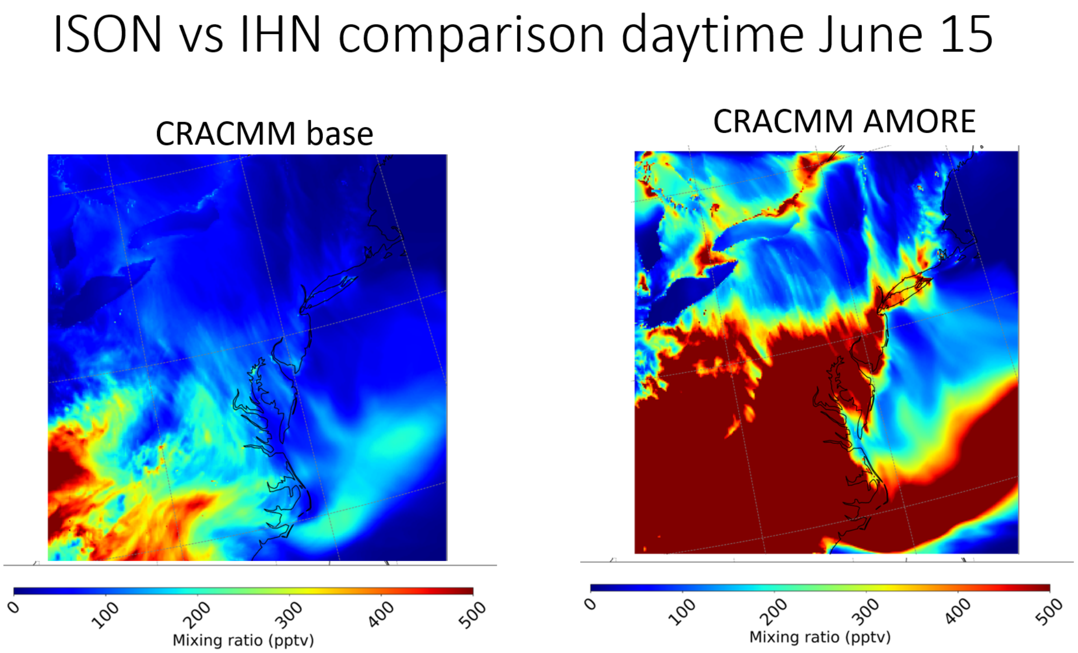

S8. Table of rate law descriptions

The table below describes the rate laws for each reaction in Table 4 and how they were determined.

Table S3

#	Reaction	Rate Law	Source
R1	ISO + O3 = 0.07 MACR + 0.189 MVK + 0.58HCHO + 0.25 HO + 0.25 HO2 + 0.58 HCHO + 0.08 MO2 + 0.1 ACO3 + 0.09 H2O2 + 0.1 MACP + 0.461 MACR + 0.14 CO + 0.28 ORA1 + 0.15 OLT	1.58E-14 exp(-2000/T)	Caltech, sum of isoprene + O_3 rates
R2	ISO + NO3 = INO2 + 0.3 HCHO + 0.3 NO2 + 0.3 ISON	2.95E-12 exp(-450/T)	Caltech, sum of isoprene + NO_3 rates
R3	ISO + HO = ISOP + 0.02 MO2	2.69E-11 exp(390/T)	Caltech, sum of isoprene + OH rates
R4	ISOP + HO2 = ISHP + 0.6 HO2 + 0.15 HCHO	4.5E-13 exp(1300/T)	Standard form of many HO_2 reactions in Caltech full, A constant calibrated for best match
R5	ISOP + NO = 0.14 IHN + 0.7 HCHO + 0.44 MVK + 0.88 HO2 + 0.78 NO2 + 0.28 MACR + 0.021 GLY	2.7E-12 exp(350/T)	Standard form of many NO reactions in Caltech full, A constant calibrated for best match
R6	ISHP + HO = ISOP	4.6E-12 exp(200/T)	Modeled after reaction from Caltech full: ISOP1OH2OOH + OH = ISOP1OH2OO : 4.6E-12*EXP(200./TEMP);
R7	INO2 + HO2 = IPN + HO	3.14E-14 exp(580/T)	Standard form of many HO_2 reactions in Caltech full, A constant calibrated

			for best match
R8	INO2 + NO = 0.2 ISON + 0.9 HCHO + 0.5 MGLY+ 0.8 MVK+ 0.5 NO2 + HO2+ 0.1 MO2	9.42E-16 exp(580/T)	Calibrated for INO_2 pathway balancing
R9	IPN + HO2 = 0.2 ISON + 0.8 NO2 + 0.4 HCHO + 0.05 GLY + 0.1 MGLY + 0.4 MACR + HO2 + 0.94 MVK + 0.1 MO2	3.4E-11 exp(390/T)	Calibrated for HCHO production rate
R10	IHN + HO = ISON + HO + 0.2 IEPOX	2.4E-7 exp(580/T)	Calibrated for IHN concentration accuracy
R11	ISHP + HO = 0.05 IPC + 0.15 HCHO + 0.05 MGLY + 0.15 MACR + 0.02 GLY + 0.2 MVK + 0.4 NO2 + 0.58 IEPOX + 0.8 HO	2.97E-11 exp(390/T)	Standard form of many OH reactions in Caltech full, A constant calibrated for best match
R12	ISHP = 0.4 HCHO + 0.1 MGLY + 0.06 ACO3	Photol(HCHO_RAD_RACM2)	Chosen from RACM2 photolysis options to best match rate in box model
R13	IPC + NO = 0.35 NO2 + 0.8 NO	1e-10	Calibrated for NO_x cycling
R14	ISON + HO = CO + 0.12 NO2	5e-11	Calibrated for ISOPN accuracy
R15	ISON + NO3 = CO	2e-14	Calibrated for ISOPN accuracy
R16	IHN = HNO3	2.3e-5	Published IHN degradation rate
R17	IEPOX + HO = HO	5E-11 exp(-400/T)	Match IEPOX degradation rates
R18	ISOP + MO2 = HO2 + 1.31 HCHO + 0.159 MACR + 0.250 MVK + 0.250 MOH + 0.250 ROH + 0.023 ALD + 0.018 GLY+ 0.016 HKET	3.4E-14exp(221/T)	RACM2

R19	ISOP + ACO3 = 0.5 HO2 + 0.5 MO2 + 1.048 HCHO + 0.219 MACR + 0.305 MVK + 0.5 ORA2	8.4E-14 exp(221/T)	RACM2
R20	ISOP + APIP2 = 0.96 HOM + 0.48 ROH + 0.48 HCHO + 0.48 MVK + 0.48 HO + 0.48 HO2 + 0.04 ELHOM	1e-10	RACM2
R21	ISOP + APINP2 = 0.96 HOM + 0.48 ROH + 0.48 HCHO + 0.48 MVK + 0.48 NO2 + 0.48 HO2 + 0.04 ELHOM	1e-10	RACM2
R22	ISOP + LIMNP2 = 0.96 HOM + 0.48 ROH + 0.48 HCHO + 0.48 MVK + 0.48 NO2 + 0.48 HO2 + 0.04 ELHOM	1e-10	RACM2

S9. Species naming convention

The species in the AMORE-Isoprene mechanism were named using common naming conventions. All species with direct analogues were named the same as in other mechanisms. This includes isoprene epoxy diols (IEPOX) and isoprene hydroxy nitrates (IHN). Other species were named in a similar manner to the Caltech reduced mechanism, with I serving as the signifier for the isoprene base, and letters H, N, C, P standing for hydroxyl, nitrates, carbonyl, and hydroperoxy groups respectively. The RACM2 naming convention is more abbreviated than that of the Caltech reduced mechanism, and that convention was retained. Thus, names in the F0AM files do not all match those in the CMAQ formatted files. The table below lists each species, the representative chemical formula, and the names given in both CMAQ and F0AM format. Representative chemical structures are included in the supplementary files.

To improve consistency in species naming across mechanisms in CMAQ, a couple structures in this work were renamed in the October 2022 CMAQ v5.4 release (https://github.com/USEPA/CMAQ/tree/5.4 and https://doi.org/10.5281/zenodo.7218076):

1. ISON in this work was renamed NALD in CMAQv5.4 CRACMM1AMORE.
2. IHN in this work was renamed ISON in CMAQv5.4 CRACMM1AMORE.

Table S4

Species	Chemical Formula	CMAQ	F0AM	Smiles String
Isoprene	C_5H_8	ISO	ISOP	C=CC(=C)C

isoprene epoxy diols	$C_5H_{10}O_3$	IEPOX	IEPOX	CC(O)(CO)C1CO1
Isoprene hydroperoxy radical	$C_5H_9O_3$	ISOP	ISOPOO	[O]OC/C=C(\CO)/C
Isoprene hydroperoxide	$C_5H_{10}O_3$	ISHP	ISOPOOH	OOCC=C(C)CO
Isoprene nitrates (lumped)	~$C_5H_8N_2O_6$	NALD	ISOPN	-
Isoprene hydroxy nitrate	$C_5H_9NO_4$	ISON	IHN	C/C(=C/CON(=O)=O)CO
Isoprene hydroperoxy carbonyl	$C_5H_8O_3$	IPC	IPC	C/C(C=O)=C/COO
Isoprene nitrate peroxy radical	$C_5H_8NO_5$	INO2	INO2	C/C(=C\CO[O])CON(=O)=O
Isoprene hydroperoxy nitrate	$C_5H_9NO_5$	IPN	IPN	C/C(=C\COO)CON(=O)=O
Peroxy radicals formed from MACR + OH	MW = 101	MACP	MACP	
Formic Acid	MW = 46	ORA1	ORA1	
Terminal alkenes	MW = 42	OLT	OLT	
C3 and higher alcohols	M2 = 60	ROH	ROH	
methanol	MW = 47	MOH		
C3 and higher aldehydes	MW = 58	ALD		
Hydroxy ketone	MW = 74	HKET		
Acetic acid and higher acids	MW = 60	ORA2		
		HOM		

		ELHOM		
		APINP2		
Peroxy radicals from d-limonene and other cyclic diene-terpenes	MW = 185	LIMNP2		
Peroxy radicals from alpha-pinenes and other cyclic terpenes with one double bond	MW = 185	APIP2		

S10. Error Metric Behavior

Plot shows behavior of single species error metric for different sample concentration profiles compared to a reference profile (solid orange). The error metric ranges from 0 to 1.

Figure S6

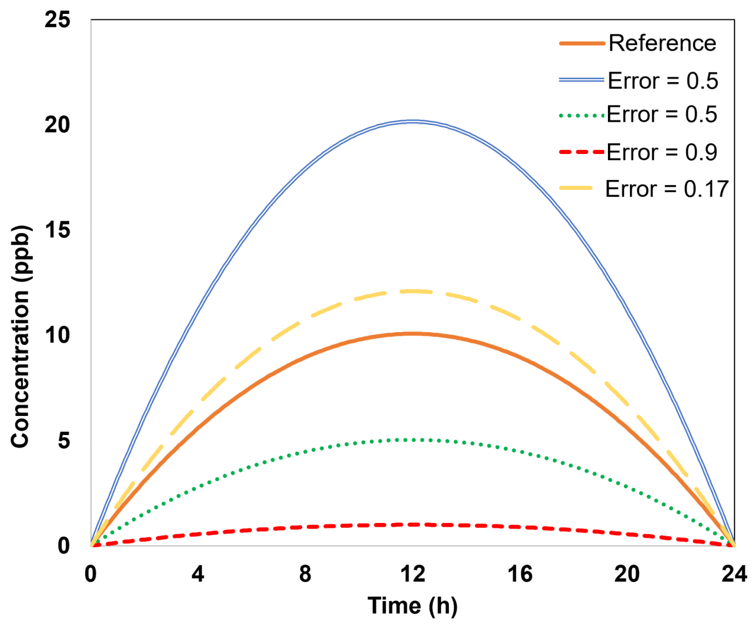

S11. Yield estimation algorithm visualization and discussion

Visual demonstration of yield estimation algorithm for a simple directed acyclic graph. The yield is shown for species A from isoprene. The arrows represent reactions connecting the reactant to the product. The circles represent individual species, and the numbers on the arrows are the fraction of the reactant species diverted to the product. The yield of A is calculated by summing the yields of A from each of its parent species. Thus, the yields of the parent species are calculated first. In this example, the equation for the yield of A (YA) is shown below the graph.

Figure S7

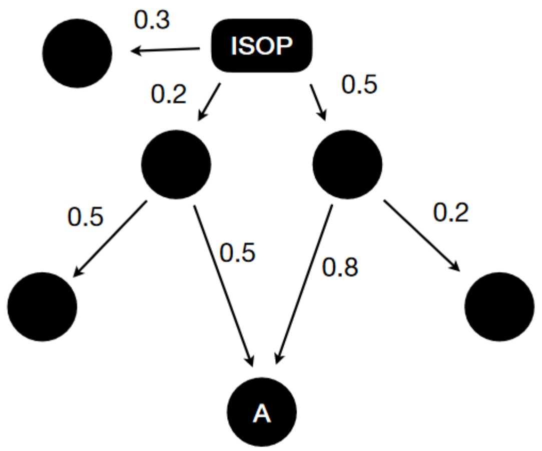

$Y_A = 0.2*0.5 + 0.5*0.8 = 0.5$

More complicated graphs can be calculated using the same method shown above as long as they are directed and acyclic graphs. Cyclic graphs require further processing to be able to estimate the yields, and reactions in which two non-constant reactants are involved are not well represented by a graph structure. In these cases, either a hypergraph is needed, or some loss in information must be accepted.

S12. Effects of Changing inputs on Pathway Importance Algorithm

The mechanism developed in Table S5. The header column describes the changes made to the elevated value inputs to the algorithm. There were seven paths that remained unchanged for all inputs. These include {sol}, {O_3}, {NO_3}, {NO_3, sol}, {OH}, {NO}, and {OH, NO}. All other paths listed were only present in some versions of the inputs. For example, the path {NO, NO_2, NO_3} was only present when NO_2 concentrations were elevated to 5 ppb. When OH concentrations were elevated to 10 x $^{-3}$ ppb (1 ppt, ~2.46 x 10^7 mol/cm^3), the overall number of OH paths decreased. This is likely because OH overshadowed other complementary species, causing their importance to be reduced while the {OH} path increased. All other changes are shown in the table.

Table S5 Green indicates included path, red indicates unincluded path.

Pathway	Default values	NO = 5 ppb, NO_2 = 2 ppb	NO = 5 ppb, NO_2 = 5 ppb	OH = 10^{-3} ppb
{sol}	Green	Green	Green	Green
{O_3}	Green	Green	Green	Green
{NO_3}	Green	Green	Green	Green
{NO_3, sol}	Green	Green	Green	Green
{NO_3, HO_2}	Red	Red	Red	Green
{NO_3, HO_2, sol}	Green	Green	Green	Green
{NO}	Green	Green	Green	Green
{NO, NO_3}	Green	Red	Red	Green
{OH}	Green	Green	Green	Green
{OH, HO_2}	Green	Green	Green	Red
{OH, NO_3}	Green	Green	Green	Red
{OH, NO_3, sol}	Green	Green	Green	Red
{OH, NO}	Green	Green	Green	Green
{OH, NO, NO_3}	Green	Red	Red	Red
{NO, NO_2, NO_3}	Red	Red	Green	Red
{NO, HO_2}	Red	Red	Red	Green

Default values:

OH (ppb) = $[10^{-6}, 10^{-4}]$,
NO (ppb) = $[1.17 \times 10^{-6}, 5.32 \times 10^{-1}]$
NO_2 (ppb) = $[1.01 \times 10^{-4}, 1.01 \times 10^{-2}]$,
NO_3 (ppb) = $[2.3 \times 10^{-4}, 2 \times 10^{-2}]$,
HO_2 (ppb) = $[4.15 \times 10^{-2}, 0.5]$,
O_3 (ppb) = $[16.7, 100]$,

CH3OO (ppb) = [0.1,0.2],
Sol (unitless) = 0.,1]

S13. AMORE Mechanism Box model error tables. Table S6 shows error tables for the AMORE mechanism under six different conditions alongside errors for the other isoprene mechanisms tested. Note that error runs from 0 to 1, with 0 being no error, and 1 being very high error. Errors (as defined in section 2.4.4) were measured for AMORE-Isoprene, Caltech Reduced Plus, Carbon Bond 3, and CRACMM in the F0AM box model under the six conditions described in Table 3. The excel files with this data can be found in the Supporting Information file folder in the AMORE github. The file is named "Multi_mechanism_box_model_error_tables".

Table S6. Error tables for box model simulations

Low NOx	AMORE	CRACMM	Caltech Red Plus	Carbon Bond
Species	Error	Error	Error	Error
IEPOX	0.13	0.42	0.09	0.25
NO	0.25	0.40	0.08	0.32
NO2	0.48	0.62	0.09	0.49
HO	0.28	0.37	0.07	0.32
HO2	0.22	0.54	0.06	0.27
NO3	0.85	0.92	0.18	0.15
ISOP	0.25	0.23	0.05	0.19
O3	0.35	0.35	0.04	0.26
MO2	0.64	0.43	0.15	0.50
ACO3	0.78	0.75	0.22	0.31
PAN	0.89	0.94	0.11	0.57
HCHO	0.28	0.80	0.12	0.23
ISOPN	0.39	0.53	0.11	0.76
GLY	0.72	0.88	0.41	0.43
MGLY	0.71	0.93	0.11	0.34

High NOx	AMORE	CRACMM	Caltech Red Plus	Carbon Bond
Species	Error	Error	Error	Error
IEPOX	0.44	0.36	0.02	0.44
NO	0.10	0.11	0.07	0.10
NO2	0.16	0.12	0.08	0.23
HO	0.22	0.09	0.11	0.31
HO2	0.14	0.16	0.03	0.09
NO3	0.37	0.17	0.05	0.36
ISOP	0.12	0.05	0.05	0.12
O3	0.14	0.03	0.03	0.18
MO2	0.28	0.22	0.14	0.40
ACO3	0.41	0.31	0.15	0.16
PAN	0.53	0.41	0.10	0.28
HCHO	0.05	0.50	0.16	0.12
ISOPN	0.27	0.47	0.04	0.92
GLY	0.47	0.53	0.60	0.88
MGLY	0.49	0.31	0.12	0.50

High NO3	AMORE	CRACMM	Caltech Red Plus	Carbon Bond
Species	Error	Error	Error	Error
IEPOX	0.08	0.88	0.08	0.06
NO	0.06	0.10	0.02	0.46
NO2	0.06	0.10	0.02	0.46
HO	0.10	0.48	0.15	0.30
HO2	0.42	0.77	0.13	0.36
NO3	0.00	0.00	0.00	0.00
ISOP	0.03	0.20	0.07	0.02
O3	0.01	0.01	0.00	0.01
MO2	0.38	0.83	0.22	0.68
ACO3	0.32	0.86	0.30	0.69
PAN	0.20	0.86	0.28	0.51
HCHO	0.13	0.92	0.08	0.41
ISOPN	0.55	0.89	0.55	0.79
GLY	0.60	0.84	0.71	0.67
MGLY	0.56	0.76	0.08	0.15

High NO3, low hv	AMORE	CRACMM	Caltech Red Plus	Carbon Bond
Species	Error	Error	Error	Error
IEPOX	0.22	1.00	0.27	0.37
NO	0.13	0.14	0.05	0.35
NO2	0.13	0.15	0.05	0.37
HO	0.18	0.99	0.52	0.40
HO2	0.46	0.99	0.29	0.41
NO3	0.00	0.00	0.00	0.00
ISOP	0.02	0.10	0.03	0.01
O3	0.03	0.04	0.01	0.01
MO2	0.75	0.97	0.39	0.73
ACO3	0.41	0.99	0.50	0.78
PAN	0.29	0.99	0.50	0.68
HCHO	0.40	0.99	0.04	0.34
ISOPN	0.36	0.82	0.53	0.70
GLY	0.55	1.00	0.94	0.34
MGLY	0.76	0.99	0.21	0.05

High O3	AMORE	CRACMM	Caltech Red Plus	Carbon Bond
Species	Error	Error	Error	Error
IEPOX	0.09	0.51	0.14	0.28
NO	0.08	0.32	0.07	0.26
NO2	0.16	0.63	0.14	0.52
HO	0.25	0.42	0.08	0.28
HO2	0.27	0.62	0.06	0.29
NO3	0.12	0.74	0.10	0.57
ISOP	0.18	0.30	0.08	0.22
O3	0.02	0.05	0.01	0.05
MO2	0.50	0.37	0.13	0.58
ACO3	0.65	0.62	0.22	0.33
PAN	0.57	0.89	0.12	0.46
HCHO	0.24	0.69	0.07	0.28
ISOPN	0.55	0.56	0.18	0.74
GLY	0.77	0.94	0.41	0.60
MGLY	0.61	0.78	0.12	0.12

Chamber Data	AMORE	CRACMM	Caltech Red Plus	Carbon Bond
Species	Error	Error	Error	Error
IEPOX	0.08	0.44	0.14	0.25
NO	0.09	0.23	0.04	0.17
NO2	0.17	0.64	0.09	0.46
HO	0.21	0.29	0.08	0.18
HO2	0.21	0.92	0.09	0.30
NO3	0.82	0.99	0.23	0.40
ISOP	0.22	0.18	0.07	0.10
O3	0.18	0.44	0.02	0.22
MO2	0.64	0.70	0.15	0.45
ACO3	0.77	0.77	0.23	0.34
PAN	0.72	0.98	0.16	0.64
HCHO	0.24	0.84	0.21	0.40
ISOPN	0.46	0.41	0.14	0.71
GLY	0.73	0.97	0.52	0.49
MGLY	0.63	0.96	0.17	0.22

S14. Additional AMORE Box Model Simulations

Only six testing conditions were shown in the box model testing for the AMORE-Isoprene mechanism. Here we include additional box model results at more extreme conditions that are not well represented in the six main testing conditions. From these additional tests, we conclude that the AMORE-Isoprene mechanism is suitable for these additional extreme conditions.

The first additional testing condition was with very low NO_x concentrations. The initial NO_2 concentration was set to 0.05 ppb. The rest of the testing inputs are shown in the error table provided below. Plots of NO_2, OH and ozone are provided as well. At low NO_x, the AMORE-Isoprene mechanism continues to have low error for OH, NO_x, O_3, and other important species. As demonstrated by Figure S9, the AMORE-Isoprene mechanism is the most accurate small mechanism in these conditions.

Table S7 Very low NO_x error table

ISOP = 10ppb, photolysis rate = 1, NO2 = 0.05ppb, H2O2 = 200 ppb				
	AMORE	CRACMM	Caltech Rec	CB6r3
IEPOX	0.13	0.58	0.10	0.22
NO	0.23	0.97	0.09	0.63
NO2	0.20	0.87	0.07	0.60
HO	0.13	0.41	0.05	0.23
HO2	0.18	0.94	0.04	0.25
NO3	0.32	0.99	0.12	0.57
ISOP	0.33	0.29	0.07	0.19
O3	0.14	0.80	0.03	0.57
MO2	0.70	0.69	0.29	0.63
ACO3	0.84	0.76	0.31	0.46
PAN	0.81	0.99	0.25	0.46
HCHO	0.45	0.86	0.24	0.35
ISOPN	0.48	0.67	0.13	0.91
GLY	0.85	0.99	0.40	0.47
MGLY	0.81	0.97	0.16	0.42

Figure S8 Very low NO_x (0.05 ppb) simulation, NO_2, OH, and O_3 Plots

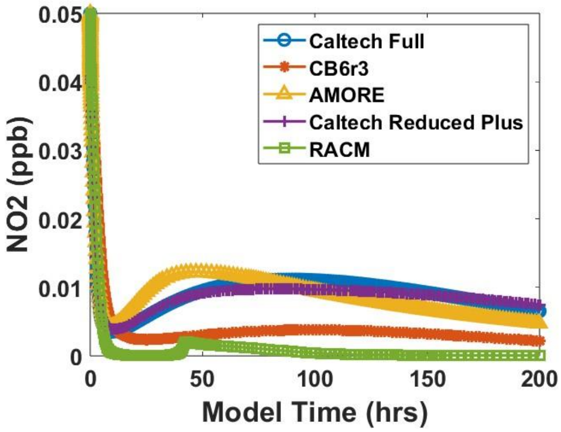

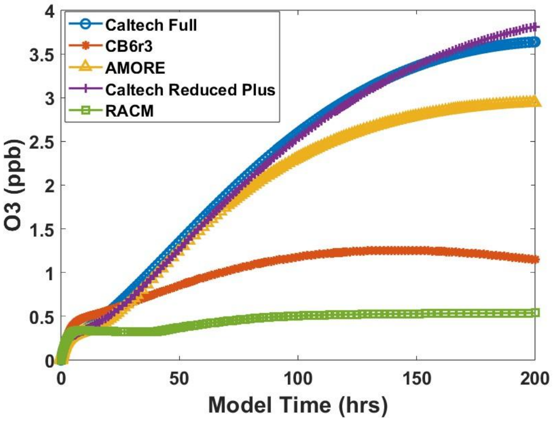

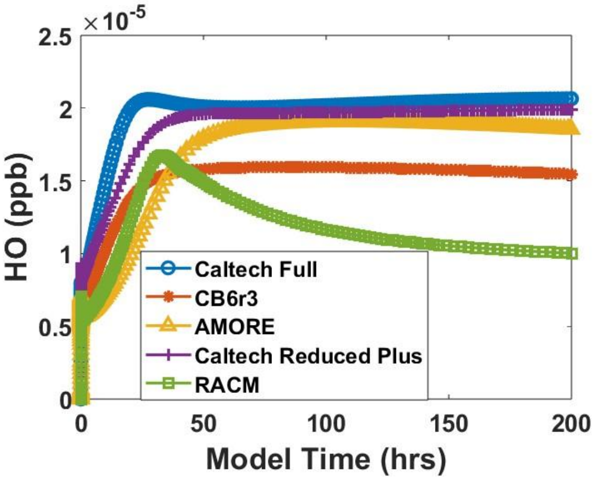

The second additional testing condition was with very high NO_x concentrations. The initial NO_2 concentration was set to 20 ppb. The rest of the testing inputs are shown in the error table provided below. Plots of NO_2 and ozone are provided as well. The results show that AMORE has strong agreement with the Caltech full mechanism for both ozone, NO_2, and many other species (see error table). This suggests that the AMORE isoprene mechanism is suitable for high NO_x conditions.

Table S8. Very high NO_x error table

OH

ISOP = 10ppb, photolysis rate = 1, NO2 = 20ppb, H2O2 = 200 ppb				
	AMORE	CRACMM	Caltech Rec	CB6r3
IEPOX	0.43	0.92	0.03	0.96
NO	0.09	0.10	0.05	0.16
NO2	0.04	0.09	0.04	0.15
HO	0.09	0.08	0.04	0.12
HO2	0.05	0.06	0.02	0.10
NO3	0.05	0.08	0.10	0.20
ISOP	0.04	0.05	0.02	0.08
O3	0.02	0.04	0.03	0.25
MO2	0.20	0.29	0.26	0.50
ACO3	0.17	0.22	0.24	0.25
PAN	0.19	0.25	0.17	0.21
HCHO	0.11	0.39	0.25	0.32
ISOPN	0.33	0.55	0.14	0.98
GLY	0.29	0.29	0.80	0.91
MGLY	0.24	0.28	0.14	0.58

Figure S9 Very high NO_x (20 ppb) simulation, NO_2 and O_3 Plots

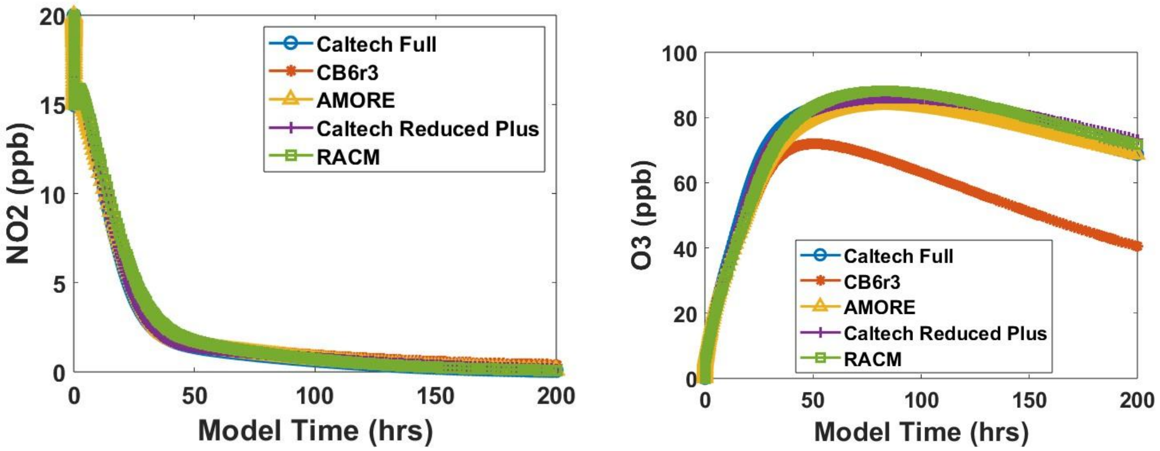

The final additional testing condition was with very high NO_x concentrations and high ozone concentrations. The initial NO_2 concentration was set to 20 ppb and the initial ozone concentration was set to 100 ppb. The rest of the testing inputs are shown in the error table provided below. Plots of NO_2, OH, and ozone are provided as well. At high ozone and NO_x, the AMORE mechanism shows strong agreement for both ozone, NO_2, and OH concentrations (Figure S10), and low error for most species (Table S9). This suggests that the AMORE mechanism is suitable for high NO_x and high Ozone conditions.

Table S9 Very high NO_x and high ozone error table

ISOP = 10ppb, photolysis rate = 1, NO2 = 20ppb, H2O2 = 200 ppb, O3 = 100 ppb

	AMORE	CRACMM	Caltech Rec	CB6r3
IEPOX	0.25	0.33	0.08	0.40
NO	0.17	0.23	0.04	0.22
NO2	0.11	0.15	0.03	0.19
HO	0.36	0.34	0.14	0.12
HO2	0.37	0.46	0.10	0.26
NO3	0.11	0.15	0.02	0.04
ISOP	0.11	0.23	0.04	0.03
O3	0.05	0.07	0.01	0.05
MO2	0.18	0.64	0.16	0.24
ACO3	0.30	0.70	0.22	0.59
PAN	0.18	0.66	0.22	0.31
HCHO	0.13	0.69	0.11	0.24
ISOPN	0.27	0.81	0.28	0.81
GLY	0.21	0.55	0.33	0.83
MGLY	0.38	0.48	0.07	0.17

Figure S10 Very high NO_x (20 ppb) and high ozone (100 ppb) simulation, NO_2, OH, and O_3 Plots

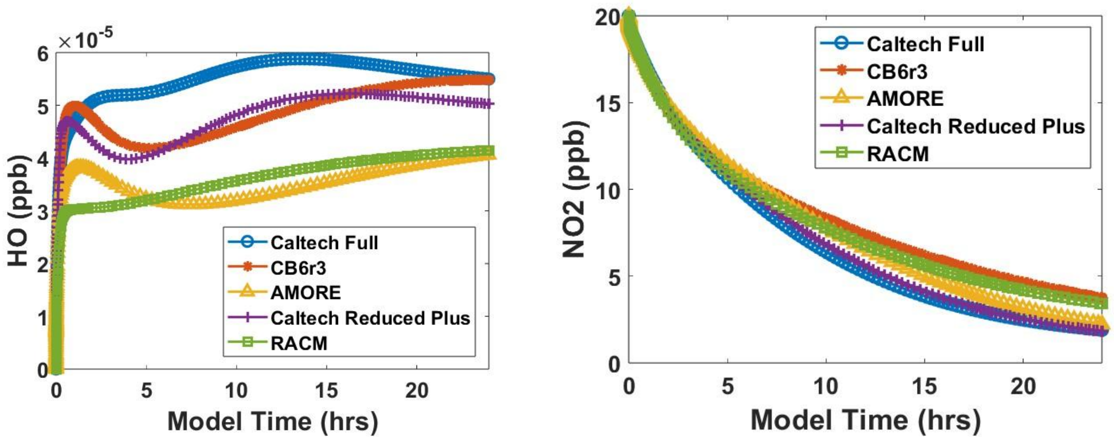

OH

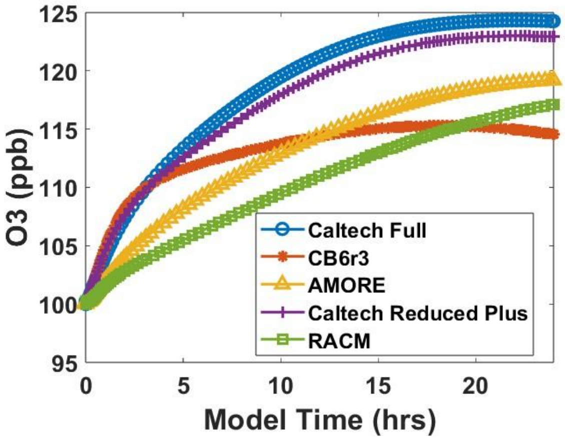

S15. Additional CMAQ Bias Plots

Additional CMAQ results are shown in figure S12. These plots show the binned bias of the AMORE mechanism for different ranges of the measured value. All measurements are from AQS data. The AMORE mechanism (red) is shown in comparison to the CRACMM1 base mechanism (gray) and a prior version of the AMORE mechanism (blue).

The AMORE mechanism shows slightly very slightly increased OC concentrations and slightly decreased isoprene concentrations, however the difference is not significant. There is no discernible difference in the NO_y concentrations between the three mechanisms. Due to issues with the CMAQ run boundary conditions, the overall magnitude of the bias is confounded by many factors unrelated to isoprene. Thus, the main conclusion to be drawn from these graphs is that there is no significant change from the base mechanism.

Figure S11.a) CMAQ isoprene biases

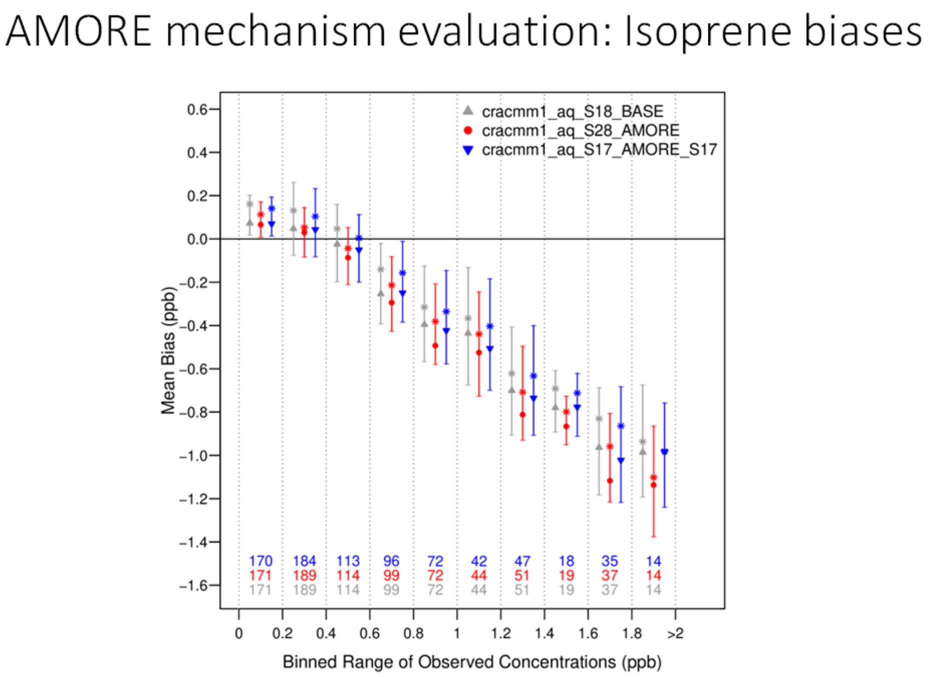

Figure S11.b) CMAQ NOy biases

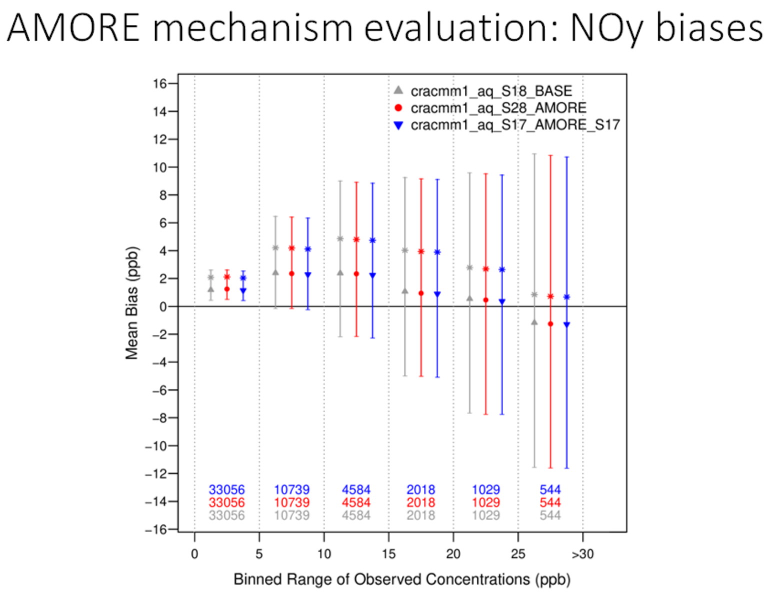

Figure S11.c) CMAQ OC biases

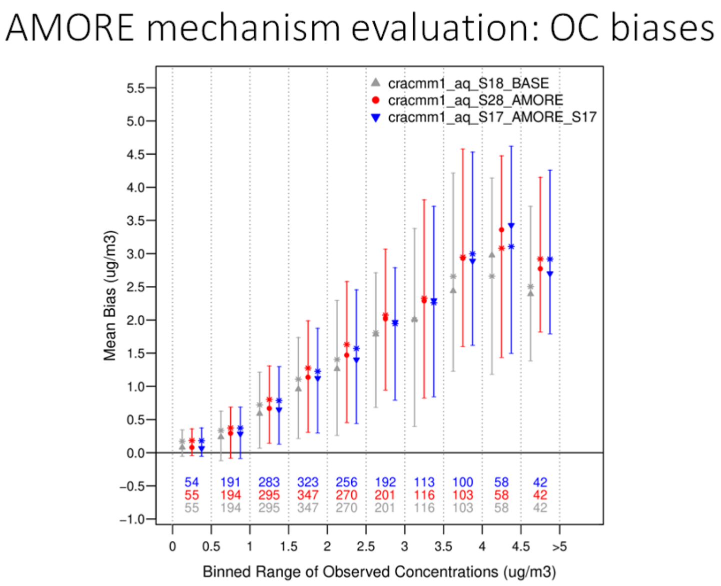

S16. Expanded conditions (lower and higher NO_x) as addendum to Figure 3.

For context, please see Figure 3 in the main paper. We ran the same comparison under lower and higher NO_x conditions. For the ISOPOO + NO reaction shown in Figure 3.a), the improvement is consistent for all NO_x concentrations. Below are plots under the same conditions except for changes to NO initial concentrations:

Figure S12.a) NO = 0.05 ppb

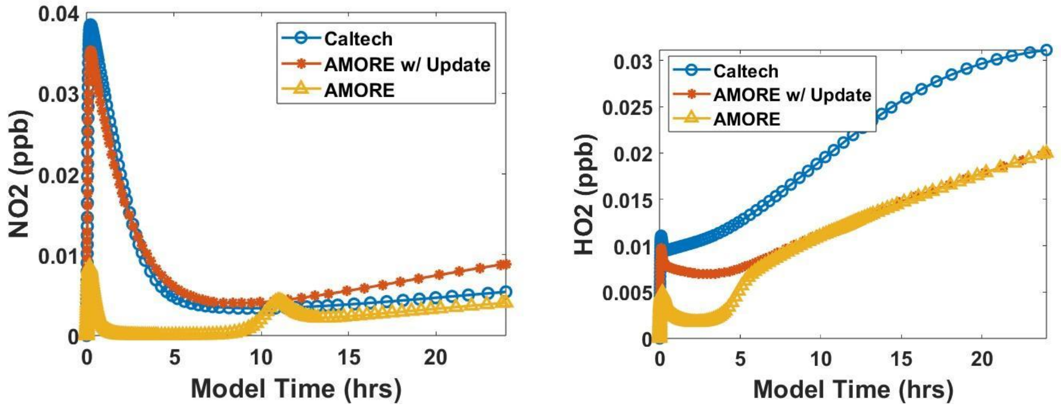

Figure S12.b) NO = 5 ppb

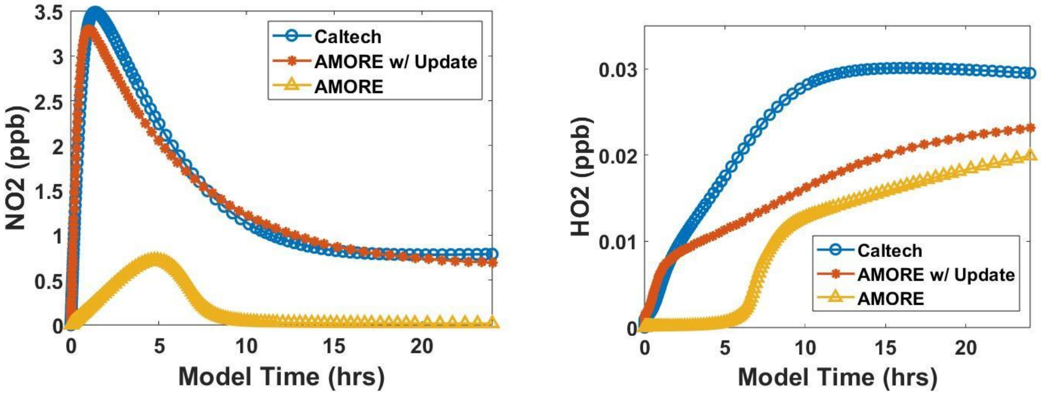

Figure S12.c) NO = 20 ppb

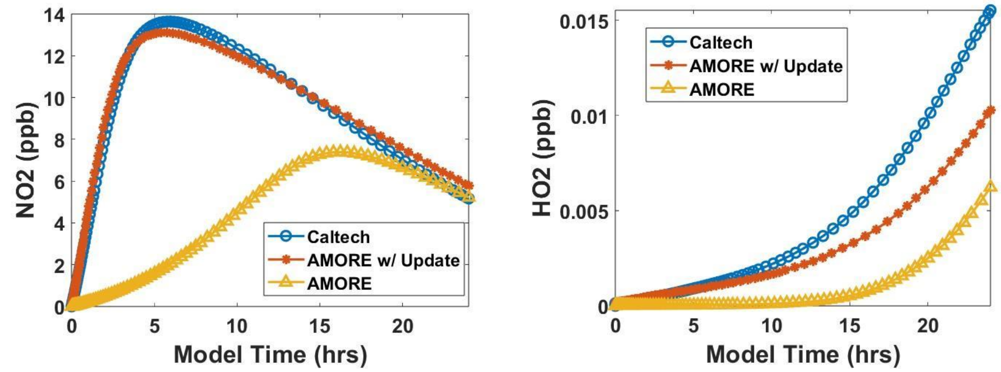

For the ISOPOO + HO2 reaction (figure 3.b), the improvement is only noticeable at lower NO concentrations, where that reaction is more prominent. At higher NO concentrations, the update has no effect. Below are plots under the same conditions except for changes to NO initial concentrations:

Figure S13.a) NO = 0.05 ppb

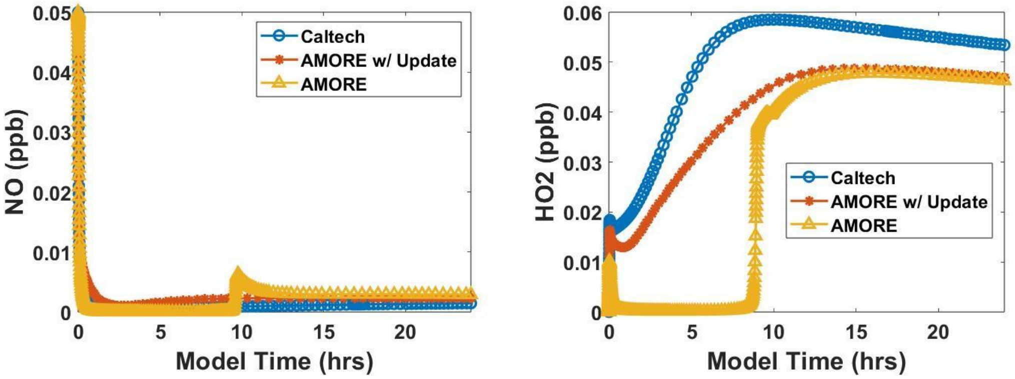

Figure S13.b) NO = 5 ppb

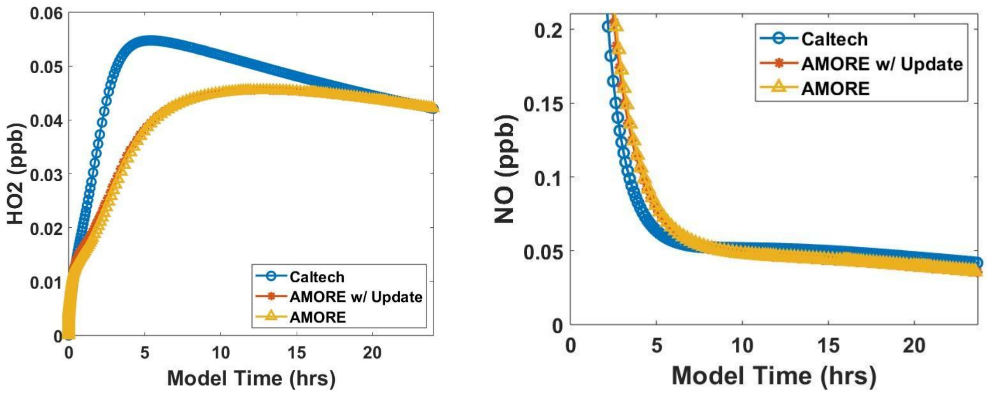

S17. Additional Box Model Plots

The following box model plots show additional species in the six primary conditions tested. The species included are isoprene, ozone, MGLY, GLY, MO2, ACO3 and NO.

Figure S14. Low NO_x

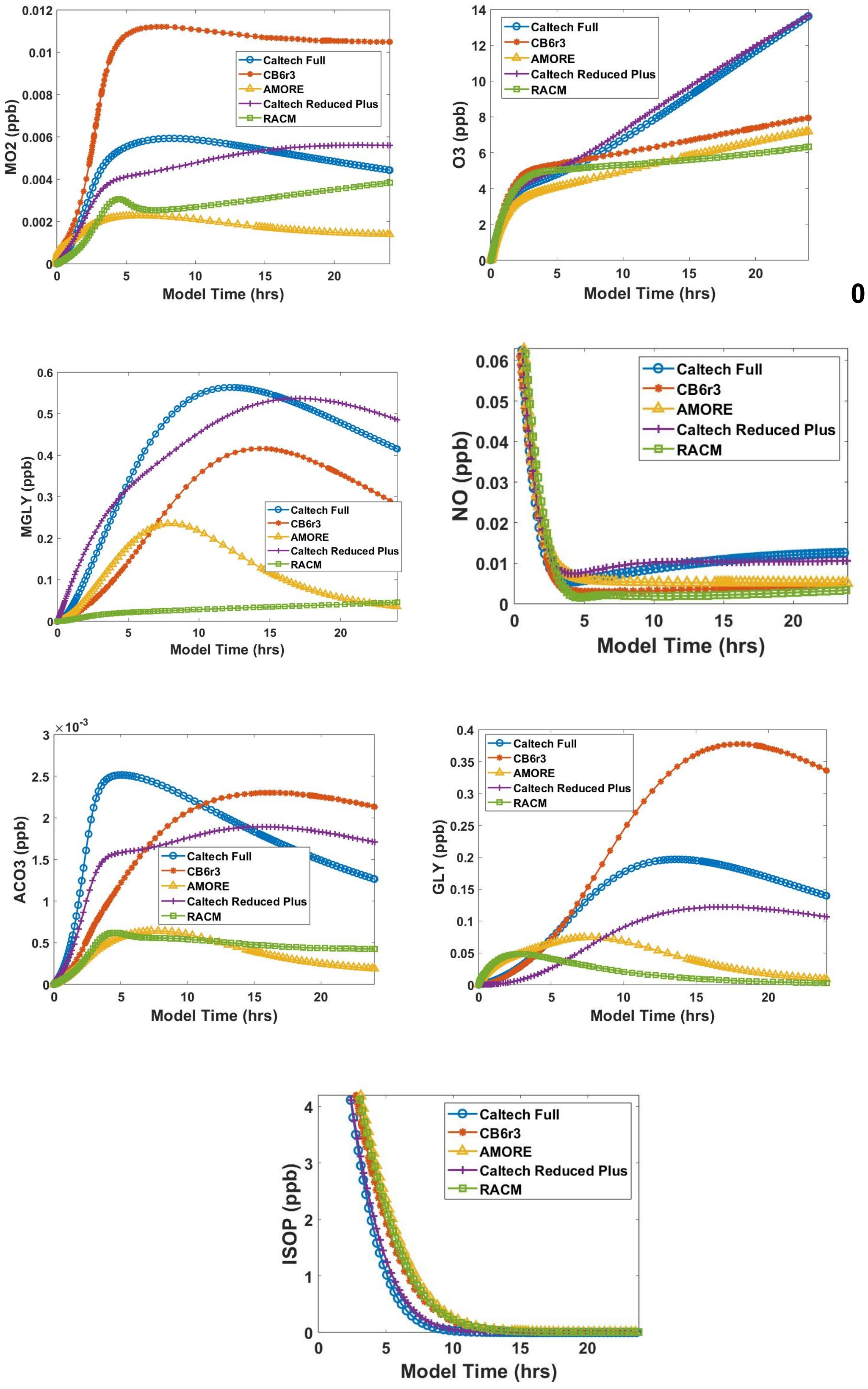
MO2 (ppb)
O3 (ppb)
MGLY (ppb)
NO (ppb)
ACO3 (ppb)
GLY (ppb)
ISOP (ppb)
Model Time (hrs)
Caltech Full
CB6r3
AMORE
Caltech Reduced Plus
RACM
0

Figure S15. High NO_x

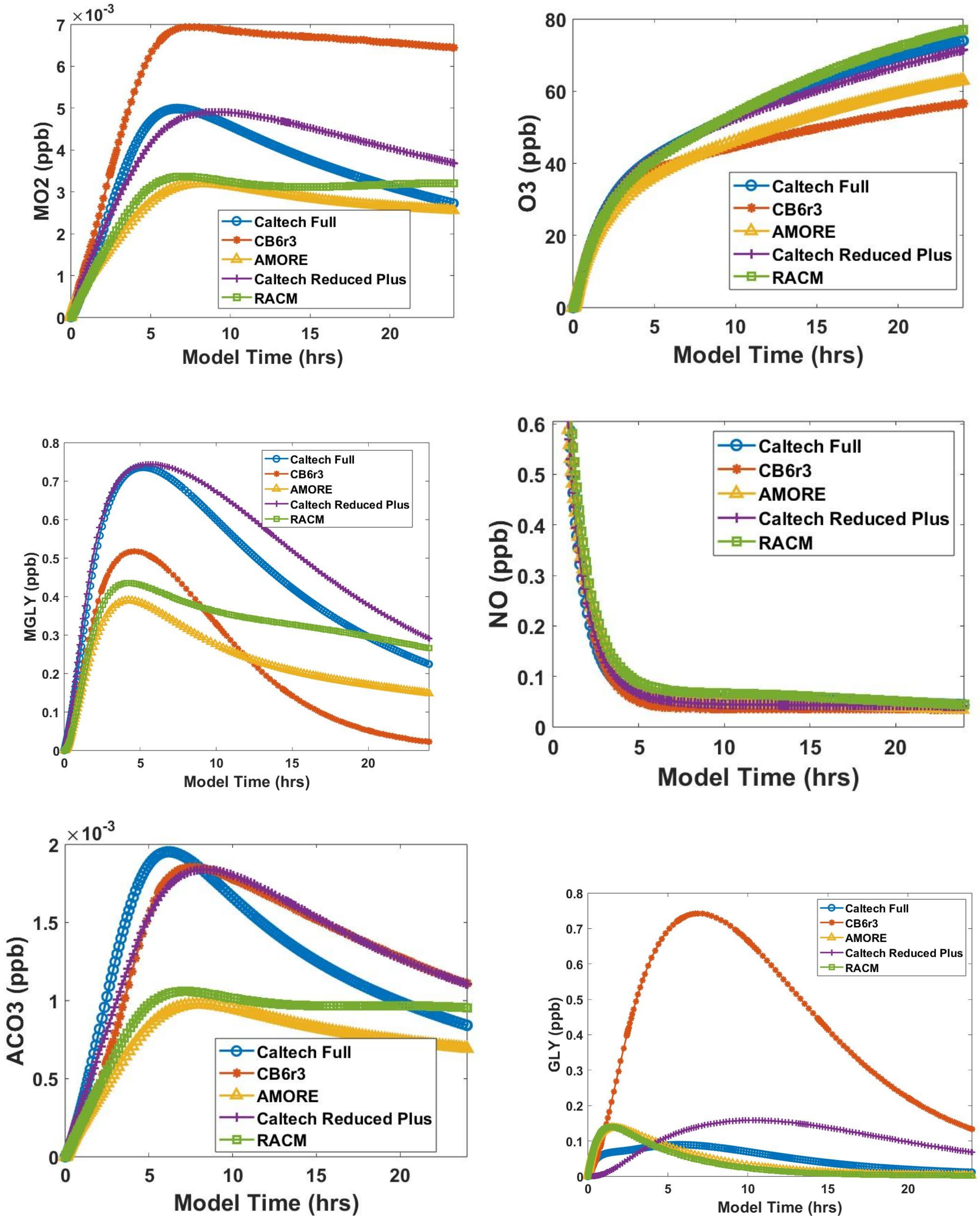

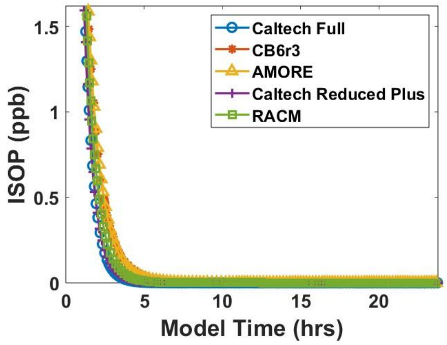

Figure S16. High NO_3

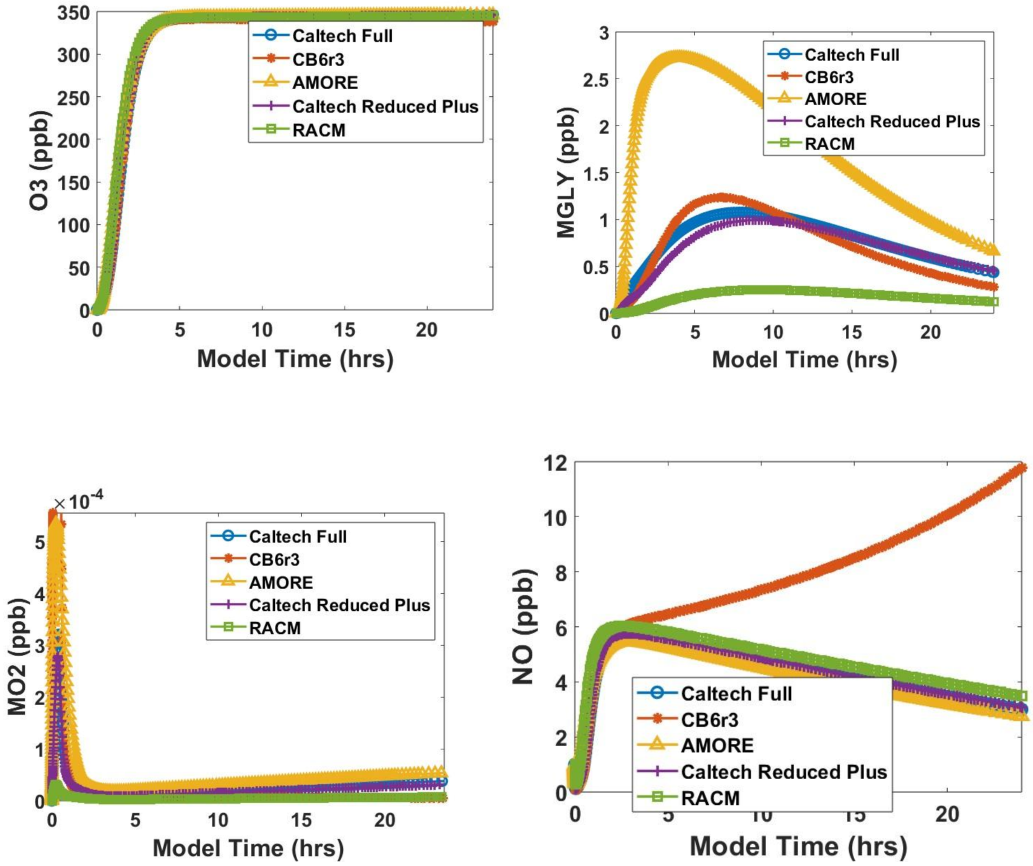

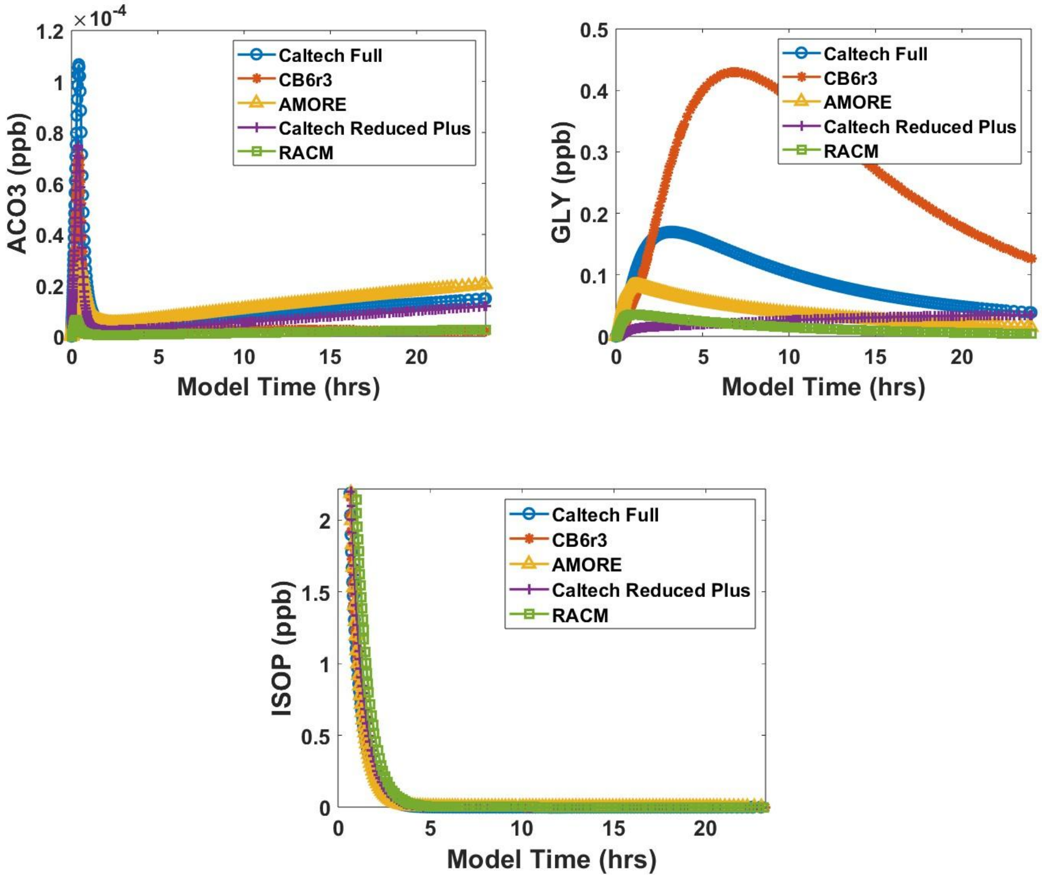

Figure S17. High NO_3 low hv

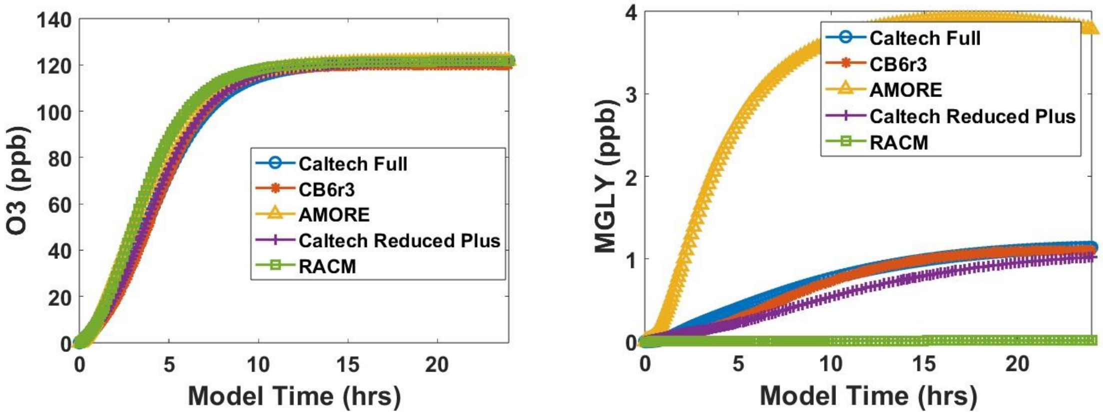

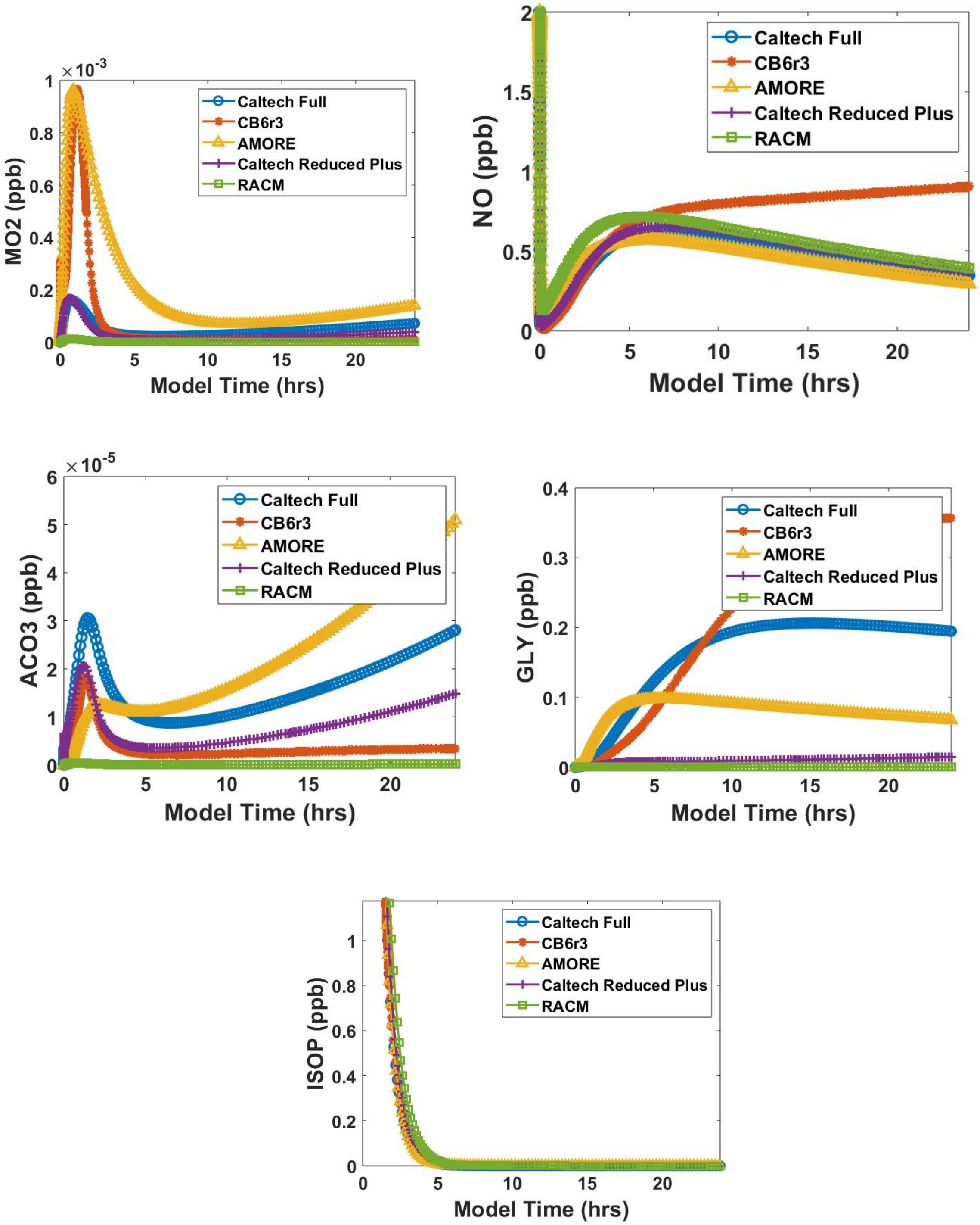

MO2 (ppb)
×10-3
Model Time (hrs)
Caltech Full
CB6r3
AMORE
Caltech Reduced Plus
RACM
NO (ppb)
Model Time (hrs)
Caltech Full
CB6r3
AMORE
Caltech Reduced Plus
RACM
ACO3 (ppb)
×10-5
Model Time (hrs)
Caltech Full
CB6r3
AMORE
Caltech Reduced Plus
RACM
GLY (ppb)
Model Time (hrs)
Caltech Full
CB6r3
AMORE
Caltech Reduced Plus
RACM
ISOP (ppb)
Model Time (hrs)
Caltech Full
CB6r3
AMORE
Caltech Reduced Plus
RACM

Figure S18. High O_3

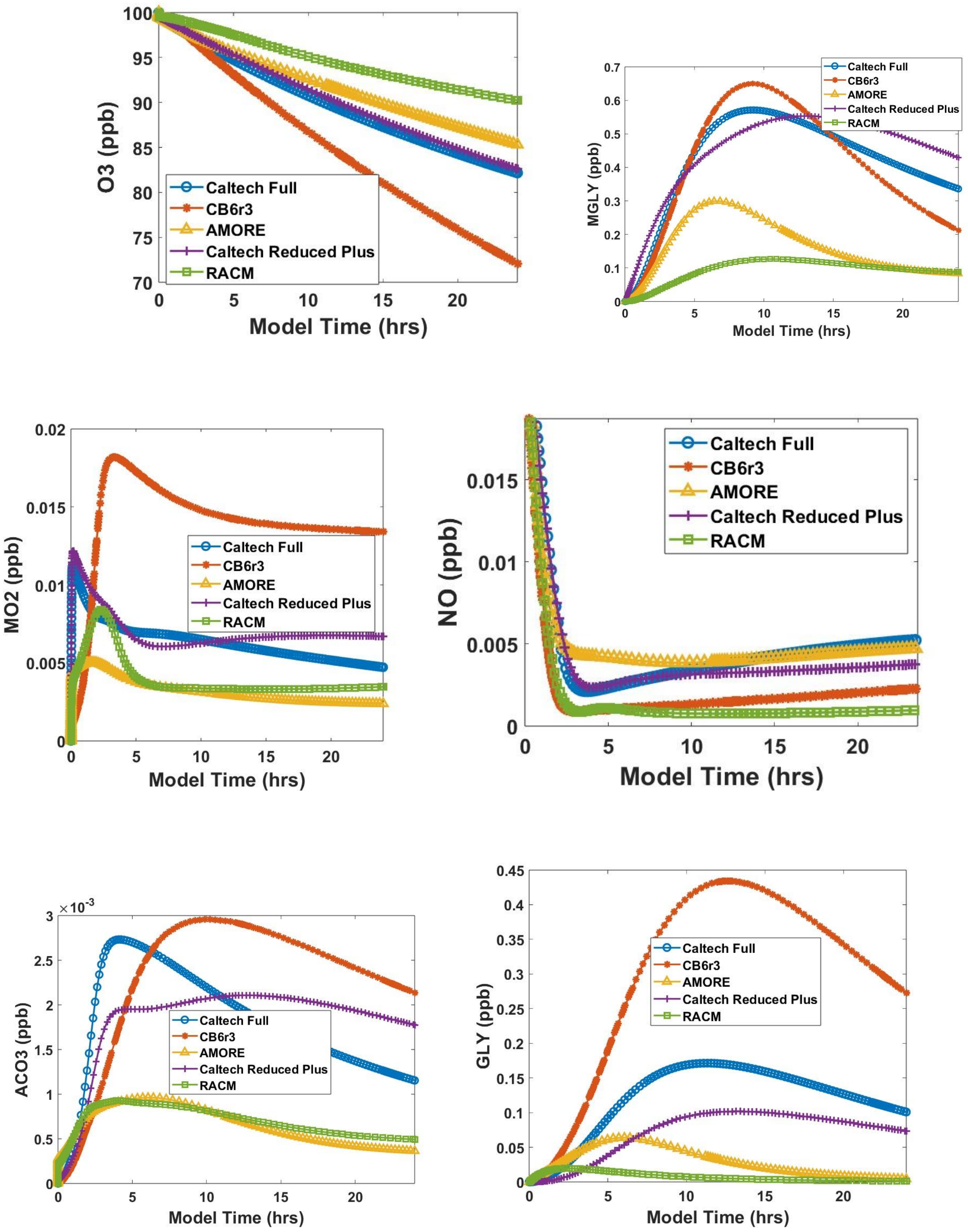

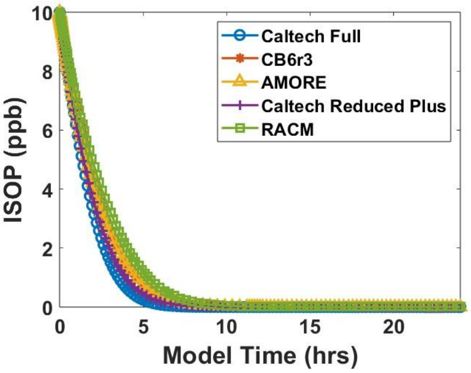

Figure S19. Chamber Comparison

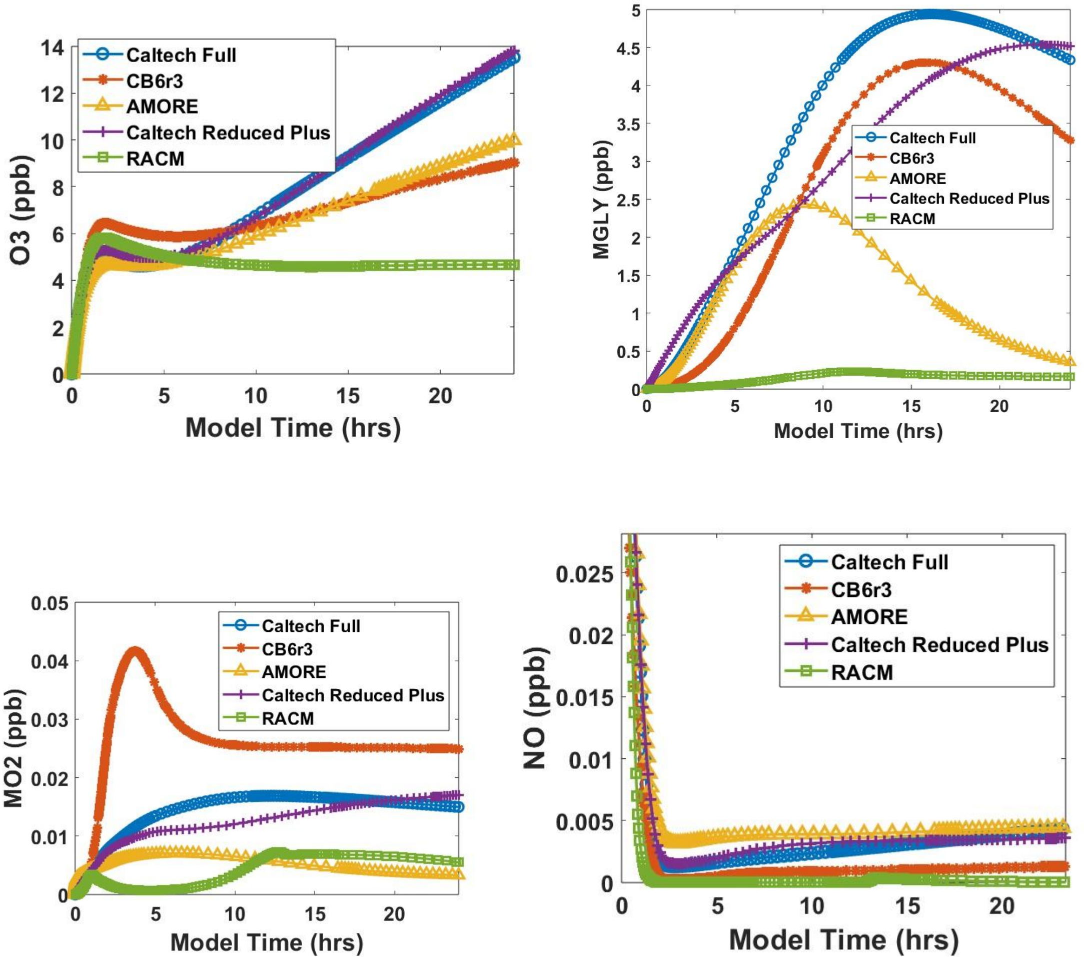

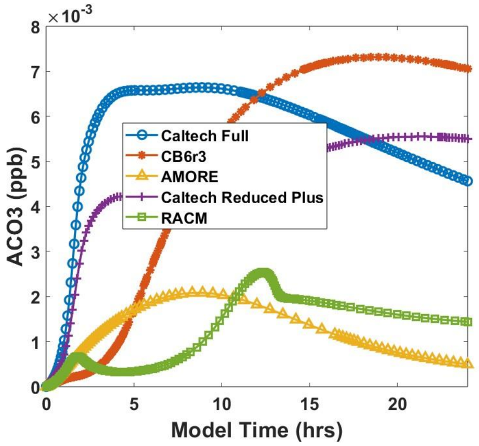

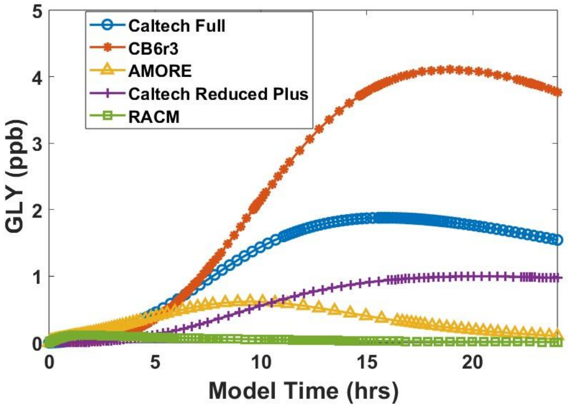

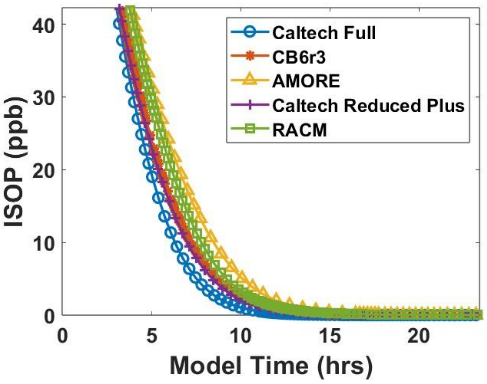

S18. Extended Caltech Isoprene Mechanism

This section lists out reactions that have been added to the Caltech full isoprene mechanism. These additions were made to improve the accuracy of the model at predicting concentrations of reactive oxidants and nitrogen radicals, and common end product species such as formaldehyde, glyoxal, and carbon monoxide. There were two types of changes made, and all changes were added reactions. No reactions were removed from the original mechanism.

The first type of change was to add degradation pathways for highly oxidized isoprene species. This was done by matching all species to their lumped counterparts in the caltech reduced mechanism. Any species that did not contain degradation pathways in the caltech full mechanism, but did have degradation pathways in the caltech reduced mechanism, were assigned identical degradation pathways.

Here is an example for the species "ISOP1N2N3OH4OH":

1.0ISOP1N2N3OH4OH = 2.0NO2 + 1.0GLYC + 1.0HAC : SUN*5.0E-6*2 ;

In cases in which the degradation product was a lumped species, the products were split up evenly between each of the lumped species. Here is an example for the species "ISOP1OH2OOH3OH4N":

1.0OH + 1.0ISOP1OH2OOH3OH4N = 0.33OH + 0.67HO2 + 0.165ISOP1OH2N3CO4OH + 0.165ISOP1OH2OH3N4CO + 0.0291ISOP1CO2N3OOH4OH + 0.0291ISOP1CO2OOH3N4OH + 0.0291ISOP1OH2OOH3N4CO + 0.0291ISOP1OH2N3OOH4CO + 0.0291ISOP1CO2N3OOH4OOH + 0.0291ISOP1CO2OOH3N4OOH + 0.0291ISOP1CO2OOH3OOH4N + 0.0291ISOP1OOH2OOH3N4CO + 0.0291ISOP1OOH2N3OOH4CO + 0.0291ISOP1N2OOH3OOH4CO + 0.0291ISOP1N2OOH3OH4CO + 0.0291ISOP1CO2OH3OOH4N + 0.0291ISOP1OOH2OH3CO4N + 0.0291ISOP1OOH2OH3N4CO + 0.0291ISOP1N2OH3CO4OOH + 0.0291ISOP1OH2OOH3CO4N + 0.0291ISOP1N4CO4OOH + 0.0291ISOP1N4CO4OH + 0.0291ISOP1CO1OOH4N + 0.0291ISOP1CO1OH4N + 0.0291ISOP1N2OH3OOH4CO + 0.0291ISOP1CO2OOH3OH4N + 0.0291ISOP1CO2N3OH4OOH : 3.0E-12 ;

The file titled 'lumped_species_correspondence' in the 'Caltech Full Mechanism (AMORE update)' folder, shows the matching between lumped species in the reduced mechanism, and non-lumped species in the full mechanism.

The second type of change was to add degradation chemistry for species without the isoprene structure. These species were generally smaller compounds (1-3 carbons). The degradation chemistry was obtained from the MCM mechanism and inserted into this mechanism. Below is a list of these species:

ETHLN, PYRAC, CH3CO3, PROPNN, CH3CO3, NCH2CO3, PNAN, PHAN, NCH2CO3H, CH3COOCH2, NPA, HOCH2COCHO, HOCH2COCHO, HOCH2CO3, NO3CH2CO2H, NPAH, NO3CH2PAN, HOCH2CO2H, HOCH2CO3H, HAC

Below is a list of every reaction in the extended mechanism. All new reactions are italicized.

#Equations
ISOP + OH = ISOP1OHc : 2.7E-11*EXP(390./TEMP)*0.63*0.5;
ISOP + OH = ISOP1OHt : 2.7E-11*EXP(390./TEMP)*0.63*0.5;
ISOP + OH = ISOP4OHc : 2.7E-11*EXP(390./TEMP)*0.37*0.7;

ISOP + OH = ISOP4OHt : 2.7E-11*EXP(390./TEMP)*0.37*0.3;
ISOP1OHt + O2 = ISOP1OH4OOt : 3.6E-13;
ISOP1OHt + O2 = ISOP1OH2OO : 7.5E-13;
ISOP1OHc + O2 = ISOP1OH2OO : 7.5E-13;
ISOP1OHc + O2 = ISOP1OH4OOc : 1.4E-13;
ISOP4OHt + O2 = ISOP1OO4OHt : 4.9E-13;
ISOP4OHt + O2 = ISOP3OO4OH : 6.5E-13;
ISOP4OHc + O2 = ISOP3OO4OH : 6.5E-13;
ISOP4OHc + O2 = ISOP1OO4OHc : 2.1E-13;
ISOP1OH4OOt = ISOP1OHt : 1.83E14*EXP(-8930./TEMP);
ISOP1OH2OO = ISOP1OHt : 2.22E15*EXP(-10355./TEMP);
ISOP1OH2OO = ISOP1OHc : 2.24E15*EXP(-10865./TEMP);
ISOP1OH4OOc = ISOP1OHc : 1.79E14*EXP(-8830./TEMP);
ISOP1OO4OHt = ISOP4OHt : 2.08E14*EXP(-9400./TEMP);
ISOP3OO4OH = ISOP4OHt : 2.49E15*EXP(-10890./TEMP);
ISOP3OO4OH = ISOP4OHc : 2.49E15*EXP(-11112./TEMP);
ISOP1OO4OHc = ISOP4OHc : 1.75E14*EXP(-9054./TEMP);
ISOP1OH2OO + NO = NO2 + MVK + HO2 + HCHO : ALK(2.7E-12,350.,1.190,6.,1.,0.);
ISOP1OH2OO + NO = ISOP1OH2N : NIT(2.7E-12,350.,1.190,6.,1.,0.);
ISOP1OH4OOc + NO = NO2 + HO2 + ISOP1CO4OH : ALK(1.215E-12,350.,1.421,6.,1.,0.);
ISOP1OH4OOc + NO = NO2 + ISOP1CO2OO3OOH4OH : ALK(1.485E-12,350.,1.421,6.,1.,0.);
ISOP1OH4OOc + NO = ISOP1OH4Nc : NIT(2.7E-12,350.,1.421,6.,1.,0.);
ISOP1OH4OOt + NO = NO2 + HO2 + ISOP1CO4OH : ALK(1.215E-12,350.,1.421,6.,1.,0.);
ISOP1OH4OOt + NO = NO2 + ISOP1CO2OO3OOH4OH : ALK(1.485E-12,350.,1.421,6.,1.,0.);
ISOP1OH4OOt + NO = ISOP1OH4Nt: NIT(2.7E-12,350.,1.421,6.,1.,0.);
ISOP1CO2OO3OOH4OH = ISOP1CO2OOH3OO4OH : TUN(4.21E13,8.93E3,1.55E8);
ISOP1CO2OOH3OO4OH = ISOP1CO2OO3OOH4OH : TUN(5.45E13,9.45E3,1.55E8);
ISOP1CO2OO3OOH4OH + NO = NO2 + MGLY + GLYC + OH : ALK(2.7E-12,350.,28.237,9.,1.,0.);
ISOP1CO2OO3OOH4OH + NO = ISOP1CO2N3OOH4OH : NIT(2.7E-12,350.,28.237,9.,1.,0.);
ISOP1CO2OO3OOH4OH = MVK3OOH4OH + CO + OH : TUN(4.92E12,9.89E3,9.66E7);
ISOP1CO2OO3OOH4OH + HO2 = 2OH + GLYC + MGLY : 2.47E-13*EXP(1300./TEMP);
ISOP1CO2OOH3OO4OH + NO = NO2 + MGLY + GLYC + OH : ALK(2.7E-12,350.,6.469,9.,1.,0.);
ISOP1CO2OOH3OO4OH + NO = ISOP1CO2OOH3N4OH : NIT(2.7E-12,350.,6.469,9.,1.,0.);
ISOP1CO2OOH3OO4OH = MVK3OOH4OH + CO + OH : TUN(2.28E13,1.08E4,1.24E8);
ISOP1CO2OOH3OO4OH + HO2 = ISOP1CO2OOH3OOH4OH : 0.25*2.47E-13*EXP(1300./TEMP);
ISOP1CO2OOH3OO4OH + HO2 = 2OH + GLYC + MGLY : 0.75*2.47E-13*EXP(1300./TEMP);
ISOP3OO4OH + NO = NO2 + MACR + HO2 + HCHO: ALK(2.7E-12,350.,1.297,6.,1.,0.);
ISOP3OO4OH + NO = ISOP3N4OH : NIT(2.7E-12,350.,1.297,6.,1.,0.);
ISOP1OO4OHc + NO = NO2 + HO2 + ISOP1OH4CO : ALK(1.215E-12,350.,1.421,6.,1.,0.);
ISOP1OO4OHc + NO = NO2 + ISOP1OH2OOH3OO4CO : ALK(1.485E-12,350.,1.421,6.,1.,0.);
ISOP1OO4OHc + NO = ISOP1N4OHc : NIT(2.7E-12,350.,1.421,6.,1.,0.);

ISOP1OO4OHt + NO = NO2 + HO2 + ISOP1OH4CO : ALK(1.215E-12,350.,1.421,6.,1.,0.);
ISOP1OO4OHt + NO = NO2 + ISOP1OH2OOH3OO4CO : ALK(1.485E-12,350.,1.421,6.,1.,0.);
ISOP1OO4OHt + NO = ISOP1N4OHt : NIT(2.7E-12,350.,1.421,6.,1.,0.);
ISOP1OH2OOH3OO4CO = ISOP1OH2OO3OOH4CO : TUN(1.34E13,9.18E3,1.64E8);
ISOP1OH2OO3OOH4CO = ISOP1OH2OOH3OO4CO : TUN(4.80E13,9.48E3,1.64E8);
ISOP1OH2OOH3OO4CO + NO = NO2 + GLYX + HAC + OH : ALK(2.7E-12,350.,35.396,9.,1.,0.);
ISOP1OH2OOH3OO4CO + NO = ISOP1OH2OOH3N4CO : NIT(2.7E-12,350.,35.396,9.,1.,0.);
ISOP1OH2OOH3OO4CO = MACR2OOH3OH + CO + OH : TUN(3.60E13,1.12E4,9.86E7);
ISOP1OH2OOH3OO4CO + HO2 = ISOP1OH2OOH3OOH4CO : 0.25*2.47E-13*EXP(1300./TEMP);
ISOP1OH2OOH3OO4CO + HO2 = 2OH + GLYX + HAC : 0.75*2.47E-13*EXP(1300./TEMP);
ISOP1OH2OO3OOH4CO + NO = NO2 + GLYX + HAC + OH : ALK(2.7E-12,350.,5.095,9.,1.,0.);
ISOP1OH2OO3OOH4CO + NO = ISOP1OH2N3OOH4CO : NIT(2.7E-12,350.,5.095,9.,1.,0.);
ISOP1OH2OO3OOH4CO = MACR2OOH3OH + CO + OH : TUN(3.67E12,9.91E3,1.21E8);
ISOP1OH2OO3OOH4CO + HO2 = ISOP1OH2OOH3OOH4CO : 0.15*2.47E-13*EXP(1300./TEMP);
ISOP1OH2OO3OOH4CO + HO2 = 2OH + GLYX + HAC : 0.85*2.47E-13*EXP(1300./TEMP);
ISOP1CO4OH + OH = ISOP1CO2OO3OH4OH : 2.1E-12*EXP(650./TEMP);
ISOP1CO4OH + OH = MVK3OOH4OH + CO + HO2 : 0.9E-12*EXP(650./TEMP);
ISOP1CO4OH + OH = ISOP1CO1R4OH : 3.8E-12*EXP(400./TEMP);
ISOP1OH4CO + OH = MACR2OOH3OH + HO2 + CO : 2.1E-12*EXP(650./TEMP);
ISOP1OH4CO + OH = ISOP1OH2OH3OO4CO : 0.9E-12*EXP(650./TEMP);
ISOP1OH4CO + OH = ISOP1OH4CO4R : 3.8E-12*EXP(400./TEMP);
ISOP1CO1R4OH + O2 = ISOP1CO1OO4OH : 1.0E-14 ;
ISOP1CO1OO4OH + NO2 = ISOP1PAN4OH : TROE(2.591E-28,0.,-6.87,1.125E-11,0.,-1.105,0.3);
ISOP1PAN4OH = ISOP1CO1OO4OH + NO2 : 1.58E16*EXP(-13500./TEMP);
ISOP1CO1OO4OH + NO = NO2 + CO2 + C4HVP1 : 2.7E-12*EXP(350./TEMP);
ISOP1CO1OO4OH + HO2 = OH + CO2 + C4HVP1 : 0.5*3.14E-12*EXP(580./TEMP);
ISOP1CO1OO4OH + HO2 = O3 + ISOP1CO1OH4OH : 0.13*3.14E-12*EXP(580./TEMP);
ISOP1CO1OO4OH + HO2 = ISOP1CO1OOH4OH : 0.37*3.14E-12*EXP(580./TEMP);
ISOP1CO1OO4OH = CO + OH + MACR2OOH3CO3OOH : 1.875E15*EXP(-10000./TEMP);
ISOP1OH4CO4R + O2 = ISOP1OH4CO4OO : 1.0E-14 ;
ISOP1OH4CO4OO + NO2 = ISOP1OH4PAN : TROE(2.591E-28,0.,-6.87,1.125E-11,0.,-1.105,0.3);
ISOP1OH4PAN = ISOP1OH4CO4OO + NO2 : 1.58E16*EXP(-13500./TEMP);
ISOP1OH4CO4OO + NO = NO2 + CO2 + C4HVP2 : 2.7E-12*EXP(350./TEMP);
ISOP1OH4CO4OO + HO2 = OH + CO2 + C4HVP2 : 0.5*3.14E-12*EXP(580./TEMP);
ISOP1OH4CO4OO + HO2 = ISOP1OH4CO4OH + O3 : 0.13*3.14E-12*EXP(580./TEMP);
ISOP1OH4CO4OO + HO2 = ISOP1OH4CO4OOH : 0.37*3.14E-12*EXP(580./TEMP);
ISOP1OH4CO4OO = CO + OH + MVK3OOH4CO4OOH : 1.875E15*EXP(-10000./TEMP);
ISOP3CO4OH + OH = ISOP1OH2OO3CO4OH : 2.7E-11*EXP(390./TEMP);
ISOP1OH2OO3CO4OH + HO2 = ISOP1OH2OOH3CO4OH : 0.35*2.38E-13*EXP(1300./TEMP);
ISOP1OH2OO3CO4OH + HO2 = OH + HO2 + HCHO + MVK3CO4OH :
0.13*2.38E-13*EXP(1300./TEMP);

ISOP1OH2OO3CO4OH + HO2 = OH + HAC + HPA : 0.52*2.38E-13*EXP(1300./TEMP);
ISOP1OH2OO3CO4OH + NO = ISOP1OH2N3CO4OH : NIT(2.7E-12,350.,13.098,8.,1.,0.);
ISOP1OH2OO3CO4OH + NO = NO2 + HAC + HPA : ALK(2.16E-12,350.,13.098,8.,1.,0.);
ISOP1OH2OO3CO4OH + NO = NO2 + HO2 + HCHO + MVK3CO4OH :
ALK(0.54E-12,350.,13.098,8.,1.,0.);
ISOP1OH2OO3CO4OH = HO2 + ISOP1OH2OOH3CO4CO : 1.875E13*EXP(-10000./TEMP);
ISOP1OH2OO + ISOP1OH2OO = MVK + MVK + HO2 + HO2 + HCHO + HCHO: 6.92E-14;
ISOP3OO4OH + ISOP3OO4OH = MACR + MACR + HO2 + HO2 + HCHO + HCHO: 5.74E-12*0.8;
ISOP3OO4OH + ISOP3OO4OH = ISOP3CO4OH + ISOP3OH4OH : 5.74E-12*0.2;
ISOP1OH2OO + ISOP3OO4OH = MVK + MACR + HO2 + HO2 + HCHO + HCHO: 3.08E-12*0.9;
ISOP1OH2OO + ISOP3OO4OH = ISOP1OH2OH + ISOP3CO4OH : 3.08E-12*0.1;
ISOP1OH2OO + ISOP1OO4OHc = MVK + HO2 + HCHO + HO2 + ISOP1OH4CO : 2.49E-12*0.805*0.45;
ISOP1OH2OO + ISOP1OO4OHc = MVK + HO2 + HCHO + ISOP1OH2OOH3OO4CO :
2.49E-12*0.805*0.55;
ISOP1OH2OO + ISOP1OO4OHc = ISOP1OH2OH + ISOP1CO4OH : 2.49E-12*(1.-0.805);
ISOP1OH2OO + ISOP1OO4OHt = MVK + HO2 + HCHO + HO2 + ISOP1OH4CO : 2.49E-12*0.805*0.45;
ISOP1OH2OO + ISOP1OO4OHt = MVK + HO2 + HCHO + ISOP1OH2OOH3OO4CO :
2.49E-12*0.805*0.55;
ISOP1OH2OO + ISOP1OO4OHt = ISOP1OH2OH + ISOP1CO4OH : 2.49E-12*(1.-0.805);
ISOP1OH2OO + ISOP1OH4OOc = MVK + HO2 + HCHO + HO2 + ISOP1CO4OH : 2.49E-12*0.805*0.45;
ISOP1OH2OO + ISOP1OH4OOc = MVK + HO2 + HCHO + ISOP1CO2OO3OOH4OH :
2.49E-12*0.805*0.5;
ISOP1OH2OO + ISOP1OH4OOc = ISOP1OH2OH + ISOP1OH4CO : 2.49E-12*(1.-0.805);
ISOP1OH2OO + ISOP1OH4OOt = MVK + HO2 + HCHO + HO2 + ISOP1CO4OH : 2.49E-12*0.805*0.45;
ISOP1OH2OO + ISOP1OH4OOt = MVK + HO2 + HCHO + ISOP1CO2OO3OOH4OH :
2.49E-12*0.805*0.55;
ISOP1OH2OO + ISOP1OH4OOt = ISOP1OH2OH + ISOP1OH4CO : 2.49E-12*(1.-0.805);
ISOP3OO4OH + ISOP1OO4OHc = MACR + HO2 + HCHO + HO2 + ISOP1OH4CO :
3.94E-12*0.705*0.45;
ISOP3OO4OH + ISOP1OO4OHc = MACR + HO2 + HCHO + ISOP1OH2OOH3OO4CO :
3.94E-12*0.705*0.55;
ISOP3OO4OH + ISOP1OO4OHc = ISOP3OH4OH + ISOP1CO4OH : 3.94E-12*(1.-0.705)*0.5;
ISOP3OO4OH + ISOP1OO4OHc = ISOP1OH4OH + ISOP3CO4OH : 3.94E-12*(1.-0.705)*0.5;
ISOP3OO4OH + ISOP1OO4OHt = MACR + HO2 + HCHO + HO2 + ISOP1OH4CO :
3.94E-12*0.705*0.45;
ISOP3OO4OH + ISOP1OO4OHt = MACR + HO2 + HCHO + ISOP1OH2OOH3OO4CO :
3.94E-12*0.705*0.55;
ISOP3OO4OH + ISOP1OO4OHt = ISOP3OH4OH + ISOP1CO4OH : 3.94E-12*(1.-0.705)*0.5;
ISOP3OO4OH + ISOP1OO4OHt = ISOP1OH4OH + ISOP3CO4OH : 3.94E-12*(1.-0.705)*0.5;
ISOP3OO4OH + ISOP1OH4OOc = MACR + HO2 + HCHO + HO2 + ISOP1CO4OH :
3.94E-12*0.705*0.45;

ISOP3OO4OH + ISOP1OH4OOc = MACR + HO2 + HCHO + ISOP1CO2OO3OOH4OH :
3.94E-12*0.705*0.55;
ISOP3OO4OH + ISOP1OH4OOc = ISOP3OH4OH + ISOP1OH4CO : 3.94E-12*(1.-0.705)*0.5;
ISOP3OO4OH + ISOP1OH4OOc = ISOP1OH4OH + ISOP3CO4OH : 3.94E-12*(1.-0.705)*0.5;
ISOP3OO4OH + ISOP1OH4OOt = MACR + HO2 + HCHO + HO2 + ISOP1CO4OH :
3.94E-12*0.705*0.45;
ISOP3OO4OH + ISOP1OH4OOt = MACR + HO2 + HCHO + ISOP1CO2OO3OOH4OH :
3.94E-12*0.705*0.55;
ISOP3OO4OH + ISOP1OH4OOt = ISOP3OH4OH + ISOP1OH4CO : 3.94E-12*(1.-0.705)*0.5;
ISOP3OO4OH + ISOP1OH4OOt = ISOP1OH4OH + ISOP3CO4OH : 3.94E-12*(1.-0.705)*0.5;
ISOP1OH2OO + CH3OO = MVK + 2HO2 + 2HCHO : 2.00E-12*0.5;
ISOP1OH2OO + CH3OO = ISOP1OH2OH + HCHO : 2.00E-12*0.5;
ISOP3OO4OH + CH3OO = MACR + 2HO2 + 2HCHO : 2.00E-12*0.5;
ISOP3OO4OH + CH3OO = ISOP3OH4OH + HCHO : 2.00E-12*0.25;
ISOP3OO4OH + CH3OO = ISOP3CO4OH + CH3OH : 2.00E-12*0.25;
ISOP1OH4OOc + CH3OO = ISOP1OH4OH + HCHO : 2.00E-12*0.5;
ISOP1OH4OOc + CH3OO = ISOP1CO2OO3OOH4OH + HCHO + HO2 : 2.00E-12*0.5*0.55;
ISOP1OH4OOc + CH3OO = 2HO2 + ISOP1CO4OH + HCHO : 2.00E-12*0.5*0.45;
ISOP1OH4OOt + CH3OO = ISOP1OH4OH + HCHO : 2.00E-12*0.5;
ISOP1OH4OOt + CH3OO = ISOP1CO2OO3OOH4OH + HCHO + HO2 : 2.00E-12*0.5*0.55;
ISOP1OH4OOt + CH3OO = 2HO2 + ISOP1CO4OH + HCHO : 2.00E-12*0.5*0.45;
ISOP1OO4OHc + CH3OO = ISOP1OH4OH + HCHO : 2.00E-12*0.5;
ISOP1OO4OHc + CH3OO = ISOP1OH2OOH3OO4CO + HCHO + HO2 : 2.00E-12*0.5*0.55;
ISOP1OO4OHc + CH3OO = 2HO2 + ISOP1OH4CO + HCHO : 2.00E-12*0.5*0.45;
ISOP1OO4OHt + CH3OO = ISOP1OH4OH + HCHO : 2.00E-12*0.5;
ISOP1OO4OHt + CH3OO = ISOP1OH2OOH3OO4CO + HCHO + HO2 : 2.00E-12*0.5*0.55;
ISOP1OO4OHt + CH3OO = 2HO2 + ISOP1OH4CO + HCHO : 2.00E-12*0.5*0.45;
HO2 + ISOP1OH2OO = ISOP1OH2OOH + O2 : 2.12E-13*EXP(1300./TEMP)*0.937;
HO2 + ISOP1OH2OO = MVK + OH + HO2 + HCHO : 2.12E-13*EXP(1300./TEMP)*0.063;
HO2 + ISOP3OO4OH = ISOP3OOH4OH + O2 : 2.12E-13*EXP(1300./TEMP)*0.937;
HO2 + ISOP3OO4OH = MACR + OH + HO2 + HCHO : 2.12E-13*EXP(1300./TEMP)*0.063;
HO2 + ISOP1OO4OHc = ISOP1OOH4OHc + O2 : 2.12E-13*EXP(1300./TEMP);
HO2 + ISOP1OO4OHt = ISOP1OOH4OHt + O2 : 2.12E-13*EXP(1300./TEMP);
HO2 + ISOP1OH4OOc = ISOP1OH4OOHc + O2 : 2.12E-13*EXP(1300./TEMP);
HO2 + ISOP1OH4OOt = ISOP1OH4OOHt + O2 : 2.12E-13*EXP(1300./TEMP);
ISOP1OH4OOc = ISOP1CO4OOHc + HO2 : TUN(1.26E15,12200.,1.0E8);
ISOP1OH4OOc = ISOP1CO2OOH + HO2 : TUN(7.58E14,12200.,1.0E8);
ISOP1OH4OOc = ISOP1CO2R3OOH4OOH : TUN(3.03E15,12200.,1.0E8);
ISOP1CO2R3OOH4OOH + O2 = ISOP1CO2OO3OOH4OOH : 1.0E-14 ;
ISOP1CO2R3OOH4OOH = ISOP1CO23O4OOH : 2.7E3 ;
ISOP1CO2OO3OOH4OOH = ISOP1CO2OOH3OO4OOH : TUN(1.72E13,7.79E3,1.73E8);

ISOP1CO2OO3OOH4OOH = ISOP1CO2OOH3OOH4OO : TUN(4.63E11,5.09E3,7.83E7);
ISOP1CO2OOH3OO4OOH = ISOP1CO2OO3OOH4OOH : TUN(7.79E12,8.19E3,1.73E8);
ISOP1CO2OOH3OO4OOH = ISOP1CO2OOH3OOH4OO : TUN(2.41E13,8.83E3,1.60E8);
ISOP1CO2OOH3OOH4OO = ISOP1CO2OO3OOH4OOH : TUN(4.14E11,5.84E3,7.83E7);
ISOP1CO2OOH3OOH4OO = ISOP1CO2OOH3OO4OOH : TUN(9.75E12,9.18E3,1.60E8);
ISOP1CO2OO3OOH4OOH = ISOP1CO2OOH3OOH4CO + OH : 9.375E12*EXP(-10000./TEMP);
ISOP1CO2OO3OOH4OOH = MVK3OOH4OOH + OH + CO : TUN(4.33E12,1.00E4,1.14E8);
ISOP1CO2OOH3OO4OOH = MVK3OOH4OOH + OH + CO : TUN(2.59E12,1.02E4,1.10E8);
ISOP1CO2OOH3OOH4OO = MVK3OOH4OOH + OH + CO : TUN(1.69E12,9.43E3,9.14E7);
ISOP1CO2OO3OOH4OOH + NO = ISOP1CO2N3OOH4OOH : NIT(2.7E-12,350.,35.468,10.,1.,0.);
ISOP1CO2OO3OOH4OOH + NO = NO2 + CO + HO2 + MVK3OOH4OOH :
ALK(0.54E-12,350.,35.468,10.,1.,0.);
ISOP1CO2OO3OOH4OOH + NO = NO2 + OH + MGLY + HPETHNL :
ALK(2.16E-12,350.,35.468,10.,1.,0.);
ISOP1CO2OO3OOH4OOH + HO2 = OH + OH + MGLY + HPETHNL : 0.8*2.54E-13*EXP(1300./TEMP);
ISOP1CO2OO3OOH4OOH + HO2 = OH + CO + HO2 + MVK3OOH4OOH :
0.2*2.54E-13*EXP(1300./TEMP);
ISOP1CO2OOH3OO4OOH + NO = ISOP1CO2OOH3N4OOH : NIT(2.7E-12,350.,13.849,10.,1.,0.);
ISOP1CO2OOH3OO4OOH + NO = NO2 + CO + HO2 + MVK3OOH4OOH :
ALK(0.54E-12,350.,13.849,10.,1.,0.);
ISOP1CO2OOH3OO4OOH + NO = NO2 + OH + MGLY + HPETHNL :
ALK(2.16E-12,350.,13.849,10.,1.,0.);
ISOP1CO2OOH3OO4OOH + HO2 = OH + OH + MGLY + HPETHNL : 0.6*2.54E-13*EXP(1300./TEMP);
ISOP1CO2OOH3OO4OOH + HO2 = OH + CO + HO2 + MVK3OOH4OOH :
0.15*2.54E-13*EXP(1300./TEMP);
ISOP1CO2OOH3OO4OOH + HO2 = ISOP1CO2OOH3OOH4OOH : 0.25*2.54E-13*EXP(1300./TEMP);
ISOP1CO2OOH3OOH4OO + NO = ISOP1CO2OOH3OOH4N : NIT(2.7E-12,350.,5.555,10.,1.,0.);
ISOP1CO2OOH3OOH4OO + NO = OH + NO2 + HCHO + MACR2OOH3CO :
ALK(2.7E-12,350.,5.555,10.,1.,0.);
ISOP1CO2OOH3OOH4OO + HO2 = ISOP1CO2OOH3OOH4OOH : 0.8*2.54E-13*EXP(1300./TEMP);
ISOP1CO2OOH3OOH4OO + HO2 = OH + OH + HCHO + MACR2OOH3CO :
0.2*2.54E-13*EXP(1300./TEMP);
ISOP1OO4OHc = ISOP1OOH4COc + HO2 : TUN(5.55E8,7160.,1.0E8);
ISOP1OO4OHc = ISOP3OOH4CO + HO2 : TUN(3.33E8,7160.,1.0E8);
ISOP1OO4OHc = ISOP1OOH2OOH3R4CO : TUN(1.33E9,7160.,1.0E8);
ISOP1OOH2OOH3R4CO + O2 = ISOP1OOH2OOH3OO4CO : 1.0E-14 ;
ISOP1OOH2OOH3R4CO = ISOP1OOH23O4CO : 2.7E3 ;
ISOP1OOH2OOH3OO4CO = ISOP1OOH2OO3OOH4CO : TUN(9.10E12,8.39E3,1.43E8);
ISOP1OOH2OOH3OO4CO = ISOP1OO2OOH3OOH4CO : TUN(3.92E13,6.60E3,8.75E7);
ISOP1OOH2OO3OOH4CO = ISOP1OOH2OOH3OO4CO : TUN(5.49E11,7.70E3,1.43E8);
ISOP1OOH2OO3OOH4CO = ISOP1OO2OOH3OOH4CO : TUN(4.24E12,7.92E3,1.67E8);

ISOP1OO2OOH3OOH4CO = ISOP1OOH2OOH3OO4CO : TUN(5.61E12,7.16E3,8.75E7);
ISOP1OO2OOH3OOH4CO = ISOP1OOH2OO3OOH4CO : TUN(2.38E13,9.16E3,1.67E8);
ISOP1OOH2OOH3OO4CO = ISOP1CO2OOH3OOH4CO + OH : 9.375E12*EXP(-10000./TEMP);
ISOP1OO2OOH3OOH4CO = ISOP1OOH2OOH3CO4CO + OH : 1.875E13*EXP(-10000./TEMP);
ISOP1OOH2OOH3OO4CO = MACR2OOH3OOH + OH + CO : TUN(4.86E13,1.14E4,1.38E8);
ISOP1OOH2OO3OOH4CO = MACR2OOH3OOH + OH + CO : TUN(2.42E11,9.44E3,1.06E8);
ISOP1OO2OOH3OOH4CO = MACR2OOH3OOH + OH + CO : TUN(6.68E12,9.60E3,9.30E7);
ISOP1OOH2OOH3OO4CO + NO = ISOP1OOH2OOH3N4CO : NIT(2.7E-12,350.,44.441,10.,1.,0.);
ISOP1OOH2OOH3OO4CO + NO = NO2 + CO + HO2 + MACR2OOH3OOH :
ALK(0.54E-12,350.,44.441,10.,1.,0.);
ISOP1OOH2OOH3OO4CO + NO = NO2 + OH + GLYX + HPAC : ALK(2.16E-12,350.,44.441,10.,1.,0.);
ISOP1OOH2OOH3OO4CO + HO2 = HO2 + OH + GLYX + HPAC : 0.6*2.54E-13*EXP(1300./TEMP);
ISOP1OOH2OOH3OO4CO + HO2 = HO2 + CO + HO2 + MACR2OOH3OOH :
0.15*2.54E-13*EXP(1300./TEMP);
ISOP1OOH2OOH3OO4CO + HO2 = ISOP1OOH2OOH3OOH4CO : 0.25*2.54E-13*EXP(1300./TEMP);
ISOP1OOH2OO3OOH4CO + NO = ISOP1OOH2N3OOH4CO : NIT(2.7E-12,350.,10.994,10.,1.,0.);
ISOP1OOH2OO3OOH4CO + NO = NO2 + CO + HO2 + MACR2OOH3OOH :
ALK(0.54E-12,350.,10.994,10.,1.,0.);
ISOP1OOH2OO3OOH4CO + NO = NO2 + OH + GLYX + HPAC : ALK(2.16E-12,350.,10.994,10.,1.,0.);
ISOP1OOH2OO3OOH4CO + HO2 = HO2 + OH + GLYX + HPAC : 0.80*2.54E-13*EXP(1300./TEMP);
ISOP1OOH2OO3OOH4CO + HO2 = HO2 + CO + HO2 + MACR2OOH3OOH :
0.20*2.54E-13*EXP(1300./TEMP);
ISOP1OO2OOH3OOH4CO + NO = ISOP1N2OOH3OOH4CO : NIT(2.7E-12,350.,5.555,10.,1.,0.);
ISOP1OO2OOH3OOH4CO + NO = NO2 + OH + HCHO + MVK3OOH4CO :
ALK(2.7E-12,350.,5.555,10.,1.,0.);
ISOP1OO2OOH3OOH4CO + HO2 = ISOP1OOH2OOH3OOH4CO : 0.8*2.54E-13*EXP(1300./TEMP);
ISOP1OO2OOH3OOH4CO + HO2 = OH + OH + HCHO + MVK3OOH4CO :
0.2*2.54E-13*EXP(1300./TEMP);
MVK3OOH4OOH = MGLY + OH + OH + HCHO : SUN*3.0E-5;
MVK3OOH4OOH = HPETHNL + CH3CO3 + OH : SUN*3.0E-5;
MACR2OOH3OOH = MGLY + OH + OH + HCHO : SUN*3.0E-5;
MACR2OOH3OOH = HPAC + CO + OH + HO2 : SUN*3.0E-5;
ISOP1CO2OOH + OH = CO + OH + MVK : 3.8E-12*EXP(400./TEMP) ;
ISOP1CO2OOH + OH = CO + OH + MVK : 2.0E-12*EXP(200./TEMP) ;
ISOP1CO2OOH + OH = ISOP1CO23O4OH + OH : 0.75*1.7E-11*EXP(390./TEMP) ;
ISOP1CO2OOH + OH = ISOP1CO2OOH3OH4OO : 0.25*1.7E-11*EXP(390./TEMP) ;
ISOP1CO2OOH = CO + OH + HO2 + MVK : SUN*3.0E-5 ;
ISOP3OOH4CO + OH = CO + OH + MACR : 3.8E-12*EXP(400./TEMP) ;
ISOP3OOH4CO + OH = CO + OH + MACR : 2.0E-12*EXP(200./TEMP) ;
ISOP3OOH4CO + OH = ISOP1OH23O4CO + OH : 0.9*3.0E-11*EXP(390./TEMP) ;
ISOP3OOH4CO + OH = ISOP1OO2OH3OOH4CO : 0.1*3.0E-11*EXP(390./TEMP) ;

ISOP3OOH4CO = CO + OH + HO2 + MACR : SUN*3.0E-5 ;

ISOP1OH2OO = HCHO + OH + MVK : 1.04E11*EXP(-9746./TEMP);

ISOP3OO4OH = MACR + OH + HCHO : 1.88E11*EXP(-9752./TEMP);

MVK + OH = MVK3OO4OH : 2.6E-12*EXP(610./TEMP)*0.75;

MVK + OH = MVK3OH4OO : 2.6E-12*EXP(610./TEMP)*0.25;

MVK3OO4OH + HO2 = CH3CO3 + GLYC + OH : 2.12E-13*EXP(1300./TEMP)*0.48;

MVK3OO4OH + HO2 = MVK3CO4OH + OH + HO2 : 2.12E-13*EXP(1300./TEMP)*0.34;

MVK3OO4OH + HO2 = MVK3OOH4OH : 2.12E-13*EXP(1300./TEMP)*0.18;

MVK3OH4OO + HO2 = MVK3OH4OOH : 2.12E-13*EXP(1300./TEMP)*0.8;

MVK3OH4OO + HO2 = MGLY + HCHO + OH + HO2 : 2.12E-13*EXP(1300./TEMP)*0.2;

MVK3OOH4OH = CH3CO3 + GLYC + OH : SUN*3.0E-5;

MVK3OO4OH + NO = CH3CO3 + GLYC + NO2 : ALK(2.7E-12,350.,6.161,6.,1.,0.);

MVK3OO4OH + NO = MVK3N4OH : NIT(2.7E-12,350.,6.161,6.,1.,0.);

MVK3OH4OO + NO = MGLY + HCHO + HO2 + NO2 : ALK(2.7E-12,350.,2.531,6.,1.,0.);

MVK3OH4OO + NO = MVK3OH4N : NIT(2.7E-12,350.,2.531,6.,1.,0.);

MVK3N4OH = CH3CO3 + GLYC + NO2 : SUN*6.46E-5;

MVK3OH4N = CH3CO3 + ETHLN + HO2 : SUN*4.21E-5;

MACR + OH = MACR2OO3OH : 8.0E-12*EXP(380./TEMP)*0.53;

MACR + OH = MACR2OH3OO : 8.0E-12*EXP(380./TEMP)*0.02;

MACR + OH = MACR1OO : 2.7E-12*EXP(470./TEMP);

MACR2OO3OH + HO2 = MACR2OOH3OH : 2.12E-13*EXP(1300./TEMP)*0.41;

MACR2OO3OH + HO2 = HAC + CO + HO2 + OH : 2.12E-13*EXP(1300./TEMP)*0.59*0.86;

MACR2OO3OH + HO2 = MGLY + HCHO + OH : 2.12E-13*EXP(1300./TEMP)*0.59*0.14;

MACR2OH3OO + HO2 = MACR2OH3OOH : 2.12E-13*EXP(1300./TEMP)*0.80;

MACR2OH3OO + HO2 = MGLY + HCHO + HO2 + OH : 2.12E-13*EXP(1300./TEMP)*0.20;

MACR1OO + HO2 = MACR1OOH : 3.14E-12*EXP(580./TEMP)*0.37;

MACR1OO + HO2 = CH3COOCH2 + CO2 + OH : 3.14E-12*EXP(580./TEMP)*0.50;

MACR1OO + HO2 = MACR1OH + O3 : 3.14E-12*EXP(580./TEMP)*0.13;

MACR2OO3OH + NO = HAC + CO + HO2 + NO2 : ALK(2.322E-12,350.,2.985,6.,1.,0.);

MACR2OO3OH + NO = MGLY + HCHO + NO2 : ALK(0.378E-12,350.,2.985,6.,1.,0.);

MACR2OO3OH + NO = MACR2N3OH : NIT(2.7E-12,350.,2.985,6.,1.,0.);

MACR2OH3OO + NO = MGLY + HCHO + HO2 + NO2 : ALK(2.7E-12,350.,2.985,6.,1.,0.);

MACR2OH3OO + NO = MACR2OH3N : NIT(2.7E-12,350.,2.985,6.,1.,0.);

MACR1OO + NO = CH3COOCH2 + CO2 + NO2 : 8.7E-12*EXP(290./TEMP);

MACR1OO + NO2 = MPAN : TROE(2.591E-28,0.,-6.87,1.125E-11,0.,-1.105,0.3);

MPAN = MACR1OO + NO2 : 1.58E16*EXP(-13500./TEMP);

MACR2OO3OH = HAC + CO + OH : 2.9E7*EXP(-5297./TEMP);

MACR2OH3OO = HPAC + CO + HO2 : 4.0E8*EXP(-5000./TEMP);

MPAN + OH = MPAN1OHx : 2.9E-11;

MPAN1OHx = MPAN1OH2OO : 1.0E7;

MPAN1OHx = HMMLx : 4.0E9;

MPAN1OHx = MPAN1OH : 8.18E7;
MPAN1OH = HMMLx : 1.0E3;
MPAN1OH = MPAN1OH2OO : 1.0E7;
HMMLx = HMML + NO3 : 1.0E8*0.75;
HMMLx = HAC + CO + NO3 : 1.0E8*0.25;
MPAN1OH2OO + NO = MPAN1OH2O + NO2 : 2.7E-12*EXP(350./TEMP);
MPAN1OH2OO + HO2 = MPAN1OH2OOH : 2.6E-13*EXP(1300./TEMP)*0.1;
MPAN1OH2OO + HO2 = MPAN1OH2O + OH : 2.6E-13*EXP(1300./TEMP)*0.9;
MPAN1OH2O = HAC + CO2 + NO3 : 1.0E8;
ISOP1OH2OOH + OH = ISOP1OH2OOH3R4OH : 1.7E-11*EXP(390./TEMP)*0.95;
ISOP1OH2OOH3R4OH + O2 = ISOP1OH2OOH3OO4OH : 1.0E-14;
ISOP1OH2OOH3R4OH = ISOP1OH23O4OHt + OH : 4.4E5*0.67;
ISOP1OH2OOH3R4OH = ISOP1OH23O4OHc + OH : 4.4E5*0.33;
ISOP3OOH4OH + OH = ISOP1OH2R3OOH4OH : 3.0E-11*EXP(390./TEMP)*0.95;
ISOP1OH2R3OOH4OH + O2 = ISOP1OH2OO3OOH4OH : 1.0E-14;
ISOP1OH2R3OOH4OH = ISOP1OH23O4OHt + OH : 4.4E5*0.68;
ISOP1OH2R3OOH4OH = ISOP1OH23O4OHc + OH : 4.4E5*0.32;
ISOP1OH2OOH + OH = ISOP1OH2OOH3OH4OO : 1.7E-11*EXP(390./TEMP)*0.05;
ISOP1OH2OOH3OH4OO = ISOP1OH2OO3OH4OOH : TUN(1.68E12,5.36E3,8.24E7);
ISOP1OH2OO3OH4OOH = ISOP1OH2OOH3OH4OO : TUN(1.39E13,6.14E3,8.24E7);
ISOP1OH2OOH3OH4OO + NO = MACR2OOH3OH + NO2 + HO2 + HCHO :
ALK(2.7E-12,350.,2.478,9.,1.,0.);
ISOP1OH2OOH3OH4OO + NO = ISOP1OH2OOH3OH4N : NIT(2.7E-12,350.,2.478,9.,1.,0.);
ISOP1OH2OO3OH4OOH + NO = HPETHNL + HAC + NO2 + HO2 : ALK(2.7E-12,350.,1.518,9.,1.,0.);
ISOP1OH2OO3OH4OOH + NO = ISOP1OH2N3OH4OOH : NIT(2.7E-12,350.,1.518,9.,1.,0.);
ISOP1OH2OOH3OH4OO + HO2 = ISOP1OH2OOH3OH4OOH : 0.80*2.47E-13*EXP(1300./TEMP);
ISOP1OH2OOH3OH4OO + HO2 = MACR2OOH3OH + OH + HO2 + HCHO :
0.20*2.47E-13*EXP(1300./TEMP);
ISOP1OH2OO3OH4OOH + HO2 = ISOP1OH2OOH3OH4OOH : 0.55*2.47E-13*EXP(1300./TEMP);
ISOP1OH2OO3OH4OOH + HO2 = HPETHNL + HAC + OH + HO2 : 0.45*2.47E-13*EXP(1300./TEMP);
ISOP1OH2OO3OH4OOH = ISOP1OH2OOH3OH4CO + OH : TUN(9.93E12,1.26E4,8.10E7);
ISOP1OH2OOH3OH4OO = ISOP1OH12O3OH4OOH + OH : TUN(2.84E12,9.82E3,8.57E7);
ISOP3OOH4OH + OH = ISOP1OO2OH3OOH4OH : 3.0E-11*EXP(390./TEMP)*0.05;
ISOP1OO2OH3OOH4OH = ISOP1OOH2OH3OO4OH : TUN(4.11E11,5.43E3,7.45E7);
ISOP1OOH2OH3OO4OH = ISOP1OO2OH3OOH4OH : TUN(9.33E11,5.29E3,7.45E7);
ISOP1OO2OH3OOH4OH + NO = MVK3OOH4OH + HO2 + HCHO + NO2 :
ALK(2.7E-12,350.,2.478,9.,1.,0.);
ISOP1OO2OH3OOH4OH + NO = ISOP1N2OH3OOH4OH : NIT(2.7E-12,350.,2.478,9.,1.,0.);
ISOP1OOH2OH3OO4OH + NO = HPAC + GLYC + NO2 + HO2 : ALK(2.7E-12,350.,1.998,9.,1.,0.);
ISOP1OOH2OH3OO4OH + NO = ISOP1OOH2OH3N4OH : NIT(2.7E-12,350.,1.998,9.,1.,0.);
ISOP1OO2OH3OOH4OH + HO2 = ISOP1OOH2OH3OOH4OH : 0.80*2.47E-13*EXP(1300./TEMP);

ISOP1OO2OH3OOH4OH + HO2 = MVK3OOH4OH + HO2 + HCHO + OH : 0.20*2.47E-13*EXP(1300./TEMP);
ISOP1OOH2OH3OO4OH + HO2 = ISOP1OOH2OH3OOH4OH : 0.55*2.47E-13*EXP(1300./TEMP);
ISOP1OOH2OH3OO4OH + HO2 = HPAC + GLYC + OH + HO2 : 0.45*2.47E-13*EXP(1300./TEMP);
ISOP1OO2OH3OOH4OH = ISOP1OOH2OH3CO4OH + OH : TUN(1.94E12,1.09E4,1.17E8);
ISOP1OO2OH3OOH4OH = ISOP1OOH2OH34O4OH + OH : TUN(9.07E11,9.60E3,8.44E7);
ISOP1OOH2OH3OO4OH = ISOP1CO2OH3OOH4OH + OH : TUN(7.69E12,1.11E4,9.22E7);
ISOP1OH2OOH3OO4OH = ISOP1OH2OO3OOH4OH : TUN(1.06E13,8.37E3,1.57E8);
ISOP1OH2OO3OOH4OH = ISOP1OH2OOH3OO4OH : TUN(1.21E13,8.76E3,1.57E8);
ISOP1OH2OOH3OO4OH + NO = ISOP1OH2OOH3N4OH : NIT(2.7E-12,350.,3.576,9.,1.,0.);
ISOP1OH2OOH3OO4OH + NO = GLYC + HAC + NO2 + OH : ALK(2.7E-12,350.,3.576,9.,1.,0.);
ISOP1OH2OOH3OO4OH + HO2 = GLYC + HAC + 2OH : 0.65*2.47E-13*EXP(1300./TEMP);
ISOP1OH2OOH3OO4OH + HO2 = ISOP1OH2OOH3OOH4OH : 0.35*2.47E-13*EXP(1300./TEMP);
ISOP1OH2OOH3OO4OH = ISOP1OH12O3OOH4OH + OH : TUN(7.85E11,9.18E3,7.78E7);
ISOP1OH2OO3OOH4OH + NO = ISOP1OH2N3OOH4OH : NIT(2.7E-12,350.,2.781,9.,1.,0.);
ISOP1OH2OO3OOH4OH + NO = HAC + GLYC + NO2 + OH : ALK(2.7E-12,350.,2.781,9.,1.,0.);
ISOP1OH2OO3OOH4OH + HO2 = ISOP1OH2OOH3OOH4OH : 0.35*2.47E-13*EXP(1300./TEMP);
ISOP1OH2OO3OOH4OH + HO2 = HAC + GLYC + 2OH : 0.65*2.47E-13*EXP(1300./TEMP);
ISOP1OH2OO3OOH4OH = ISOP1OH2OOH34O4OH + OH : TUN(2.01E12,9.65E3,8.05E7);
ISOP1OH2OOH + OH = ISOP1OH2OO : 4.6E-12*EXP(200./TEMP);
ISOP1OH2OOH + OH = ISOP1CO2OOH + HO2 : 1.5E-12*EXP(200./TEMP)*0.5;
ISOP1OH2OOH + OH = ISO1OH12O + OH : 1.5E-12*EXP(200./TEMP)*0.5;
ISOP3OOH4OH + OH = ISOP3OO4OH : 2.1E-12*EXP(200./TEMP);
ISOP3OOH4OH + OH = ISOP3CO4OH + OH : 2.0E-12*EXP(200./TEMP)*0.32;
ISOP3OOH4OH + OH = ISOP3OOH4CO + HO2 : 2.0E-12*EXP(200./TEMP)*0.68;
ISOP1OH2OOH = MVK + HCHO + HO2 + OH : SUN*6.5E-6;
ISOP3OOH4OH = MACR + HCHO + HO2 + OH : SUN*6.5E-6;
ISOP1OOH4OHt = OH + HO2 + ISOP1CO4OH : SUN*6.49E-6;
ISOP1OH4OOHt = OH + HO2 + ISOP1OH4CO : SUN*6.49E-6;
ISOP1OOH4OHc = OH + HO2 + ISOP1CO4OH : SUN*0.4*6.49E-6;
ISOP1OOH4OHc = OH + OH + CO + MACR2OOH3OH : SUN*0.6*6.49E-6;
ISOP1OH4OOHc = OH + HO2 + ISOP1OH4CO : SUN*0.4*6.49E-6;
ISOP1OH4OOHc = OH + OH + CO + MVK3OOH4OH : SUN*0.6*6.49E-6;
ISOP1OH4OOHc + OH = ISOP1OH2OO3OH4OOH : 2.1E-11*EXP(390./TEMP);
ISOP1OH4OOHc + OH = ISOP1OH2OH34O + OH : 0.9E-11*EXP(390./TEMP);
ISOP1OH4OOHc + OH = ISOP1OH4OOc : 2.0E-12*EXP(200./TEMP);
ISOP1OH4OOHc + OH = ISOP1OH4CO + OH : 7.5E-12*EXP(20./TEMP);
ISOP1OH4OOHc + OH = ISOP1CO2OOH + HO2 : 0.15*7.5E-12*EXP(20./TEMP);
ISOP1OH4OOHc + OH = ISOP1CO4OOHc + HO2 : 0.25*7.5E-12*EXP(20./TEMP);
ISOP1OH4OOHc + OH = ISOP1CO2R3OOH4OOH : 0.60*7.5E-12*EXP(20./TEMP);
ISOP1OH4OOHt + OH = ISOP1OH2OO3OH4OOH : 2.1E-11*EXP(390./TEMP);

ISOP1OH4OOHt + OH = ISOP1OH2OH34O + OH : 0.9E-11*EXP(390./TEMP);
ISOP1OH4OOHt + OH = ISOP1OH4OOt : 2.0E-12*EXP(200./TEMP);
ISOP1OH4OOHt + OH = ISOP1OH4CO + OH : 7.5E-12*EXP(20./TEMP);
ISOP1OH4OOHt + OH = ISOP1CO2OOH + HO2 : 0.15*7.5E-12*EXP(20./TEMP);
ISOP1OH4OOHt + OH = ISOP1CO4OOHc + HO2 : 0.25*7.5E-12*EXP(20./TEMP);
ISOP1OH4OOHt + OH = ISOP1CO2R3OOH4OOH : 0.60*7.5E-12*EXP(20./TEMP);
ISOP1OOH4OHc + OH = ISOP1OOH2OH3OO4OH : 0.9E-11*EXP(390./TEMP);
ISOP1OOH4OHc + OH = ISOP12O3OH4OH + OH : 2.1E-11*EXP(390./TEMP);
ISOP1OOH4OHc + OH = ISOP1OO4OHc : 2.0E-12*EXP(200./TEMP);
ISOP1OOH4OHc + OH = ISOP1CO4OH + OH : 7.5E-12*EXP(20./TEMP);
ISOP1OOH4OHc + OH = ISOP3OOH4CO + HO2 : 0.15*7.5E-12*EXP(20./TEMP);
ISOP1OOH4OHc + OH = ISOP1OOH4COc + HO2 : 0.25*7.5E-12*EXP(20./TEMP);
ISOP1OOH4OHc + OH = ISOP1OOH2OOH3R4CO : 0.60*7.5E-12*EXP(20./TEMP);
ISOP1OOH4OHt + OH = ISOP1OOH2OH3OO4OH : 0.9E-11*EXP(390./TEMP);
ISOP1OOH4OHt + OH = ISOP12O3OH4OH + OH : 2.1E-11*EXP(390./TEMP);
ISOP1OOH4OHt + OH = ISOP1OO4OHt : 2.0E-12*EXP(200./TEMP);
ISOP1OOH4OHt + OH = ISOP1CO4OH + OH : 7.5E-12*EXP(20./TEMP);
ISOP1OOH4OHt + OH = ISOP3OOH4CO + HO2 : 0.15*7.5E-12*EXP(20./TEMP);
ISOP1OOH4OHt + OH = ISOP1OOH4COc + HO2 : 0.25*7.5E-12*EXP(20./TEMP);
ISOP1OOH4OHt + OH = ISOP1OOH2OOH3R4CO : 0.60*7.5E-12*EXP(20./TEMP);
ISOP1OH23O4OHc + OH = ISOP1OH23O4R4OHc : 0.19*5.82E-11*EXP(-400./TEMP);
ISOP1OH23O4R4OHc + O2 = ISOP1OH23O4CO + H2O + HO2 : 1.0E-14;
ISOP1OH23O4R4OHc = ISOP1OH2OH3OO4CO : 1.835E5;
ISOP1OH23O4OHc + OH = ISOP1OH1R23O4OHc : 0.81*5.82E-11*EXP(-400./TEMP);
ISOP1OH1R23O4OHc + O2 = ISOP1CO23O4OH + H2O + HO2 : 1.0E-14;
ISOP1OH1R23O4OHc = ISOP1CO2OO3OH4OH : 1.835E5;
ISOP1OH23O4OHt + OH = ISOP1OH23O4R4OHt : 0.33*3.75E-11*EXP(-400./TEMP);
ISOP1OH23O4R4OHt + O2 = ISOP1OH23O4CO + H2O + HO2 : 1.0E-14;
ISOP1OH23O4R4OHt = ISOP1OH2OH3OO4CO : 2.358E5;
ISOP1OH23O4OHt + OH = ISOP1OH1R23O4OHt : 0.67*3.75E-11*EXP(-400./TEMP);
ISOP1OH1R23O4OHt + O2 = ISOP1CO23O4OH + H2O + HO2 : 1.0E-14;
ISOP1OH1R23O4OHt = ISOP1CO2OO3OH4OH : 2.358E5;
ISOP1OH2OH34O + OH = ISOP1CO2OH34O + HO2 : 3.22E-11*EXP(-400./TEMP);
ISOP12O3OH4OH + OH = ISOP12O3OH4CO + HO2 : 0.2*3.22E-11*EXP(-400./TEMP);
ISOP12O3OH4OH + OH = ISOP12O3OH3R4OH : 0.8*3.22E-11*EXP(-400./TEMP);
ISOP12O3OH3R4OH + O2 = ISOP12O3CO4OH + HO2 : 0.33*1.0E-14;
ISOP12O3OH3R4OH + O2 = ISOP1OH3OH4CO + HO2 : 0.33*1.0E-14;
ISOP12O3OH3R4OH + O2 = ISOP1CO3OH4OH + HO2 : 0.33*1.0E-14;
ISOP12O3OH3R4OH = ISOP1OH2OO3CO4OH : 3.45E4;
ISOP1OH2OH3OO4CO = ISOP1CO2OH3OOH4CO + HO2 : 1.875E13*EXP(-10000./TEMP);
ISOP1OH2OH3OO4CO = CO + OH + MACR2OH3OH : 1.0E7*EXP(-5000./TEMP);

ISOP1OH2OH3OO4CO + NO = CO + NO2 + HO2 + MACR2OH3OH :
ALK(0.5625E-12,350.,16.463,8.,1.,0.);
ISOP1OH2OH3OO4CO + NO = NO2 + HO2 + GLYX + HAC : ALK(2.1375E-12,350.,16.463,8.,1.,0.);
ISOP1OH2OH3OO4CO + NO = ISOP1OH2OH3N4CO : NIT(2.7E-12,350.,16.463,8.,1.,0.);
ISOP1OH2OH3OO4CO + HO2 = CO + OH + HO2 + MACR2OH3OH : 0.13*2.38E-13*EXP(1300./TEMP);
ISOP1OH2OH3OO4CO + HO2 = OH + HO2 + GLYX + HAC : 0.52*2.38E-13*EXP(1300./TEMP);
ISOP1OH2OH3OO4CO + HO2 = ISOP1OH2OH3OOH4CO : 0.35*2.38E-13*EXP(1300./TEMP);
ISOP1CO2OO3OH4OH = ISOP1CO2OOH3OH4CO + HO2 : 1.875E13*EXP(-10000./TEMP);
ISOP1CO2OO3OH4OH = OH + CO + MVK3OH4OH : 1.0E7*EXP(-5000./TEMP);
ISOP1CO2OO3OH4OH + HO2 = CO + OH + HO2 + MVK3OH4OH : 0.13*2.38E-13*EXP(1300./TEMP);
ISOP1CO2OO3OH4OH + HO2 = OH + HO2 + GLYC + MGLY : 0.52*2.38E-13*EXP(1300./TEMP);
ISOP1CO2OO3OH4OH + HO2 = ISOP1CO2OOH3OH4OH : 0.35*2.38E-13*EXP(1300./TEMP);
ISOP1CO2OO3OH4OH + NO = MVK3OH4OH + HO2 + NO2 + CO :
ALK(0.5625E-12,350.,13.098,8.,1.,0.);
ISOP1CO2OO3OH4OH + NO = NO2 + HO2 + GLYC + MGLY : ALK(2.1375E-12,350.,13.098,8.,1.,0.);
ISOP1CO2OO3OH4OH + NO = ISOP1CO2N3OH4OH : NIT(2.7E-12,350.,13.098,8.,1.,0.);
MVK3OH4OH + OH = MVK3OH4CO + HO2 : 0.4*8.7E-12*EXP(70./TEMP);
MVK3OH4OH + OH = MVK3CO4OH + HO2 : 0.6*8.7E-12*EXP(70./TEMP);
MVK3OH4CO + OH = OH + MGLY + CO2 : 5.0E-12*EXP(470./TEMP);
MVK3OH4CO = CO + HO2 + HO2 + MGLY : 0.5*2.5E-4*SUN;
MVK3OH4CO = GLYX + HO2 + CH3CO3 : 0.5*2.5E-4*SUN;
MVK3CO4OH + OH = CO + CO + HO2 + CH3CO3 : 2E-12*EXP(70./TEMP);
MVK3CO4OH = CO + HO2 + HCHO + CH3CO3 : 2.5E-4*SUN;
MACR2OH3OH + OH = MACR2OH3CO + HO2 : 0.16*2.4E-11*EXP(70./TEMP);
MACR2OH3OH + OH = 0.25HAC + HO2 + CO + 0.75CO2 + 0.75OH + 0.75CH3CO3 :
0.84*2.4E-11*EXP(70./TEMP);
MACR2OH3CO + OH = CO2 + OH + CO + HO2 + CH3CO3 : 5.0E-12*EXP(470./TEMP);
ISOP1CO4OOHc = MVKENOL + OH + OH + CO : 0.552*0.58*SUN*4.0E-4;
ISOP1CO4OOHc = C4HVP1 + OH + CO : 0.224*0.58*SUN*4.0E-4;
ISOP1CO4OOHc = ISOP1CO4CO + HO2 + OH : 0.112*0.58*SUN*4.0E-4;
ISOP1CO4OOHc = ISOP1CO2OO3OOH4CO + OH : 0.112*0.58*SUN*4.0E-4;
ISOP1OOH4COc = MACRENOL + OH + OH + CO : 0.455*0.55*SUN*4.0E-4;
ISOP1OOH4COc = C4HVP2 + OH + CO : 0.182*0.55*SUN*4.0E-4;
ISOP1OOH4COc = ISOP1CO4CO + HO2 + OH : 0.182*0.55*SUN*4.0E-4;
ISOP1OOH4COc = ISOP1CO2OOH3OO4CO + OH : 0.182*0.55*SUN*4.0E-4;
ISOP1CO2OO3OOH4CO = ISOP1CO2OOH3OO4CO : 3.0E6;
ISOP1CO2OOH3OO4CO = ISOP1CO2OO3OOH4CO : 4.0E6;
ISOP1CO2OO3OOH4CO = MACR2OOH3CO + OH + CO : 4.0E8*EXP(-5000./TEMP);
ISOP1CO2OO3OOH4CO = MVK3OOH4CO + OH + CO : 1.0E7*EXP(-5000./TEMP);
ISOP1CO2OOH3OO4CO = MACR2OOH3CO + OH + CO : 1.0E7*EXP(-5000./TEMP);
ISOP1CO2OOH3OO4CO = MVK3OOH4CO + OH + CO : 4.0E8*EXP(-5000./TEMP);

MVKENOL + OH = HO2 + MVK3OH4CO : 0.25*3.35E-12*EXP(983./TEMP);

MVKENOL + OH = MVK3OO4OH4OH : 0.75*3.35E-12*EXP(983./TEMP);

MVKENOL = CH3CO3 + GLYX + OH : 0.5*2.5E-4*SUN;

MVKENOL = MGLY + HO2 + CO + OH : 0.5*2.5E-4*SUN;

MVK3OO4OH4OH + NO = MVK3N4OH4OH : NIT(2.7E-12,350.,17.402,7.,1.,0.);

MVK3OO4OH4OH + NO = NO2 + HO2 + HCOOH + MGLY : ALK(2.7E-12,350.,17.402,7.,1.,0.);

MVK3OO4OH4OH + HO2 = OH + HO2 + HCOOH + MGLY : 0.7*2.26E-13*EXP(1300./TEMP);

MVK3OO4OH4OH + HO2 = MVK3OOH4OH4OH : 0.3*2.26E-13*EXP(1300./TEMP);

MACRENOL + OH = DHA + CO + OH : 3.35E-12*EXP(983./TEMP);

MACRENOL + OH = PPYRAC + OH + CO : 2.7E-12*EXP(470./TEMP);

DHA + OH = HO2 + PYRAC : 8.0E-12*EXP(70./TEMP);

MACRENOL = MACR3CO + OH + OH : 2.5E-4*SUN;

MACR3CO + OH = PYRAC + OH + CO : 2.7E-11*EXP(390./TEMP);

C4HVP1 + NO = NO2 + MVK3OO4OH : 2.7E-12*EXP(350./TEMP);

C4HVP1 + HO2 = OH + MVK3OO4OH : 1.93E-13*EXP(1300./TEMP);

C4HVP1 + NO2 = MVK3N4OH : 9.0E-12;

C4HVP2 + NO = NO2 + MACR2OO3OH : 2.7E-12*EXP(350./TEMP);

C4HVP2 + HO2 = OH + MACR2OO3OH : 1.93E-13*EXP(1300./TEMP);

C4HVP2 + NO2 = MACR2N3OH : 9.0E-12;

MACR2OOH3CO = OH + MGLY + HO2 + CO : 2.5E-4*SUN;

MACR2OOH3CO + OH = OH + CO + MGLY : 5.0E-12*EXP(470./TEMP);

MVK3OOH4CO = OH + GLYX + CH3CO3 : 2.5E-4*SUN;

MVK3OOH4CO + OH = OH + CO + MGLY : 5.0E-12*EXP(470./TEMP);

ISOP1CO4OOHc + OH = ISOP1CO4CO + OH : 7.5E-12*EXP(20./TEMP);

ISOP1CO4OOHc + OH = ISOP1CO4OOc : 2.0E-12*EXP(200./TEMP);

ISOP1CO4OOHc + OH = ISOP1CO2OO3OH4OOH : 2.1E-12*EXP(650./TEMP);

ISOP1CO4OOHc + OH = ISOP1CO2OH3R4OOH : 0.9E-12*EXP(650./TEMP);

ISOP1CO2OH3R4OOH + O2 = ISOP1CO2OH3OO4OOH : 1.0E-14;

ISOP1CO2OH3R4OOH = ISOP1CO2OH34O : 5.2E4;

ISOP1CO4OOHc + OH = ISOP1CO3R4OOH : 3.8E-12*EXP(400./TEMP);

ISOP1CO3R4OOH + O2 = ISOP1CO1OO4OOH : 1.0E-14;

ISOP1CO2OH3OO4OOH = MVK3OOH4OOH + CO + HO2 : 4.0E8*EXP(-5000./TEMP);

ISOP1CO2OH3OOH4OO = MVK3OOH4OOH + CO + HO2 : 1.0E8*EXP(-5000./TEMP);

ISOP1CO2OH3OO4OOH = ISOP1CO2OH3OOH4OO : 2.0E6;

ISOP1CO2OH3OOH4OO = ISOP1CO2OH3OO4OOH : 3.0E6;

ISOP1CO2OO3OH4OOH = ISOP1CO2OOH3OH4CO + OH : 9.375E12*EXP(-10000./TEMP);

ISOP1CO2OO3OH4OOH = MVK3OH4OOH + CO + OH : 1.0E7*EXP(-5000./TEMP);

ISOP1CO2OOH3OH4OO = MVK3OH4OOH + CO + OH : 1.0E8*EXP(-5000./TEMP);

ISOP1CO2OO3OH4OOH = ISOP1CO2OOH3OH4OO : 2.0E6;

ISOP1CO2OOH3OH4OO = ISOP1CO2OO3OH4OOH : 4.0E6;

ISOP1CO4OOc = ISOP1CO2OO : 10.0;

ISOP1CO2OO = MVK + OH + CO : 1.0E7*EXP(-5000./TEMP);
ISOP1CO4OOc = ISOP1CO3R4OOH : 10.0;
ISOP1CO1OO4OOH = ISOP1CO1OOH4OO : 1.0E6;
ISOP1CO1OOH4OO = ISOP1CO1OOH : 1.79E14*EXP(-8830./TEMP);
ISOP1CO1OOH + O2 = ISOP1CO1OOH2OO : 7.5E-13;
ISOP1CO1OOH2OO + NO = MVK + CO2 + NO2 + OH : 2.7E-12*EXP(350./TEMP);
ISOP1CO1OOH2OO + HO2 = MVK + CO2 + OH + OH : 2.38E-13*EXP(1300./TEMP);
ISOP1OOH4COc + OH = ISOP1CO4CO + OH : 7.5E-12*EXP(20./TEMP);
ISOP1OOH4COc + OH = ISOP1OO4COc : 2.0E-12*EXP(200./TEMP);
ISOP1OOH4COc + OH = ISOP1OOH2OH3OO4CO : 0.9E-12*EXP(650./TEMP);
ISOP1OOH4COc + OH = ISOP1OOH2R3OH4CO : 2.1E-12*EXP(650./TEMP);
ISOP1OOH2R3OH4CO + O2 = ISOP1OOH2OO3OH4CO : 1.0E-14;
ISOP1OOH2R3OH4CO = ISOP12O3OH4CO : 5.2E4;
ISOP1OOH4COc + OH = ISOP1OOH2R4CO : 3.8E-12*EXP(400./TEMP);
ISOP1OOH2R4CO + O2 = ISOP1OOH4CO4OO : 1.0E-14;
ISOP1OOH2OH3OO4CO = ISOP1CO2OH3OOH4CO + OH : 9.375E12*EXP(-10000./TEMP);
ISOP1OOH2OH3OO4CO = MACR2OH3OOH + OH + CO : 1.0E7*EXP(-5000./TEMP);
ISOP1OO2OH3OOH4CO = ISOP1OOH2OH3CO4CO + OH : 1.875E13*EXP(-10000./TEMP);
ISOP1OO2OH3OOH4CO = MACR2OH3OOH + OH + CO : 1.0E8*EXP(-5000./TEMP);
ISOP1OOH2OH3OO4CO = ISOP1OO2OH3OOH4CO : 2.0E6;
ISOP1OO2OH3OOH4CO = ISOP1OOH2OH3OO4CO : 3.0E6;
ISOP1OOH2OO3OH4CO = MACR2OOH3OOH + CO + HO2 : 4.0E8*EXP(-5000./TEMP);
ISOP1OO2OOH3OH4CO = MACR2OOH3OOH + CO + HO2 : 1.0E8*EXP(-5000./TEMP);
ISOP1OOH2OO3OH4CO = ISOP1OO2OOH3OH4CO : 2.0E6;
ISOP1OO2OOH3OH4CO = ISOP1OOH2OO3OH4CO : 4.0E6;
ISOP1OO4COc = ISOP3OO4CO : 10.0;
ISOP3OO4CO = MACR + CO + OH : 2.9E7*EXP(-5297/TEMP);
ISOP1OO4COc = ISOP1OOH2R4CO : 10.0;
ISOP1OOH4CO4OO = ISOP1OO4CO4OOH : 1.0E6;
ISOP1OO4CO4OOH = ISOP4CO4OOH : 1.75E14*EXP(-9054./TEMP);
ISOP4CO4OOH + O2 = ISOP3OO4CO4OOH : 6.5E-13;
ISOP3OO4CO4OOH + NO = MACR + CO2 + NO2 + OH : 2.7E-12*EXP(350./TEMP);
ISOP3OO4CO4OOH + HO2 = MACR + CO2 + OH + OH : 2.38E-13*EXP(1300./TEMP);
ISOP1OH2N + OH = ISOP1OH2N3R4OH : 0.75*8.4E-12*EXP(390./TEMP);
ISOP1OH2N3R4OH + O2 = ISOP1OH2N3OO4OH : 1.0E-14;
ISOP1OH2N3R4OH = ISOP1OH23O4OHt : 1.3E4*0.67;
ISOP1OH2N3R4OH = ISOP1OH23O4OHc : 1.3E4*0.33;
ISOP1OH2N + OH = ISOP1OH2N3OH4OO : 0.25*8.4E-12*EXP(390./TEMP);
ISOP1OH2N3OH4OO + NO = ISOP1OH2N3OH4N : NIT(2.7E-12,350.,2.660,11.,1.,0.);
ISOP1OH2N3OH4OO + NO = MACR2N3OH + HO2 + HCHO + NO2 : ALK(2.7E-12,350.,2.660,11.,1.,0.);
ISOP1OH2N3OO4OH + NO = ISOP1OH2N3N4OH : NIT(2.7E-12,350.,13.035,11.,1.,0.);

ISOP1OH2N3OO4OH + NO = GLYC + NO2 + NO2 + HAC : ALK(2.7E-12,350.,13.035,11.,1.,0.);
ISOP1OH2N3OO4OH = ISOP1CO2N3OOH4OH + HO2 : 1.875E13*EXP(-10000./TEMP);
ISOP1OH2N3OH4OO = ISOP1CO2N3OH4OOH + HO2 : 1.875E13*EXP(-10000./TEMP);
ISOP1OH2N3OH4OO + HO2 = ISOP1OH2N3OH4OOH : 0.8*2.6E-13*EXP(1300./TEMP);
ISOP1OH2N3OH4OO + HO2 = MACR2N3OH + HO2 + HCHO + OH : 0.2*2.6E-13*EXP(1300./TEMP);
ISOP1OH2N3OO4OH + HO2 = ISOP1OH2N3OOH4OH : 0.35*2.6E-13*EXP(1300./TEMP);
ISOP1OH2N3OO4OH + HO2 = GLYC + HAC + OH + NO2 : 0.65*2.6E-13*EXP(1300./TEMP);
ISOP3N4OH + OH = ISOP1OH2R3N4OH : 0.9*1.17E-11*EXP(390./TEMP);
ISOP1OH2R3N4OH + O2 = ISOP1OH2OO3N4OH : 1.0E-14;
ISOP1OH2R3N4OH = ISOP1OH23O4OHt : 0.67*8.42E3;
ISOP1OH2R3N4OH = ISOP1OH23O4OHc : 0.33*8.42E3;
ISOP3N4OH + OH = ISOP1OO2OH3N4OH : 0.1*1.17E-11*EXP(390./TEMP);
ISOP1OH2OO3N4OH + NO = ISOP1OH2N3N4OH : NIT(2.7E-12,350.,10.339,11.,1.,0.);
ISOP1OH2OO3N4OH + NO = MVK3N4OH + HO2 + NO2 + HCHO : ALK(2.7E-12,350.,10.339,11.,1.,0.);
ISOP1OO2OH3N4OH + NO = ISOP1N2OH3N4OH : NIT(2.7E-12,350.,2.660,11.,1.,0.);
ISOP1OO2OH3N4OH + NO = MVK3N4OH + HO2 + NO2 + HCHO : ALK(2.7E-12,350.,2.660,11.,1.,0.);
ISOP1OO2OH3N4OH = ISOP1OOH2OH3N4CO + HO2 : 1.875E13*EXP(-10000./TEMP);
ISOP1OH2OO3N4OH = ISOP1OH2OOH3N4CO + HO2 : 1.875E13*EXP(-10000./TEMP);
ISOP1OH2OO3N4OH + HO2 = MVK3N4OH + HO2 + OH + HCHO : 0.65*2.6E-13*EXP(1300./TEMP);
ISOP1OH2OO3N4OH + HO2 = ISOP1OH2OOH3N4OH : 0.35*2.6E-13*EXP(1300./TEMP);
ISOP1OO2OH3N4OH + HO2 = ISOP1OOH2OH3N4OH : 0.80*2.6E-13*EXP(1300./TEMP);
ISOP1OO2OH3N4OH + HO2 = MVK3N4OH + HO2 + OH + HCHO : 0.20*2.6E-13*EXP(1300./TEMP);
ISOP + O3 = MACR + ciCH2OO : 1.1E-14*EXP(-2000./TEMP)*0.41;
ISOP + O3 = MVK + ciCH2OO : 1.1E-14*EXP(-2000./TEMP)*0.17;
ISOP + O3 = OH : 1.1E-14*EXP(-2000./TEMP)*0.28;
ISOP + O3 = ciMVKOO : 1.1E-14*EXP(-2000./TEMP)*0.007;
ISOP + O3 = ciMACROO : 1.1E-14*EXP(-2000./TEMP)*0.006;
ISOP + O3 = HO2 : 1.1E-14*EXP(-2000./TEMP)*0.16;
ISOP + O3 = HCHO + CO2 + HCHO + CO + CH3OO : 1.1E-14*EXP(-2000./TEMP)*0.407;
ciCH2OO + SO2 = HCHO + H2SO4 : 3.7E-11;
ciCH2OO + HCOOH = HPMF : 2.0E-11;
ciCH2OO + H2O = HMHP : 1.7E-15*(0.73);
ciCH2OO + H2O = HCOOH : 1.7E-15*(0.21);
ciCH2OO + H2O = HCHO : 1.7E-15*(0.06);
ciCH2OO + H2O = H2O2 : 1.7E-15*(0.06);
ciCH2OO + H2Od = HMHP : 1.5E-12*(0.4);
ciCH2OO + H2Od = HCOOH : 1.5E-12*(0.54);
ciCH2OO + H2Od = HCHO : 1.5E-12*(0.06);
ciCH2OO + H2Od = H2O2 : 1.5E-12*(0.06);
ciCH2OO + O3 = HCHO : 2.0E-12*(0.7);
ciMACROO + H2O = MACR3OH3OOH : 1.0E-15;

ciMVKOO + H2O = MVK3OH3OOH : 1.0E-15;

HMHP + OH = HCHO + HO2 : 1.3E-12*EXP(500./TEMP)*0.5;

HMHP + OH = HCOOH + OH : 1.3E-12*EXP(500./TEMP)*0.5;

HPMF + OH = HO2 + FAH : 4.31E-12;

FAH + OH = CO + HO2 + CO2 : 1.80E-13;

ISOP + NO3 = ISOP1N2OO : 2.95E-12*EXP(-450./TEMP)*0.42;

ISOP + NO3 = ISOP3OO4N : 2.95E-12*EXP(-450./TEMP)*0.045;

ISOP + NO3 = ISOP1N4OO : 2.95E-12*EXP(-450./TEMP)*0.45;

ISOP + NO3 = ISOP1OO4N : 2.95E-12*EXP(-450./TEMP)*0.085;

ISOP1N2OO + HO2 = ISOP1N2OOH : 2.47E-13*EXP(1300./TEMP)*0.47;

ISOP1N2OO + HO2 = MVK + OH + HCHO + NO2 : 2.47E-13*EXP(1300./TEMP)*0.53;

ISOP1N4OO + HO2 = ISOP1N4OOH : 2.47E-13*EXP(1300./TEMP);

ISOP3OO4N + HO2 = ISOP3OOH4N : 2.47E-13*EXP(1300./TEMP)*0.5;

ISOP3OO4N + HO2 = MACR + OH + HCHO + NO2 : 2.47E-13*EXP(1300./TEMP)*0.5;

ISOP1OO4N + HO2 = ISOP1OOH4N : 2.47E-13*EXP(1300./TEMP);

ISOP1N2OO + ISOP1N2OO = MVK + HCHO + NO2 + MVK + HCHO + NO2 : 6.92E-14*0.965;

ISOP1N2OO + ISOP1N2OO = ISOP1N2OOISOP1N2 : 6.92E-14*0.035;

ISOP1N2OO + ISOP3OO4N = ISOP1N2OH + ISOP3CO4N : 3.08E-12*0.77*0.5 ;

ISOP1N2OO + ISOP3OO4N = MVK + MACR + HCHO + HCHO + NO2 + NO2 : 3.08E-12*0.58 ;

ISOP1N2OO + ISOP3OO4N = ISOP3OO4NISOP1N2 : 3.08E-12*0.035 ;

ISOP1N2OO + ISOP1OO4N = ISOP1N2OH + ISOP1CO4N : 2.49E-12*0.77*0.5 ;

ISOP1N2OO + ISOP1OO4N = MVK + HCHO + NO2 + ISOP1O4N : 2.49E-12*0.58 ;

ISOP1N2OO + ISOP1OO4N = ISOP1OO4NISOP1N2 : 2.49E-12*0.035 ;

ISOP1N2OO + ISOP1N4OO = ISOP1N4CO + ISOP1N2OH : 2.49E-12*0.77*0.5;

ISOP1N2OO + ISOP1N4OO = MVK + HCHO + NO2 + ISOP1N4O : 2.49E-12*0.58;

ISOP1N2OO + ISOP1N4OO = ISOP1N2OOISOP1N4 : 2.49E-12*0.035;

ISOP1N4OO + ISOP1N4OO = ISOP1N4CO + ISOP1N4OHc : 3.9E-12*0.77*0.5;

ISOP1N4OO + ISOP1N4OO = ISOP1N4CO + ISOP1N4OHt : 3.9E-12*0.77*0.5;

ISOP1N4OO + ISOP1N4OO = ISOP1N4O + ISOP1N4O : 3.9E-12*0.195;

ISOP1N4OO + ISOP1N4OO = ISOP1N4OOISOP1N4 : 3.9E-12*0.035;

ISOP3OO4N + ISOP1N4OO = ISOP3OH4N + ISOP1N4CO : 3.94E-12*0.77*0.5;

ISOP3OO4N + ISOP1N4OO = ISOP3CO4N + ISOP1N4OHc : 3.94E-12*0.77*0.5*0.5;

ISOP3OO4N + ISOP1N4OO = ISOP3CO4N + ISOP1N4OHt : 3.94E-12*0.77*0.5*0.5;

ISOP3OO4N + ISOP1N4OO = MACR + HCHO + NO2 + ISOP1N4O : 3.94E-12*0.195;

ISOP3OO4N + ISOP1N4OO = ISOP3OO4NISOP1N4 : 3.94E-12*0.035;

ISOP1OO4N + ISOP1N4OO = ISOP1CO4N + ISOP1N4OHc : 3.29E-12*0.77*0.5*0.5;

ISOP1OO4N + ISOP1N4OO = ISOP1CO4N + ISOP1N4OHt : 3.29E-12*0.77*0.5*0.5;

ISOP1OO4N + ISOP1N4OO = ISOP1OH4Nc + ISOP1N4CO : 3.29E-12*0.77*0.5*0.5;

ISOP1OO4N + ISOP1N4OO = ISOP1OH4Nt + ISOP1N4CO : 3.29E-12*0.77*0.5*0.5;

ISOP1OO4N + ISOP1N4OO = ISOP1O4N + ISOP1N4O : 3.29E-12*0.195;

ISOP1OO4N + ISOP1N4OO = ISOP1OO4NISOP1N4 : 3.29E-12*0.035;

ISOP1N2OO + CH3OO = HCHO + ISOP1N2OH : 1.6E-13*0.71*0.5;
ISOP1N2OO + CH3OO = MVK + 2HCHO + NO2 + HO2 : 1.6E-13*0.645;
ISOP1N4OO + CH3OO = ISOP1N4CO + CH3OH : 1.2E-12*0.71*0.5;
ISOP1N4OO + CH3OO = ISOP1N4OHc + HCHO : 1.2E-12*0.71*0.5*0.5;
ISOP1N4OO + CH3OO = ISOP1N4OHt + HCHO : 1.2E-12*0.71*0.5*0.5;
ISOP1N4OO + CH3OO = ISOP1N4O + HO2 + HCHO : 1.2E-12*0.29;
ISOP3OO4N + CH3OO = ISOP3OH4N + HCHO : 1.4E-12*0.71*0.5;
ISOP3OO4N + CH3OO = ISOP3CO4N + CH3OH : 1.4E-12*0.71*0.5;
ISOP3OO4N + CH3OO = MACR + 2HCHO + NO2 + HO2 : 1.4E-12*0.29;
ISOP1OO4N + CH3OO = ISOP1CO4N + CH3OH : 9.8E-13*0.71*0.5;
ISOP1OO4N + CH3OO = ISOP1OH4Nc + HCHO : 9.8E-13*0.71*0.5*0.5;
ISOP1OO4N + CH3OO = ISOP1OH4Nt + HCHO : 9.8E-13*0.71*0.5*0.5;
ISOP1OO4N + CH3OO = ISOP1O4N + HO2 + HCHO : 9.8E-13*0.29;
ISOP1N2OO + NO = MVK + HCHO + NO2 + NO2 : ALK(2.7E-12,350.,10.481,9.,1.,0.);
ISOP1N2OO + NO = ISOP1N2N : NIT(2.7E-12,350.,10.481,9.,1.,0.);
ISOP1N4OO + NO = ISOP1N4O + NO2 : ALK(2.7E-12,350.,1.412,9.,1.,0.);
ISOP1N4OO + NO = ISOP1N4N : NIT(2.7E-12,350.,1.412,9.,1.,0.);
ISOP3OO4N + NO = MACR + HCHO + NO2 + NO2 : ALK(2.7E-12,350.,13.202,9.,1.,0.);
ISOP3OO4N + NO = ISOP3N4N : NIT(2.7E-12,350.,13.202,9.,1.,0.);
ISOP1OO4N + NO = ISOP1O4N + NO2 : ALK(2.7E-12,350.,1.412,9.,1.,0.);
ISOP1OO4N + NO = ISOP1N4N : NIT(2.7E-12,350.,1.412,9.,1.,0.);
ISOP1N2OO + NO3 = MVK + HCHO + NO2 + NO2 : 2.3E-12;
ISOP1N4OO + NO3 = ISOP1N4O + NO2 : 2.3E-12;
ISOP3OO4N + NO3 = MACR + HCHO + NO2 + NO2 : 2.3E-12;
ISOP1OO4N + NO3 = ISOP1O4N + NO2 : 2.3E-12;
ISOP1N4O + O2 = ISOP1N4CO + HO2 : 2.5E-14*EXP(-300./TEMP);
ISOP1O4N + O2 = ISOP1CO4N + HO2 : 2.5E-14*EXP(-300./TEMP);
ISOP1N4O = ISOP1N253OO4OH : 1.0E20*EXP(-10000./TEMP);
ISOP1N253OO4OH + NO3 = ISOP1N253O4OH + NO2 : 2.3E-12;
ISOP1N253OO4OH + NO = ISOP1N253O4OH + NO2 : ALK(2.7E-12,350.,2.111,10.,1.,0.);
ISOP1N253OO4OH + NO = ISOP1N253N4OH + NO2 : NIT(2.7E-12,350.,2.111,10.,1.,0.);
ISOP1N253O4OH + O2 = MACR4N + HCHO + HO2 : 2.5E-14*EXP(-300./TEMP);
ISOP1N253OO4OH + HO2 = ISOP1N253OOH4OH : 0.65*2.54E-13*EXP(1300./TEMP);
ISOP1N253OO4OH + HO2 = ISOP1N253O4OH + OH : 0.35*2.54E-13*EXP(1300./TEMP);
ISOP1N253OO4OH + ISOP1N4OO = ISOP1N253CO4OH + ISOP1N4OHc : 3.94E-12*0.77*0.5*0.5;
ISOP1N253OO4OH + ISOP1N4OO = ISOP1N253CO4OH + ISOP1N4OHt : 3.94E-12*0.77*0.5*0.5;
ISOP1N253OO4OH + ISOP1N4OO = ISOP1N253OH4OH + ISOP1N4CO : 3.94E-12*0.77*0.5;
ISOP1N253OO4OH + ISOP1N4OO = ISOP1N253O4OH + ISOP1N4O : 3.94E-12*0.195;
ISOP1N253OO4OH + ISOP1N4OO = ISOP1N253OO4OHISOP1N4 : 3.94E-12*0.035;
ISOP1N253OO4OH + ISOP1N2OO = MVK + HCHO + NO2 + ISOP1N253O4OH : 3.08E-12*0.58;
ISOP1N253OO4OH + ISOP1N2OO = ISOP1N2OH + ISOP1N253CO4OH : 3.08E-12*0.77*0.5;

ISOP1N253OO4OH + ISOP1N2OO = ISOP1N253OO4OHISOP1N2 : 3.08E-12*0.035;
ISOP1N253OO4OH + CH3OO = ISOP1N253OH4OH + HCHO : 1.4E-12*0.71*0.5;
ISOP1N253OO4OH + CH3OO = ISOP1N253CO4OH + CH3OH : 1.4E-12*0.71*0.5;
ISOP1N253OO4OH + CH3OO = ISOP1N253O4OH + HCHO + HO2 : 1.4E-12*0.29;
ISOP1N2OOH + OH = ISOP1N2OOH3R4OH : 8.38E-12*EXP(390./TEMP)*0.75;
ISOP1N2OOH3R4OH + O2 = ISOP1N2OOH3OO4OH : 1.0E-14;
ISOP1N2OOH3R4OH = ISOP1N23O4OH + OH : 9.2E4;
ISOP1N2OOH + OH = ISOP1N2OOH3OH4OO : 8.38E-12*EXP(390./TEMP)*0.25;
ISOP1N4OOH + OH = ISOP1N2OH3R4OOH : 2.24E-11*EXP(390./TEMP)*0.3;
ISOP1N2OH3R4OOH + O2 = ISOP1N2OH3OO4OOH : 1.0E-14;
ISOP1N2OH3R4OOH = ISOP1N2OH34O + OH : 9.61E4;
ISOP1N4OOH + OH = ISOP1N2R3OH4OOH : 2.24E-11*EXP(390./TEMP)*0.7;
ISOP1N2R3OH4OOH + O2 = ISOP1N2OO3OH4OOH : 1.0E-14;
ISOP1N2R3OH4OOH = ISOP12O3OH4OOH + NO2 : 7.73E3;
ISOP3OOH4N + OH = ISOP1OH2R3OOH4N : 1.17E-11*EXP(390./TEMP)*0.9;
ISOP1OH2R3OOH4N + O2 = ISOP1OH2OO3OOH4N : 1.0E-14;
ISOP1OH2R3OOH4N = ISOP1OH23O4N + OH : 9.2E4;
ISOP3OOH4N + OH = ISOP1OO2OH3OOH4N : 1.17E-11*EXP(390./TEMP)*0.1;
ISOP1OOH4N + OH = ISOP1OOH2R3OH4N : 3.07E-11*EXP(390./TEMP)*0.7;
ISOP1OOH2R3OH4N + O2 = ISOP1OOH2OO3OH4N : 1.0E-14;
ISOP1OOH2R3OH4N = ISOP12O3OH4N + OH : 9.61E4;
ISOP1OOH4N + OH = ISOP1OOH2OH3R4N : 3.07E-11*EXP(390./TEMP)*0.3;
ISOP1OOH2OH3R4N + O2 = ISOP1OOH2OH3OO4N : 1.0E-14;
ISOP1OOH2OH3R4N = ISOP1OOH2OH34O + NO2 : 7.73E3;
ISOP1N2OOH + OH = ISOP1N2OO + H2O : 3.4E-12*EXP(200./TEMP);
ISOP1N4OOH + OH = ISOP1N4OO + H2O : 3.4E-12*EXP(200./TEMP);
ISOP3OOH4N + OH = ISOP3OO4N + H2O : 3.4E-12*EXP(200./TEMP);
ISOP1OOH4N + OH = ISOP1OO4N + H2O : 3.4E-12*EXP(200./TEMP);
ISOP1N4OOH + OH = OH + ISOP1N4CO : 7.5E-12*EXP(20./TEMP);
ISOP1OOH4N + OH = OH + ISOP1CO4N : 7.5E-12*EXP(20./TEMP);
ISOP3OOH4N + OH = OH + ISOP3CO4N : 7.5E-12*EXP(20./TEMP);
ISOP1N2OOH3OO4OH + NO = ISOP1N2OOH3N4OH : NIT(2.7E-12,350.,4.886,12.,1.,0.);
ISOP1N2OOH3OO4OH + NO = NO2 + PROPNN + GLYC + OH : ALK(2.1375E-12,350.,4.886,12.,1.,0.);
ISOP1N2OOH3OO4OH + NO = NO2 + HO2 + HCHO + MACR2OOH3N :
ALK(0.5625E-12,350.,4.886,12.,1.,0.);
ISOP1N2OOH3OO4OH + HO2 = OH + HO2 + HCHO + MACR2OOH3N :
0.15*2.64E-13*EXP(1300./TEMP);
ISOP1N2OOH3OO4OH + HO2 = OH + PROPNN + GLYC + OH : 0.6*2.64E-13*EXP(1300./TEMP);
ISOP1N2OOH3OO4OH + HO2 = ISOP1N2OOH3OOH4OH : 0.25*2.64E-13*EXP(1300./TEMP);
ISOP1N2OOH3OH4OO + NO = ISOP1N2OOH3OH4N : NIT(2.7E-12,350.,1.783,12.,1.,0.);

ISOP1N2OOH3OH4OO + NO = NO2 + HO2 + HCHO + MACR2OOH3N :
ALK(2.7E-12,350.,1.783,12.,1.,0.);
ISOP1N2OOH3OH4OO + HO2 = ISOP1N2OOH3OH4OOH : 0.8*2.64E-13*EXP(1300./TEMP);
ISOP1N2OOH3OH4OO + HO2 = OH + HO2 + HCHO + MACR2OOH3N :
0.2*2.64E-13*EXP(1300./TEMP);
ISOP1N2OH3OO4OOH + NO = ISOP1N2OH3N4OOH : NIT(2.7E-12,350.,4.886,12.,1.,0.);
ISOP1N2OH3OO4OOH + NO = NO2 + MACR2OH3N + HCHO + OH :
ALK(0.5625E-12,350.,4.886,12.,1.,0.);
ISOP1N2OH3OO4OOH + NO = NO2 + PROPNN + HPETHNL + HO2 :
ALK(2.1375E-12,350.,4.886,12.,1.,0.);
ISOP1N2OH3OO4OOH + HO2 = OH + PROPNN + HPETHNL + HO2 :
0.60*2.64E-13*EXP(1300./TEMP);
ISOP1N2OH3OO4OOH + HO2 = OH + MACR2OH3N + HCHO + OH : 0.15*2.64E-13*EXP(1300./TEMP);
ISOP1N2OH3OO4OOH + HO2 = ISOP1N2OH3OOH4OOH : 0.25*2.64E-13*EXP(1300./TEMP);
ISOP1N2OO3OH4OOH + NO = ISOP1N2N3OH4OOH : NIT(2.7E-12,350.,12.974,12.,1.,0.);
ISOP1N2OO3OH4OOH + NO = NO2 + PROPNN + HPETHNL + HO2 :
ALK(2.7E-12,350.,12.974,12.,1.,0.);
ISOP1N2OO3OH4OOH + HO2 = OH + PROPNN + HPETHNL + HO2 :
0.85*2.64E-13*EXP(1300./TEMP);
ISOP1N2OO3OH4OOH + HO2 = ISOP1N2OOH3OH4OOH : 0.15*2.64E-13*EXP(1300./TEMP);
ISOP1OH2OO3OOH4N + NO = ISOP1OH2N3OOH4N : NIT(2.7E-12,350.,3.818,12.,1.,0.);
ISOP1OH2OO3OOH4N + NO = NO2 + HO2 + HCHO + MVK3OOH4N :
ALK(0.5625E-12,350.,3.818,12.,1.,0.);
ISOP1OH2OO3OOH4N + NO = NO2 + HAC + ETHLN + OH : ALK(2.1375E-12,350.,3.818,12.,1.,0.);
ISOP1OH2OO3OOH4N + HO2 = OH + HAC + ETHLN + OH : 0.68*2.64E-13*EXP(1300./TEMP);
ISOP1OH2OO3OOH4N + HO2 = OH + HO2 + HCHO + MVK3OOH4N :
0.17*2.64E-13*EXP(1300./TEMP);
ISOP1OH2OO3OOH4N + HO2 = ISOP1OH2OOH3OOH4N : 0.15*2.64E-13*EXP(1300./TEMP);
ISOP1OO2OH3OOH4N + NO = ISOP1N2OH3OOH4N : NIT(2.7E-12,350.,1.783,12.,1.,0.);
ISOP1OO2OH3OOH4N + NO = HO2 + NO2 + HCHO + MVK3OOH4N :
ALK(2.7E-12,350.,1.783,12.,1.,0.);
ISOP1OO2OH3OOH4N + HO2 = ISOP1OOH2OH3OOH4N : 0.8*2.64E-13*EXP(1300./TEMP);
ISOP1OO2OH3OOH4N + HO2 = OH + HO2 + HCHO + MVK3OOH4N : 0.2*2.64E-13*EXP(1300./TEMP);
ISOP1OOH2OO3OH4N + NO = ISOP1OOH2N3OH4N : NIT(2.7E-12,350.,3.818,12.,1.,0.);
ISOP1OOH2OO3OH4N + NO = NO2 + HPAC + ETHLN + HO2 : ALK(2.1375E-12,350.,3.818,12.,1.,0.);
ISOP1OOH2OO3OH4N + NO = NO2 + MVK3OH4N + HCHO + OH :
ALK(0.5625E-12,350.,3.818,12.,1.,0.);
ISOP1OOH2OO3OH4N + HO2 = OH + MVK3OH4N + HCHO + OH : 0.17*2.64E-13*EXP(1300./TEMP);
ISOP1OOH2OO3OH4N + HO2 = OH + HPAC + ETHLN + HO2 : 0.68*2.64E-13*EXP(1300./TEMP);
ISOP1OOH2OO3OH4N + HO2 = ISOP1OOH2OOH3OH4N : 0.15*2.64E-13*EXP(1300./TEMP);
ISOP1OOH2OH3OO4N + NO = ISOP1OOH2OH3N4N : NIT(2.7E-12,350.,16.331,12.,1.,0.);

ISOP1OOH2OH3OO4N + NO = NO2 + HPAC + ETHLN + HO2 : ALK(2.7E-12,350.,16.331,12.,1.,0.);
ISOP1OOH2OH3OO4N + HO2 = OH + HPAC + ETHLN + HO2 : 0.75*2.64E-13*EXP(1300./TEMP);
ISOP1OOH2OH3OO4N + HO2 = ISOP1OOH2OH3OOH4N : 0.25*2.64E-13*EXP(1300./TEMP);
ISOP1N2OOH3OO4OH = ISOP1N2OO3OOH4OH : 4.0E6;
ISOP1N2OOH3OH4OO = ISOP1N2OO3OH4OOH : 4.0E6;
ISOP1N2OH3OO4OOH = ISOP1N2OH3OOH4OO : 2.0E6;
ISOP1N2OO3OH4OOH = ISOP1N2OOH3OH4OO : 2.0E6;
ISOP1OH2OO3OOH4N = ISOP1OH2OOH3OO4N : 3.0E6;
ISOP1OO2OH3OOH4N = ISOP1OOH2OH3OO4N : 3.0E6;
ISOP1OOH2OO3OH4N = ISOP1OO2OOH3OH4N : 2.0E6;
ISOP1OOH2OH3OO4N = ISOP1OO2OH3OOH4N : 2.0E6;
ISOP1N2OO3OOH4OH = ISOP1N2OOH3OO4OH : 3.0E6;
ISOP1N2OH3OOH4OO = ISOP1N2OH3OO4OOH : 3.0E6;
ISOP1OH2OOH3OO4N = ISOP1OH2OO3OOH4N : 4.0E6;
ISOP1OO2OOH3OH4N = ISOP1OOH2OO3OH4N : 4.0E6;
ISOP1N2OO3OOH4OH + HO2 = MVK3OOH4OH + NO2 + HCHO + OH :
0.2*2.64E-13*EXP(1300./TEMP);
ISOP1N2OO3OOH4OH + HO2 = OH + OH + GLYC + PROPNN : 0.8*2.64E-13*EXP(1300./TEMP);
ISOP1N2OO3OOH4OH + NO = ISOP1N2N3OOH4OH : NIT(2.7E-12,350.,21.808,12.,1.,0.);
ISOP1N2OO3OOH4OH + NO = MVK3OOH4OH + NO2 + NO2 + HCHO :
ALK(0.5625E-12,350.,21.808,12.,1.,0.);
ISOP1N2OO3OOH4OH + NO = NO2 + OH + GLYC + PROPNN : ALK(2.1375E-12,350.,21.808,12.,1.,0.);
ISOP1N2OH3OOH4OO + HO2 = ISOP1N2OH3OOH4OOH : 0.8*2.64E-13*EXP(1300./TEMP);
ISOP1N2OH3OOH4OO + HO2 = OH + OH + HCHO + MACR2OH3N : 0.2*2.64E-13*EXP(1300./TEMP);
ISOP1N2OH3OOH4OO + NO = ISOP1N2OH3OOH4N : NIT(2.7E-12,350.,3.256,12.,1.,0.);
ISOP1N2OH3OOH4OO + NO = MACR2OH3N + OH + NO2 + HCHO : ALK(2.7E-12,350.,3.256,12.,1.,0.);
ISOP1OH2OOH3OO4N + HO2 = ISOP1OH2OOH3OOH4N : 0.25*2.64E-13*EXP(1300./TEMP);
ISOP1OH2OOH3OO4N + HO2 = MACR2OOH3OH + OH + NO2 + HCHO :
0.15*2.64E-13*EXP(1300./TEMP);
ISOP1OH2OOH3OO4N + HO2 = HAC + ETHLN + OH + OH : 0.6*2.64E-13*EXP(1300./TEMP);
ISOP1OH2OOH3OO4N + NO = ISOP1OH2OOH3N4N : NIT(2.7E-12,350.,27.374,12.,1.,0.);
ISOP1OH2OOH3OO4N + NO = MACR2OOH3OH + NO2 + NO2 + HCHO :
ALK(0.5625E-12,350.,27.374,12.,1.,0.);
ISOP1OH2OOH3OO4N + NO = HAC + ETHLN + OH + NO2: ALK(2.1375E-12,350.,27.374,12.,1.,0.);
ISOP1OO2OOH3OH4N + HO2 = ISOP1OOH2OOH3OH4N : 0.8*2.64E-13*EXP(1300./TEMP);
ISOP1OO2OOH3OH4N + HO2 = OH + OH + HCHO + MVK3OH4N : 0.2*2.64E-13*EXP(1300./TEMP);
ISOP1OO2OOH3OH4N + NO = ISOP1N2OOH3OH4N : NIT(2.7E-12,350.,3.256,12.,1.,0.);
ISOP1OO2OOH3OH4N + NO = MVK3OH4N + OH + NO2 + HCHO : ALK(2.7E-12,350.,3.256,12.,1.,0.);
ISOP1N2OO3OH4OOH = ISOP1N2OOH3OH4CO + OH : 9.375E12*EXP(-10000./TEMP);
ISOP1OOH2OH3OO4N = ISOP1CO2OH3OOH4N + OH : 9.375E12*EXP(-10000./TEMP);
ISOP1OO2OOH3OH4N = ISOP1OOH23O3OH4N + OH : 3.75E13*EXP(-10000./TEMP);

ISOP1N2OO3OOH4OH = ISOP1N2OOH34O4OH + OH : 1.875E13*EXP(-10000./TEMP);
ISOP1OO2OH3OOH4N = ISOP1OOH2OH3CO4N + OH : 1.875E13*EXP(-10000./TEMP);
ISOP1OH2OOH3OO4N = ISOP1OH12O3OOH4N + OH : 1.875E13*EXP(-10000./TEMP);
ISOP1N23O4OH + OH = ISOP1N23O4OH4R : 4.78E-11*EXP(-400./TEMP);
ISOP1N23O4OH4R + O2 = ISOP1N23O4CO + HO2 : 1.0E-14;
ISOP1N23O4OH4R = HCHO + NO2 + MVKENOL : 0.5*2.07E5;
ISOP1N23O4OH4R = ISOP1N2OH3OO4CO : 0.5*2.07E5;
ISOP1OH23O4N + OH = ISOP1OH1R23O4N : 4.78E-11*EXP(-400./TEMP);
ISOP1OH1R23O4N + O2 = ISOP1CO23O4N + HO2 : 1.0E-14;
ISOP1OH1R23O4N = HCHO + NO2 + MACRENOL : 0.5*2.07E5;
ISOP1OH1R23O4N = ISOP1CO2OO3OH4N : 0.5*2.07E5;
ISOP1N2OH34O + OH = ISOP1N2OH3OO4CO : 0.5*3.22E-11*EXP(-400./TEMP);
ISOP1N2OH34O + OH = ISOP1N2OH3CO4OO : 0.5*3.22E-11*EXP(-400./TEMP);
ISOP12O3OH4N + OH = ISOP12O3OH3R4N : 3.22E-11*EXP(-400./TEMP);
ISOP12O3OH3R4N + O2 = ISOP12O3CO4N + HO2 : 1.0E-14;
ISOP12O3OH3R4N = ISOP1OH2OO3CO4N : 2.07E5;
ISOP1N2OH3CO4OO + NO = ISOP1N2OH3CO4N : NIT(2.7E-12,350.,15.73,11.,1.,0.);
ISOP1N2OH3CO4OO + NO = PROPNN + CO + HO2 + HCHO + NO2 :
ALK(2.7E-12,350.,15.73,11.,1.,0.);
ISOP1N2OH3CO4OO + HO2 = PROPNN + CO + HO2 + HCHO + OH : 0.2*2.6E-13*EXP(1300./TEMP);
ISOP1N2OH3CO4OO + HO2 = ISOP1N2OH3CO4OOH : 0.8*2.6E-13*EXP(1300./TEMP);
ISOP3CO4N + OH = ISOP1OH2OO3CO4N : 2.7E-12*EXP(390./TEMP);
ISOP1OH2OO3CO4N + HO2 = ISOP1OH2OOH3CO4N : 2.6E-13*EXP(1300./TEMP)*0.15;
ISOP1OH2OO3CO4N + HO2 = HAC + OH + NPA : 2.6E-13*EXP(1300./TEMP)*0.85*0.25;
ISOP1OH2OO3CO4N + HO2 = MVK3CO4N + HO2 + OH + HCHO :
2.6E-13*EXP(1300./TEMP)*0.85*0.75;
ISOP1OH2OO3CO4N + NO = MVK3CO4N + NO2 + HO2 + HCHO :
ALK(2.025E-12,350.,18.181,11.,1.,0.);
ISOP1OH2OO3CO4N + NO = ISOP1OH2N3CO4N : NIT(2.7E-12,350.,18.181,11.,1.,0.);
ISOP1OH2OO3CO4N + NO = HAC + NO2 + NPA : ALK(0.675E-12,350.,18.181,11.,1.,0.);
ISOP1N2OO3CO4OH + NO = ISOP1N2N3CO4OH : NIT(2.7E-12,350.,96.601,11.,1.,0.);
ISOP1N2OO3CO4OH + NO = NO2 + PROPNN + CH3CO3 : ALK(2.7E-12,350.,96.601,11.,1.,0.);
ISOP1N2OO3CO4OH + HO2 = OH + PROPNN + CH3CO3 : 2.6E-13*EXP(1300./TEMP);
ISOP1N4CO + OH = ISOP1N4R4CO : 3.3E-12*EXP(470./TEMP);
ISOP1N4R4CO + O2 = ISOP1N4CO4OO : 1.0E-14;
ISOP1N4CO4OO + NO2 = ISOP1N4PAN : TROE(2.591E-28,0.,-6.87,1.125E-11,0.,-1.105,0.3);
ISOP1N4CO4OO + NO = NO2 + CO2 + C4NVP2 : 2.7E-12*EXP(350./TEMP);
ISOP1N4CO4OO + HO2 = ISOP1N4CO4OOH : 3.14E-12*EXP(580./TEMP)*0.37;
ISOP1N4CO4OO + HO2 = O3 + ISOP1N4CO4OH : 3.14E-12*EXP(580./TEMP)*0.13;
ISOP1N4CO4OO + HO2 = OH + CO2 + C4NVP2 : 3.14E-12*EXP(580./TEMP)*0.5;
C4NVP2 + NO = NO2 + MACR2OO3N : 2.7E-12*EXP(350./TEMP);

C4NVP2 + HO2 = OH + MACR2OO3N : 2.38E-13*EXP(1300./TEMP);
C4NVP2 + NO2 = MACR2N3N : 9.0E-12;
MACR2OO3N = CO + OH + PROPNN : 1.0E7*EXP(-5000./TEMP);
MACR2OO3N + NO = NO2 + HO2 + CO + PROPNN : 2.7E-12*EXP(350./TEMP);
MACR2OO3N + HO2 = OH + HO2 + CO + PROPNN : 2.47E-13*EXP(1300./TEMP);
ISOP1N4PAN = ISOP1N4CO4OO + NO2 : 1.58E16*EXP(-13500./TEMP);
ISOP1CO4N + OH = ISOP1CO1R4N : 3.3E-12*EXP(470./TEMP);
ISOP1CO1R4N + O2 = ISOP1CO1OO4N : 1.0E-14;
ISOP1CO1OO4N + NO2 = ISOP1PAN4N : TROE(2.591E-28,0.,-6.87,1.125E-11,0.,-1.105,0.3);
ISOP1CO1OO4N + NO = NO2 + CO2 + C4NVP1 : 2.7E-12*EXP(350./TEMP);
ISOP1CO1OO4N + HO2 = ISOP1CO1OOH4N : 3.14E-12*EXP(580./TEMP)*0.37;
ISOP1CO1OO4N + HO2 = O3 + ISOP1CO1OH4N : 3.14E-12*EXP(580./TEMP)*0.13;
ISOP1CO1OO4N + HO2 = OH + CO2 + C4NVP1 : 3.14E-12*EXP(580./TEMP)*0.5;
C4NVP1 + NO = NO2 + MVK3OO4N : 2.7E-12*EXP(350./TEMP);
C4NVP1 + HO2 = OH + MVK3OO4N : 2.38E-13*EXP(1300./TEMP);
C4NVP1 + NO2 = MVK3N4N : 9.0E-12;
MVK3OO4N + NO = NO2 + ETHLN + CH3CO3 : 2.7E-12*EXP(350./TEMP);
MVK3OO4N + HO2 = OH + ETHLN + CH3CO3 : 2.47E-13*EXP(1300./TEMP);
ISOP1PAN4N = ISOP1CO1OO4N + NO2 : 1.58E16*EXP(-13500./TEMP);
ISOP1N4CO + OH = ISOP1N2R3OH4CO : 4.86E-12*EXP(390./TEMP)*0.7;
ISOP1N2R3OH4CO + O2 = ISOP1N2OO3OH4CO : 1.0E-14;
ISOP1N2R3OH4CO = ISOP12O3OH4CO + NO2 : 7.73E3;
ISOP1N4CO + OH = ISOP1N2OH3OO4CO : 4.86E-12*EXP(390./TEMP)*0.3;
ISOP1CO4N + OH = ISOP1CO2OH3R4N : 6.75E-12*EXP(390./TEMP)*0.3;
ISOP1CO2OH3R4N + O2 = ISOP1CO2OH3OO4N : 1.0E-14;
ISOP1CO2OH3R4N = ISOP1CO2OH34O + NO2 : 7.73E3;
ISOP1CO4N + OH = ISOP1CO2OO3OH4N : 6.75E-12*EXP(390./TEMP)*0.7;
ISOP1N2OO3OH4CO = MACR2OOH3N + CO + HO2 : 4.0E8*EXP(-5000./TEMP);
ISOP1CO2OH3OO4N = MVK3OOH4N + CO + HO2 : 4.0E8*EXP(-5000./TEMP);
ISOP1N2OH3OO4CO = MACR2OH3N + CO + OH : 1.0E7*EXP(-5000./TEMP);
ISOP1N2OH3OO4CO + HO2 = ISOP1N2OH3OOH4CO : 2.6E-13*EXP(1300./TEMP)*0.25;
ISOP1N2OH3OO4CO + HO2 = MACR2OH3N + OH + HO2 + CO :
2.6E-13*EXP(1300./TEMP)*0.75*0.75;
ISOP1N2OH3OO4CO + HO2 = OH + HO2 + PROPNN + GLYX : 2.6E-13*EXP(1300./TEMP)*0.75*0.25;
ISOP1N2OH3OO4CO + NO = ISOP1N2OH3N4CO : NIT(2.7E-12,350.,22.837,11.,1.,0.);
ISOP1N2OH3OO4CO + NO = MACR2OH3N + NO2 + HO2 + CO : ALK(2.025E-12,350.,22.837,11.,1.,0.);
ISOP1N2OH3OO4CO + NO = NO2 + HO2 + PROPNN + GLYX : ALK(0.675E-12,350.,22.837,11.,1.,0.);
ISOP1CO2OO3OH4N = MVK3OH4N + CO + OH : 1.0E7*EXP(-5000./TEMP);
ISOP1CO2OO3OH4N + HO2 = MVK3OH4N + OH + HO2 + CO : 2.6E-13*EXP(1300./TEMP)*0.85*0.75;
ISOP1CO2OO3OH4N + HO2 = OH + HO2 + MGLY + ETHLN : 2.6E-13*EXP(1300./TEMP)*0.85*0.25;
ISOP1CO2OO3OH4N + HO2 = ISOP1CO2OOH3OH4N : 2.6E-13*EXP(1300./TEMP)*0.15;

ISOP1CO2OO3OH4N + NO = MVK3OH4N + NO2 + HO2 + CO : ALK(2.025E-12,350.,18.181,11.,1.,0.);
ISOP1CO2OO3OH4N + NO = NO2 + HO2 + MGLY + ETHLN: ALK(0.675E-12,350.,18.181,11.,1.,0.);
ISOP1CO2OO3OH4N + NO = ISOP1CO2N3OH4N : NIT(2.7E-12,350.,18.181,11.,1.,0.);
ISOP1CO4N = MVKENOL + OH + NO2 + CO : 0.552*0.58*SUN*4.0E-4;
ISOP1CO4N = C4HVP1 + NO2 + CO : 0.224*0.58*SUN*4.0E-4;
ISOP1CO4N = ISOP1CO4CO + HO2 + NO2 : 0.112*0.58*SUN*4.0E-4;
ISOP1CO4N = ISOP1CO2OO3OOH4CO + NO2 : 0.112*0.58*SUN*4.0E-4;
ISOP1N4CO = MACRENOL + NO2 + OH + CO : 0.455*0.55*SUN*4.0E-4;
ISOP1N4CO = C4HVP2 + NO2 + CO : 0.182*0.55*SUN*4.0E-4;
ISOP1N4CO = ISOP1CO4CO + HO2 + NO2 : 0.182*0.55*SUN*4.0E-4;
ISOP1N4CO = ISOP1CO2OOH3OO4CO + NO2 : 0.182*0.55*SUN*4.0E-4;
ISOP3CO4N = NO2 + HCHO + MACR1OO : 0.56*SUN*4.0E-4;
ISOP1N2OH + OH = ISOP1N2OH3OO4OH : 8.38E-12*EXP(390./TEMP)*0.75;
ISOP1N2OH + OH = ISOP1N2OH3OH4OO : 8.38E-12*EXP(390./TEMP)*0.25;
ISOP1N4OHc + OH = ISOP1N2R3OH4OH : 2.24E-11*EXP(390./TEMP)*0.7;
ISOP1N4OHt + OH = ISOP1N2R3OH4OH : 2.24E-11*EXP(390./TEMP)*0.7;
ISOP1N2R3OH4OH + O2 = ISOP1N2OO3OH4OH : 1.0E-14;
ISOP1N2R3OH4OH = ISOP12O3OH4OH : 7.73E3;
ISOP1N4OHc + OH = ISOP1N2OH3OO4OH : 2.24E-11*EXP(390./TEMP)*0.3;
ISOP1N4OHt + OH = ISOP1N2OH3OO4OH : 2.24E-11*EXP(390./TEMP)*0.3;
ISOP3OH4N + OH = ISOP1OH2OO3OH4N : 1.17E-11*EXP(390./TEMP)*0.9;
ISOP3OH4N + OH = ISOP1OO2OH3OH4N : 1.17E-11*EXP(390./TEMP)*0.1;
ISOP1OH4Nc + OH = ISOP1OH2OH3R4N : 3.07E-11*EXP(390./TEMP)*0.3;
ISOP1OH4Nt + OH = ISOP1OH2OH3R4N : 3.07E-11*EXP(390./TEMP)*0.3;
ISOP1OH2OH3R4N + O2 = ISOP1OH2OH3OO4N : 1.0E-14;
ISOP1OH2OH3R4N = ISOP1OH2OH34O : 7.73E3;
ISOP1OH4Nc + OH = ISOP1OH2OO3OH4N : 3.07E-11*EXP(390./TEMP)*0.7;
ISOP1OH4Nt + OH = ISOP1OH2OO3OH4N : 3.07E-11*EXP(390./TEMP)*0.7;
ISOP1OH2OH3OO4N = ISOP1CO2OH3OOH4N + HO2 : 1.875E13*EXP(-10000./TEMP);
ISOP1N2OO3OH4OH = ISOP1N2OOH3OH4CO + HO2 : 1.875E13*EXP(-10000./TEMP);
ISOP1N2OO3OH4OH + HO2 = ISOP1N2OOH3OH4OH : 2.6E-13*EXP(1300./TEMP)*0.35;
ISOP1N2OO3OH4OH + HO2 = OH + HO2 + PROPNN + GLYC : 2.6E-13*EXP(1300./TEMP)*0.65;
ISOP1N2OH3OO4OH + HO2 = ISOP1N2OH3OOH4OH : 2.6E-13*EXP(1300./TEMP)*0.55;
ISOP1N2OH3OO4OH + HO2 = MACR2OH3N + HCHO + HO2 + OH : 2.6E-13*EXP(1300./TEMP)*0.09;
ISOP1N2OH3OO4OH + HO2 = OH + HO2 + PROPNN + GLYC : 2.6E-13*EXP(1300./TEMP)*0.36;
ISOP1OH2OH3OO4N + HO2 = ISOP1OH2OH3OOH4N : 2.6E-13*EXP(1300./TEMP)*0.35;
ISOP1OH2OH3OO4N + HO2 = OH + HO2 + HAC + ETHLN : 2.6E-13*EXP(1300./TEMP)*0.65;
ISOP1OH2OO3OH4N + HO2 = ISOP1OH2OOH3OH4N : 2.6E-13*EXP(1300./TEMP)*0.55;
ISOP1OH2OO3OH4N + HO2 = MVK3OH4N + HCHO + HO2 + OH : 2.6E-13*EXP(1300./TEMP)*0.09;
ISOP1OH2OO3OH4N + HO2 = OH + HO2 + HAC + ETHLN : 2.6E-13*EXP(1300./TEMP)*0.36;
ISOP1N2OO3OH4OH + NO = ISOP1N2N3OH4OH : NIT(2.7E-12,350.,10.339,11.,1.,0.);

ISOP1N2OO3OH4OH + NO = NO2 + HO2 + PROPNN + GLYC : ALK(2.7E-12,350.,10.339,11.,1.,0.);
ISOP1N2OH3OO4OH + NO = ISOP1N2OH3N4OH : NIT(2.7E-12,350.,2.143,11.,1.,0.);
ISOP1N2OH3OO4OH + NO = MACR2OH3N + HCHO + HO2 + NO2 :
ALK(0.5625E-12,350.,2.143,11.,1.,0.);
ISOP1N2OH3OO4OH + NO = NO2 + HO2 + PROPNN + GLYC : ALK(2.1375E-12,350.,2.143,11.,1.,0.);
ISOP1OH2OH3OO4N + NO = ISOP1OH2OH3N4N : NIT(2.7E-12,350.,13.035,11.,1.,0.);
ISOP1OH2OH3OO4N + NO = NO2 + HO2 + HAC + ETHLN : ALK(2.7E-12,350.,13.035,11.,1.,0.);
ISOP1OH2OO3OH4N + NO = ISOP1OH2N3OH4N : NIT(2.7E-12,350.,1.626,11.,1.,0.);
ISOP1OH2OO3OH4N + NO = MVK3OH4N + HCHO + HO2 + NO2 :
ALK(0.5625E-12,350.,1.626,11.,1.,0.);
ISOP1OH2OO3OH4N + NO = NO2 + HO2 + HAC + ETHLN : ALK(2.1375E-12,350.,1.626,11.,1.,0.);
ISOP1OO2OH3OH4N = ISOP1OOH2OH3CO4N + HO2 : 3.75E13*EXP(-10000./TEMP);
ISOP1N2OH3OH4OO + HO2 = ISOP1N2OH3OH4OOH : 0.85*2.6E-13*EXP(1300./TEMP);
ISOP1N2OH3OH4OO + HO2 = OH + HO2 + HCHO + MACR2OH3N : 0.15*2.6E-13*EXP(1300./TEMP);
ISOP1OO2OH3OH4N + HO2 = ISOP1OOH2OH3OH4N : 0.85*2.6E-13*EXP(1300./TEMP);
ISOP1OO2OH3OH4N + HO2 = OH + HO2 + HCHO + MVK3OH4N : 0.15*2.6E-13*EXP(1300./TEMP);
ISOP1N2OH3OH4OO + NO = ISOP1N2OH3OH4N : NIT(2.7E-12,350.,1.353,11.,1.,0.);
ISOP1N2OH3OH4OO + NO = HO2 + NO2 + HCHO + MACR2OH3N : ALK(2.7E-12,350.,1.353,11.,1.,0.);
ISOP1OO2OH3OH4N + NO = ISOP1N2OH3OH4N : NIT(2.7E-12,350.,1.353,11.,1.,0.);
ISOP1OO2OH3OH4N + NO = HO2 + NO2 + HCHO + MVK3OH4N : ALK(2.7E-12,350.,1.353,11.,1.,0.);
ISOP1N4OHc + OH = ISOP1N4R4OH : 7.5E-12*EXP(20./TEMP);
ISOP1N4OHt + OH = ISOP1N4R4OH : 7.5E-12*EXP(20./TEMP);
ISOP1N4R4OH + O2 = ISOP1N4CO + HO2 : 0.4*1.0E-14;
ISOP1N4R4OH + O2 = ISOP1N2OOH3OO4CO : 0.6*1.0E-14;
ISOP1N2OOH3OO4CO = ISOP1N2OO3OOH4CO : 4.E6;
ISOP1N2OO3OOH4CO = ISOP1N2OOH3OO4CO : 3.E6;
ISOP1N2OO3OOH4CO = OH + CO + MACR2OOH3N : 4.0E8*EXP(-5000./TEMP);
ISOP1N2OOH3OO4CO = OH + CO + MACR2OOH3N : 1.0E7*EXP(-5000./TEMP);
ISOP1OH4Nc + OH = ISOP1OH1R4N : 7.5E-12*EXP(20./TEMP);
ISOP1OH4Nt + OH = ISOP1OH1R4N : 7.5E-12*EXP(20./TEMP);
ISOP1OH1R4N + O2 = ISOP1CO4N + HO2 : 0.4*1.0E-14;
ISOP1OH1R4N + O2 = ISOP1CO2OO3OOH4N : 0.6*1.0E-14;
ISOP1CO2OO3OOH4N = ISOP1CO2OOH3OO4N : 3.E6;
ISOP1CO2OOH3OO4N = ISOP1CO2OO3OOH4N : 4.E6;
ISOP1CO2OO3OOH4N = OH + CO + MVK3OOH4N : 1.0E7*EXP(-5000./TEMP);
ISOP1CO2OOH3OO4N = OH + CO + MVK3OOH4N : 4.0E8*EXP(-5000./TEMP);
ISOP3OH4N + OH = ISOP3OH3R4N : 7.5E-12*EXP(20./TEMP);
ISOP3OH3R4N + O2 = ISOP3CO4N + HO2 : 0.6*1.0E-14;
ISOP3OH3R4N + O2 = ISOP1OOH2R3CO4N : 0.4*1.0E-14;
ISOP1OOH2R3CO4N = OH + ISOP12O3CO4N : 1.0E3;

*1.0OH + 1.0ISOP1OH2OH = 1.06OH + 0.355CO2 + 0.355MGLY + 0.452HO2 + 0.807CO + 0.452MACR2OOH3OH + 0.193ISOP1OH2OH3OO4CO : 4.64E-12*EXP(650./TEMP);*
*1.0OH + 1.0ISOP1OH4OH = 1.06OH + 0.355CO2 + 0.355MGLY + 0.452HO2 + 0.807CO + 0.452MACR2OOH3OH + 0.193ISOP1OH2OH3OO4CO : 4.64E-12*EXP(650./TEMP);*
*1.0OH + 1.0ISOP3OH4OH = 1.0ISOP1OH2OO3CO4OH : 2.7E-11*EXP(390./TEMP);*
*1.0HO2 + 1.0ISOP1OH2OO3CO4OH = 0.0219ISOP1OH2OOH3OOH4CO + 0.0219ISOP1CO2OOH3OOH4OH + 0.0219ISOP1OH2OOH3CO4OH + 0.0219ISOP1CO2OOH3OOH4OOH + 0.0219ISOP1OOH2OOH3OOH4CO + 0.0219ISOP1OH2OOH3OH4CO + 0.0219ISOP1OOH2OH3CO4OH + 0.0219ISOP1CO2OH3OOH4OH + 0.0219ISOP1OH3OH4CO + 0.0219ISOP1CO3OH4OH + 0.0219ISOP1OH2OH3OOH4CO + 0.0219ISOP1CO2OOH3OH4OH + 0.0219ISOP1CO1OH4OH + 0.0219ISOP1OH4CO4OH + 0.0219ISOP1CO1OOH4OH + 0.0219ISOP1OH4CO4OOH + 0.65OH + 0.13MVK3CO4OH + 0.65HCHO + 0.65HO2 + 0.52HAC + 0.52CO : 2.38E-13*EXP(1300./TEMP);*
1.0NO + 1.0ISOP1OH2OO3CO4OH = 0.5ISOP1OH2N3CO4OH + 0.5ISOP1OH2OH3N4CO : NIT(2.7E-12,350.,13.098,8.,1.,0.);
1.0NO + 1.0ISOP1OH2OO3CO4OH = 1.0NO2 + 0.8HAC + 0.8CO + 1.0HCHO + 1.0HO2 + 0.2MVK3CO4OH : ALK(2.7E-12,350.,13.098,8.,1.,0.);
*1.0ISOP1OH2OO3CO4OH = 1.0HO2 + 1.0CO + 1.0CO + 1.0HAC + 1.0OH : 1.875E13*EXP(-10000./TEMP);*
1.0ISOP1OH2OOH3OH4OO = 0.167ISOP1CO2OOH34O4OH + 0.167ISOP1OH12O3OOH4CO + 0.167ISOP1OOH2OH34O4OH + 0.167ISOP1OH12O3OOH4OH + 0.167ISOP1OH12O3OH4OOH + 0.167ISOP1OH2OOH34O4OH + 1.0OH : TUN(6.80E12,1.12E4,8.46E7);
1.0ISOP1OH2OO3OH4OOH = 0.167ISOP1CO2OOH34O4OH + 0.167ISOP1OH12O3OOH4CO + 0.167ISOP1OOH2OH34O4OH + 0.167ISOP1OH12O3OOH4OH + 0.167ISOP1OH12O3OH4OOH + 0.167ISOP1OH2OOH34O4OH + 1.0OH : TUN(6.80E12,1.12E4,8.46E7);
1.0NO + 1.0ISOP1OH2OOH3OH4OO = 0.17MACR2OOH3OH + 0.17HCHO + 0.83HPETHNL + 0.83HAC + 1.0NO2 + 1.0HO2 : ALK(2.7E-12,350.,2.1,9.,1.,0.);
1.0NO + 1.0ISOP1OH2OO3OH4OOH = 0.17MACR2OOH3OH + 0.17HCHO + 0.83HPETHNL + 0.83HAC + 1.0NO2 + 1.0HO2 : ALK(2.7E-12,350.,2.1,9.,1.,0.);
1.0NO + 1.0ISOP1OH2OOH3OH4OO = 0.1ISOP1OH2OOH3OH4N + 0.1ISOP1OH2N3OH4OOH + 0.1ISOP1N2OH3OOH4OH + 0.1ISOP1OOH2OH3N4OH + 0.1ISOP1OH2OOH3N4OH + 0.1ISOP1OH2N3OOH4OH + 0.1ISOP1N2OOH3OH4OH + 0.1ISOP1OH2OH3OOH4N + 0.1ISOP1N2OH3OH4OOH + 0.1ISOP1OOH2OH3OH4N : NIT(2.7E-12,350.,2.1,9.,1.,0.);
1.0NO + 1.0ISOP1OH2OO3OH4OOH = 0.1ISOP1OH2OOH3OH4N + 0.1ISOP1OH2N3OH4OOH + 0.1ISOP1N2OH3OOH4OH + 0.1ISOP1OOH2OH3N4OH + 0.1ISOP1OH2OOH3N4OH + 0.1ISOP1OH2N3OOH4OH + 0.1ISOP1N2OOH3OH4OH + 0.1ISOP1OH2OH3OOH4N + 0.1ISOP1N2OH3OH4OOH + 0.1ISOP1OOH2OH3OH4N : NIT(2.7E-12,350.,2.1,9.,1.,0.);
*1.0HO2 + 1.0ISOP1OH2OOH3OH4OO = 0.197ISOP1OH2OOH3OH4OOH + 0.197ISOP1OOH2OH3OOH4OH + 0.197ISOP1OH2OOH3OOH4OH + 0.032MACR2OOH3OH + 0.032HCHO + 0.378HPETHNL + 0.378HAC + 0.41OH + 0.41HO2 : 2.47E-13*EXP(1300./TEMP);*
*1.0HO2 + 1.0ISOP1OH2OO3OH4OOH = 0.197ISOP1OH2OOH3OH4OOH + 0.197ISOP1OOH2OH3OOH4OH + 0.197ISOP1OH2OOH3OOH4OH + 0.032MACR2OOH3OH + 0.032HCHO + 0.378HPETHNL + 0.378HAC + 0.41OH + 0.41HO2 : 2.47E-13*EXP(1300./TEMP);*
*1.0OH + 1.0MVK3OOH4CO4OOH = 1.0OH + 1.0MGLY + 1.0CO2 : 5E-12*EXP(470./TEMP);*
*1.0MVK3OOH4CO4OOH = 0.5GLYX + 1.5HO2 + 0.5CH3CO3 + 0.5CO + 0.5MGLY : 2.5E-4*SUN;*
*1.0OH + 1.0MACR2OOH3CO3OOH = 1.0CO2 + 1.0OH + 1.0CO + 1.0HO2 + 1.0CH3CO3 : 5.0E-12*EXP(470./TEMP);*

1.0CH3CO3 + 1.0ISOP3OO4N = 1.0HCHO + 1.0NO2 + 1.0CH3OO + 1.0CO2 + 0.903MVK + 0.097MACR : 1.92E-12 ;
1.0CH3CO3 + 1.0ISOP1N4OO = 1.0CH3OO + 1.0CO2 + 0.841ISOP1N4O + 0.159HO2 + 0.159ISOP1CO4N : 7.71E-12 ;
1.0CH3CO3 + 1.0ISOP1OO4N = 1.0CH3OO + 1.0CO2 + 0.841ISOP1N4O + 0.159HO2 + 0.159ISOP1CO4N : 7.71E-12 ;
*1.0O2 + 1.0ISOP1N4O = 1.0ISOP1N4CO + 1.0HO2 : 2.5E-14*EXP(-300./TEMP) ;*
*1.0ISOP1N4O = 1.0IDHNBOO : 1.0E20*EXP(-10000./TEMP) ;*
*1.0HO2 + 1.0ISOP1N2OH3OO4OH = 0.0418ISOP1OH2OOH3OH4N + 0.0418ISOP1OH2N3OH4OOH + 0.0418ISOP1N2OH3OOH4OH + 0.0418ISOP1OOH2OH3N4OH + 0.0418ISOP1OH2OOH3N4OH + 0.0418ISOP1OH2N3OOH4OH + 0.0418ISOP1N2OOH3OH4OH + 0.0418ISOP1OH2OH3OOH4N + 0.0418ISOP1N2OH3OH4OOH + 0.0418ISOP1OOH2OH3OH4N + 0.551PROPNN + 0.551GLYC + 0.0103MACR2OH3N + 0.0103MACR2OH3N + 0.0103MACR2OH3N + 0.031HCHO + 0.582HO2 + 0.582OH : 2.6E-13*EXP(1300./TEMP);*
*1.0HO2 + 1.0ISOP1N2OO3OH4OH = 0.0418ISOP1OH2OOH3OH4N + 0.0418ISOP1OH2N3OH4OOH + 0.0418ISOP1N2OH3OOH4OH + 0.0418ISOP1OOH2OH3N4OH + 0.0418ISOP1OH2OOH3N4OH + 0.0418ISOP1OH2N3OOH4OH + 0.0418ISOP1N2OOH3OH4OH + 0.0418ISOP1OH2OH3OOH4N + 0.0418ISOP1N2OH3OH4OOH + 0.0418ISOP1OOH2OH3OH4N + 0.551PROPNN + 0.551GLYC + 0.0103MACR2OH3N + 0.0103MACR2OH3N + 0.0103MACR2OH3N + 0.031HCHO + 0.582HO2 + 0.582OH : 2.6E-13*EXP(1300./TEMP);*
1.0NO + 1.0ISOP1N2OH3OO4OH = 0.935PROPNN + 0.935GLYC + 0.0217MACR2OH3N + 0.0217MACR4N + 0.0217MACR2N3N + 0.065HCHO + 1.0HO2 + 1.0NO2 : ALK(2.7E-12,350.,4.712,11.,1.,0.);
1.0NO + 1.0ISOP1N2OO3OH4OH = 0.935PROPNN + 0.935GLYC + 0.0217MACR2OH3N + 0.0217MACR4N + 0.0217MACR2N3N + 0.065HCHO + 1.0HO2 + 1.0NO2 : ALK(2.7E-12,350.,4.712,11.,1.,0.);
1.0NO + 1.0ISOP1N2OH3OO4OH = 0.167ISOP1OH2N3OH4N + 0.167ISOP1OH2N3N4OH + 0.167ISOP1N2OH3N4OH + 0.167ISOP1N2N3OH4OH + 0.167ISOP1OH2OH3N4N + 0.167ISOP1N2OH3OH4N : NIT(2.7E-12,350.,4.712,11.,1.,0.);
1.0NO + 1.0ISOP1N2OO3OH4OH = 0.167ISOP1OH2N3OH4N + 0.167ISOP1OH2N3N4OH + 0.167ISOP1N2OH3N4OH + 0.167ISOP1N2N3OH4OH + 0.167ISOP1OH2OH3N4N + 0.167ISOP1N2OH3OH4N : NIT(2.7E-12,350.,4.712,11.,1.,0.);
*1.0ISOP1N2OH3OO4OH = 0.0435ISOP1CO2N3OOH4OH + 0.0435ISOP1CO2OOH3N4OH + 0.0435ISOP1OH2OOH3N4CO + 0.0435ISOP1OH2N3OOH4CO + 0.0435ISOP1CO2N3OOH4OOH + 0.0435ISOP1CO2OOH3N4OOH + 0.0435ISOP1CO2OOH3OOH4N + 0.0435ISOP1OOH2OOH3N4CO + 0.0435ISOP1OOH2N3OOH4CO + 0.0435ISOP1N2OOH3OOH4CO + 0.0435ISOP1N2OOH3OH4CO + 0.0435ISOP1CO2OH3OOH4N + 0.0435ISOP1OOH2OH3CO4N + 0.0435ISOP1OOH2OH3N4CO + 0.0435ISOP1N2OH3CO4OOH + 0.0435ISOP1OH2OOH3CO4N + 0.0435ISOP1N4CO4OOH + 0.0435ISOP1N4CO4OH + 0.0435ISOP1CO1OOH4N + 0.0435ISOP1CO1OH4N + 0.0435ISOP1N2OH3OOH4CO + 0.0435ISOP1CO2OOH3OH4N + 0.0435ISOP1CO2N3OH4OOH + 1.0HO2 : 1.256E13*EXP(-10000./TEMP);*
1.0ISOP1N2OO3OH4OH = 0.0435ISOP1CO2N3OOH4OH + 0.0435ISOP1CO2OOH3N4OH + 0.0435ISOP1OH2OOH3N4CO + 0.0435ISOP1OH2N3OOH4CO + 0.0435ISOP1CO2N3OOH4OOH + 0.0435ISOP1CO2OOH3N4OOH + 0.0435ISOP1CO2OOH3OOH4N + 0.0435ISOP1OOH2OOH3N4CO + 0.0435ISOP1OOH2N3OOH4CO + 0.0435ISOP1N2OOH3OOH4CO + 0.0435ISOP1N2OOH3OH4CO + 0.0435ISOP1CO2OH3OOH4N + 0.0435ISOP1OOH2OH3CO4N + 0.0435ISOP1OOH2OH3N4CO + 0.0435ISOP1N2OH3CO4OOH + 0.0435ISOP1OH2OOH3CO4N + 0.0435ISOP1N4CO4OOH + 0.0435ISOP1N4CO4OH + 0.0435ISOP1CO1OOH4N + 0.0435ISOP1CO1OH4N +

*0.0435ISOP1N2OH3OOH4CO + 0.0435ISOP1CO2OOH3OH4N + 0.0435ISOP1CO2N3OH4OOH + 1.0HO2 : 1.256E13*EXP(-10000./TEMP);*
*IDHNBOO + HO2 = 0.379HO2 + 0.379OH + 0.0621ISOP1N2OOH3OH4OH + 0.0621ISOP1OH2OH3OOH4N + 0.0621ISOP1N2OH3OH4OOH + 0.0621ISOP1OOH2OH3OH4N + 0.0621ISOP1OH2N3OOH4OH + 0.0621ISOP1OH2OOH3OH4N + 0.0621ISOP1OH2N3OH4OOH + 0.0621ISOP1N2OH3OOH4OH + 0.0621ISOP1OOH2OH3N4OH + 0.0621ISOP1OH2OOH3N4OH + 0.094MACR2OH3N + 0.242GLYC + 0.242PROPNN + 0.010MVK3OH4N + 0.033HAC + 0.033ETHLN + 0.104HCHO : 2.6E-13*EXP(1300./TEMP);*
IDHNBOO + NO = 0.355MACR2OH3N + 0.546PROPNN + 0.546GLYC + 0.028MVK3OH4N + 0.071ETHLN + 0.071HAC + HO2 + NO2 + 0.383HCHO : ALK(2.7E-12,350.,1.851,11.,1.,0.);
IDHNBOO + NO = 0.16ISOP1OH2N3OH4N + 0.16ISOP1OH2N3N4OH + 0.16ISOP1N2OH3N4OH + 0.16ISOP1N2N3OH4OH + 0.16ISOP1OH2OH3N4N + 0.16ISOP1N2OH3OH4N : NIT(2.7E-12,350.,1.851,11.,1.,0.);
1.0OH + 1.0ISOP12O3OH4N = 0.201IHNEOO + 0.388ISOP1N2OH3OO4CO + 0.026ISOP1CO2OO3OH4N + 0.172ISOP1OH2OO3CO4N + 0.026MACRENOL + 0.187MVKENOL + 0.213NO2 + 0.213HCHO : EPO(3.22E-11,-400.,1.014E-20);
1.0OH + 1.0ISOP1OH23O4N = 0.201IHNEOO + 0.388ISOP1N2OH3OO4CO + 0.026ISOP1CO2OO3OH4N + 0.172ISOP1OH2OO3CO4N + 0.026MACRENOL + 0.187MVKENOL + 0.213NO2 + 0.213HCHO : EPO(3.22E-11,-400.,1.014E-20);
1.0OH + 1.0ISOP1N2OH34O = 0.201IHNEOO + 0.388ISOP1N2OH3OO4CO + 0.026ISOP1CO2OO3OH4N + 0.172ISOP1OH2OO3CO4N + 0.026MACRENOL + 0.187MVKENOL + 0.213NO2 + 0.213HCHO : EPO(3.22E-11,-400.,1.014E-20);
*1.0OH + 1.0ISOP12O3OH4N = 0.201IHNEOO + 0.2ISOP1N2OH3OO4CO + 0.2ISOP1N23O4CO + 0.2ISOP1CO23O4N + 0.2ISOP12O3CO4N : 7.77E-12*EXP(-400./TEMP);*
*1.0OH + 1.0ISOP1OH23O4N = 0.201IHNEOO + 0.2ISOP1N2OH3OO4CO + 0.2ISOP1N23O4CO + 0.2ISOP1CO23O4N + 0.2ISOP12O3CO4N : 7.77E-12*EXP(-400./TEMP);*
*1.0OH + 1.0ISOP1N2OH34O = 0.201IHNEOO + 0.2ISOP1N2OH3OO4CO + 0.2ISOP1N23O4CO + 0.2ISOP1CO23O4N + 0.2ISOP12O3CO4N : 7.77E-12*EXP(-400./TEMP);*
*IHNEOO + HO2 = 0.0348ISOP1CO2N3OOH4OH + 0.0348ISOP1CO2OOH3N4OH + 0.0348ISOP1OH2OOH3N4CO + 0.0348ISOP1OH2N3OOH4CO + 0.0348ISOP1CO2N3OOH4OOH + 0.0348ISOP1CO2OOH3N4OOH + 0.0348ISOP1CO2OOH3OOH4N + 0.0348ISOP1OOH2OOH3N4CO + 0.0348ISOP1OOH2N3OOH4CO + 0.0348ISOP1N2OOH3OOH4CO + 0.0348ISOP1N2OOH3OH4CO + 0.0348ISOP1CO2OH3OOH4N + 0.0348ISOP1OOH2OH3CO4N + 0.0348ISOP1OOH2OH3N4CO + 0.0348ISOP1N2OH3CO4OOH + 0.0348ISOP1OH2OOH3CO4N + 0.0348ISOP1N4CO4OOH + 0.0348ISOP1N4CO4OH + 0.0348ISOP1CO1OOH4N + 0.0348ISOP1CO1OH4N + 0.0348ISOP1N2OH3OOH4CO + 0.0348ISOP1CO2OOH3OH4N + 0.0348ISOP1CO2N3OH4OOH + 0.2PROPNN + 0.2CO + 0.2HO2 + 0.2HCHO + 0.2OH : 2.6E-13*EXP(1300./TEMP);*
IHNEOO + NO = 0.2ISOP1N2OH3CO4N + 0.2ISOP1OH2N3CO4N + 0.2ISOP1N2N3CO4OH + 0.2ISOP1CO2N3OH4N + 0.2ISOP1N2OH3N4CO : NIT(2.7E-12,350.,15.73,11.,1.,0.);
IHNEOO + NO = PROPNN + CO + HO2 + HCHO + NO2 : ALK(2.7E-12,350.,15.73,11.,1.,0.);
*1.0OH + 1.0ISOP1CO23O4OOH = 1.0OH + 1.5CO + 0.5HCHO + 0.5MGLY + 0.5HAC : 9.85E-12*EXP(410./TEMP) ;*
*1.0OH + 1.0ISOP1OOH23O4CO = 1.0OH + 1.5CO + 0.5HCHO + 0.5MGLY + 0.5HAC : 9.85E-12*EXP(410./TEMP) ;*
*1.0OH + 1.0ISO1OH12O = 1.0OH + 1.5CO + 0.5HCHO + 0.5MGLY + 0.5HAC : 9.85E-12*EXP(410./TEMP) ;*
*1.0OH + 1.0ISOP1OH23O4CO = 1.0OH + 1.5CO + 0.5HCHO + 0.5MGLY + 0.5HAC : 9.85E-12*EXP(410./TEMP) ;*

1.0OH + 1.0ISOP1CO23O4OH = 1.0OH + 1.5CO + 0.5HCHO + 0.5MGLY + 0.5HAC :
9.85E-12*EXP(410./TEMP) ;
1.0OH + 1.0ISOP1CO2OH34O = 1.0OH + 1.5CO + 0.5HCHO + 0.5MGLY + 0.5HAC :
9.85E-12*EXP(410./TEMP) ;
1.0OH + 1.0ISOP12O3OH4CO = 1.0OH + 1.5CO + 0.5HCHO + 0.5MGLY + 0.5HAC :
9.85E-12*EXP(410./TEMP) ;
1.0OH + 1.0ISOP12O3CO4OH = 1.0OH + 1.5CO + 0.5HCHO + 0.5MGLY + 0.5HAC :
9.85E-12*EXP(410./TEMP) ;
1.0OH + 1.0ISOP12O3OH4OOH = 1.0OH + 1.5CO + 0.5HCHO + 0.5MGLY + 0.5HAC :
9.85E-12*EXP(410./TEMP) ;
1.0OH + 1.0ISOP1OOH2OH34O = 1.0OH + 1.5CO + 0.5HCHO + 0.5MGLY + 0.5HAC :
9.85E-12*EXP(410./TEMP) ;
1.0OH + 1.0ISOP1N23O4CO = 1.0OH + 2.0CO + 0.35PROPNN + 0.65MGLY + 0.65HO2 + 0.65NO2 :
9.85E-12*EXP(410./TEMP) ;
1.0OH + 1.0ISOP1CO23O4N = 1.0OH + 2.0CO + 0.35PROPNN + 0.65MGLY + 0.65HO2 + 0.65NO2 :
9.85E-12*EXP(410./TEMP) ;
1.0OH + 1.0ISOP12O3CO4N = 1.0OH + 2.0CO + 0.35PROPNN + 0.65MGLY + 0.65HO2 + 0.65NO2 :
9.85E-12*EXP(410./TEMP) ;
1.0MVK3N4OH4OH = 1.0CH3CO3 + 1.0GLYC + 1.0NO2 : SUN*6.46E-5;
1.0MVK3N4N = 1.0CH3CO3 + 1.0GLYC + 1.0NO2 : SUN*6.46E-5;
1.0OH + 1.0MVK3N4OH4OH = 0.65HCOOH + 1.0NO3 + 0.65MGLY + 0.35HCHO + 0.35PYRAC :
1.5E-12*EXP(380/TEMP) ;
1.0OH + 1.0MVK3N4N = 0.65HCOOH + 1.0NO3 + 0.65MGLY + 0.35HCHO + 0.35PYRAC :
1.5E-12*EXP(380/TEMP) ;
1.0MVK3OOH4OH4OH = 1.0CH3CO3 + 1.0GLYC + 1.0OH : SUN*3.0E-5;
1.0OH + 1.0MVK3OOH4OH4OH = 1.0MVK3CO4OH + 1.0OH : 5.77E-11 ;
1.0OH + 1.0MVK3OH4OOH = 1.0MVK3OOH4CO4OOH + 1.0OH : 5.77E-11 ;
1.0OH + 1.0MVK3OH3OOH = 1.0MVK3OOH4CO4OOH + 1.0OH : 5.77E-11 ;
1.0MVK3OH4OOH = 1.0OH + 1.0HO2 + 1.0HCHO + 1.0MGLY : SUN*3.0E-5;
1.0MVK3OH3OOH = 1.0OH + 1.0HO2 + 1.0HCHO + 1.0MGLY : SUN*3.0E-5;
1.0MACR2OOH3OH = 1.0OH + 1.0CO + 1.0HO2 + 1.0HAC : SUN*3.0E-5;
1.0OH + 1.0MACR2OOH3OH = 1.0CO + 1.0OH + 1.0HAC : 2.7E-12*EXP(470./TEMP);
1.0MACR2OH3OOH = 1.0OH + 1.0HCHO + 1.0HO2 + 1.0MGLY : SUN*3.0E-5;
1.0MACR3OH3OOH = 1.0OH + 1.0HCHO + 1.0HO2 + 1.0MGLY : SUN*3.0E-5;
1.0OH + 1.0MACR2OH3OOH = 1.0CO2 + 1.0OH + 1.0HPAC : 2.7E-12*EXP(470./TEMP);
1.0OH + 1.0MACR3OH3OOH = 1.0CO2 + 1.0OH + 1.0HPAC : 2.7E-12*EXP(470./TEMP);
1.0OH + 1.0MACR2OH3N = 1.0CO2 + 1.0OH + 1.0PROPNN : 2.7E-12*EXP(470./TEMP);
1.0OH + 1.0MACR4N = 1.0CO2 + 1.0OH + 1.0PROPNN : 2.7E-12*EXP(470./TEMP);
1.0OH + 1.0MACR2N3N = 1.0CO2 + 1.0OH + 1.0PROPNN : 2.7E-12*EXP(470./TEMP);
1.0MVK3OOH4N = 1.0CH3CO3 + 1.0OH + 1.0ETHLN : SUN*4.21E-5;
1.0OH + 1.0MVK3OOH4N = 1.0OH + 1.0MVK3CO4N : 5.77E-11 ;
1.0OH + 1.0MACR2OOH3N = 1.0CO + 1.0OH + 1.0PROPNN : 2.7E-12*EXP(470./TEMP);
1.0MACR2OOH3N = 1.0PROPNN + 1.0OH + 1.0CO + 1.0HO2 : SUN*3.0E-5;
1.0MVK3CO4N = 2.0CH3CO3 + 1.0NO2 : 2.5E-4*SUN;
1.0OH + 1.0ISOP1CO4CO = 1.0CO + 1.0HO2 + 1.0MVK3OOH4CO : 3.0E-12*EXP(650./TEMP) ;
1.0OH + 1.0HPETHNL = 1.0CO + 1.0OH + 1.0HCHO : 1.55E-12*EXP(340./TEMP) ;
1.0OH + 1.0HPETHNL = 1.0GLYX + 1.0OH : 2.91E-11 ;
1.0HPETHNL = 1.0OH + 1.0CO + 1.0HO2 + 1.0HCHO : 2.5E-4*SUN;
1.0OH + 1.0HMML = 0.7MGLY + 0.7OH + 0.3CH3CO3 + 0.3HCOOH : 4.33E-12 ;

*1.0MACR1OOH = 1.0OH + 1.65CO2 + 0.65CH3OO + 1.0HCHO + 0.35CH3CO3 : SUN*3.0E-5;*
1.0OH + 1.0MACR1OH = 1.65CO2 + 1.0HCHO + 0.35CH3CO3 + 0.65CH3OO : 1.51E-11 ;
*1.0HPAC = 1.0CH3CO3 + 1.0HCHO + 1.0OH : SUN*3.0E-5;*
1.0OH + 1.0HPAC = 1.0MGLY + 1.0OH : 8.39E-12 ;
*1.0ISOP1N2N = 0.1MVK + 0.01MACR + 0.11HCHO + 1.11NO2 + 0.455ISOP1N4O + 0.455ISOP1CO4N + 0.455HO2 : SUN*5.0E-5;*
*1.0ISOP1N4N = 0.1MVK + 0.01MACR + 0.11HCHO + 1.11NO2 + 0.455ISOP1N4O + 0.455ISOP1CO4N + 0.455HO2 : SUN*5.0E-5;*
*1.0ISOP3N4N = 0.1MVK + 0.01MACR + 0.11HCHO + 1.11NO2 + 0.455ISOP1N4O + 0.455ISOP1CO4N + 0.455HO2 : SUN*5.0E-5;*
1.0OH + 1.0ISOP1N2N = 1.0NO2 + 0.333ISOP12O3OH4N + 0.333ISOP1OH23O4N + 0.333ISOP1N2OH34O : EPO(2.37E-11,390.,2.715E-19);
1.0OH + 1.0ISOP1N4N = 1.0NO2 + 0.333ISOP12O3OH4N + 0.333ISOP1OH23O4N + 0.333ISOP1N2OH34O : EPO(2.37E-11,390.,2.715E-19);
1.0OH + 1.0ISOP3N4N = 1.0NO2 + 0.333ISOP12O3OH4N + 0.333ISOP1OH23O4N + 0.333ISOP1N2OH34O : EPO(2.37E-11,390.,2.715E-19);
*1.0OH + 1.0ISOP1N2N = 1.0IDNOO : 0.74*2.37E-11*EXP(390./TEMP) ;*
*1.0OH + 1.0ISOP1N4N = 1.0IDNOO : 0.74*2.37E-11*EXP(390./TEMP) ;*
*1.0OH + 1.0ISOP3N4N = 1.0IDNOO : 0.74*2.37E-11*EXP(390./TEMP) ;*
*IDNOO + NO = PROPNN + 1.11NO2 + 0.11GLYC + 0.89ETHLN + 0.89HO2 : 2.7E-12*EXP(350./TEMP);*
*IDNOO + HO2 = 0.018ISOP1N2N3OOH4OH + 0.018ISOP1OH2OOH3N4N + 0.018ISOP1OOH2OH3N4N + 0.018ISOP1OOH2N3OH4N + 0.018ISOP1N2OH3OOH4N + 0.018ISOP1N2OOH3OH4N + 0.018ISOP1N2OH3N4OOH + 0.018ISOP1N2N3OH4OOH + 0.018ISOP1OH2N3OOH4N + 0.018ISOP1N2OOH3N4OH + 0.82PROPNN + 0.82OH + 0.09GLYC + 0.09NO2 + 0.73ETHLN + 0.73HO2 : 2.71E-13*EXP(1300./TEMP);*
*MACRNO2 + HO2 = 0.5HAC + 0.5OH + 0.5NO2 + 0.5CO2 + 0.13O3 + 0.13MACRNOH + 0.37MACRNOOH : 3.14E-12*EXP(580./TEMP) ;*
*MACRNO2 + NO = HAC + 2NO2 + CO2 : 7.5E-12*EXP(290./TEMP) ;*
MACRNO2 + NO2 = MPANHN : TROE(2.591E-28,0.,-6.87,1.125E-11,0.,-1.105,0.3);
MACRNO2 + NO3 = HAC + 2NO2 + CO2 : 4.0E-12 ;
*MACRNO2 + CH3OO = 0.7HAC + 0.7CO2 + 0.7NO2 + 0.7HO2 + HCHO + 0.3MACRNOH : 2.9E-12*EXP(500./TEMP) ;*
*1.0HO2 + 1.0MACR2N3OH = 0.5HAC + 0.5OH + 0.5NO2 + 0.5CO2 + 0.13O3 + 0.13MACRNOH + 0.37MACRNOOH : 3.14E-12*EXP(580./TEMP) ;*
*1.0NO + 1.0MACR2N3OH = 1.0HAC + 2.0NO2 + 1.0CO2 : 7.5E-12*EXP(290./TEMP) ;*
1.0NO2 + 1.0MACR2N3OH = 1.0MPAN1OH2OOH : TROE(2.591E-28,0.,-6.87,1.125E-11,0.,-1.105,0.3);
1.0NO3 + 1.0MACR2N3OH = 1.0HAC + 2.0NO2 + 1.0CO2 : 4.0E-12 ;
*1.0CH3OO + 1.0MACR2N3OH = 0.7HAC + 0.7CO2 + 0.7NO2 + 0.7HO2 + 1.0HCHO + 0.3MACRNOH : 2.9E-12*EXP(500./TEMP) ;*
*1.0HO2 + 1.0MACR2OH3N = 0.5HAC + 0.5OH + 0.5NO2 + 0.5CO2 + 0.13O3 + 0.13MACRNOH + 0.37MACRNOOH : 3.14E-12*EXP(580./TEMP) ;*
*1.0NO + 1.0MACR2OH3N = 1.0HAC + 2.0NO2 + 1.0CO2 : 7.5E-12*EXP(290./TEMP) ;*
1.0NO2 + 1.0MACR2OH3N = 1.0MPAN1OH2OOH : TROE(2.591E-28,0.,-6.87,1.125E-11,0.,-1.105,0.3);
1.0NO3 + 1.0MACR2OH3N = 1.0HAC + 2.0NO2 + 1.0CO2 : 4.0E-12 ;
*1.0CH3OO + 1.0MACR2OH3N = 0.7HAC + 0.7CO2 + 0.7NO2 + 0.7HO2 + 1.0HCHO + 0.3MACRNOH : 2.9E-12*EXP(500./TEMP) ;*
MACRNOH + OH = HAC + NO2 + CO2 : 1.34E-12 ;
MACRNOOH + OH = MACRNO2 : 4.42E-12 ;
*MACRNOOH = HAC + NO2 + OH + CO2 : SUN*6.49E-6 ;*

*MPANHN = MACRNO2 + NO2 : 1.58E16*EXP(-13500./TEMP);*
*1.0MPAN1OH2OOH = 1.0MACR2N3OH + 1.0NO2 : 1.58E16*EXP(-13500./TEMP);*
*1.0ETHLN = 1.0NO2 + 1.0HCHO + 1.0CO + 1.0HO2 : SUN*6.46E-5 ;*
1.0OH + 1.0ETHLN = 1.0NCH2CO3 : 3.4E-12 ;
*1.0NO3 + 1.0ETHLN = 1.0HNO3 + 1.0NCH2CO3 : 1.4E-12*EXP(-1860./TEMP);*
1.0OH + 1.0PYRAC = 1.0CH3CO3 + 1.0CO2 : 8.0E-13 ;
*1.0PYRAC = 1.0CH3CO3 + 1.0CO2 + 1.0HO2 : SUN*3.1E-3 ;*
1.0OH + 1.0PROPNN = 1.0MGLY + 1.0NO2 : 1.0E-13 ;
*1.0PROPNN = 1.0NO2 + 1.0HCHO + 1.0CH3CO3 : SUN*8.44E-6;*
1.0HO2 + 1.0CH3CO3 = 0.13O3 + 0.13CH3CO2H + 0.37CH3CO3H + 0.5CH3OO + 0.5CO2 + 0.5OH :
*3.14E-12*EXP(580./TEMP) ;*
*1.0NO + 1.0CH3CO3 = 1.0NO2 + 1.0CH3OO + 1.0CO2 : 7.5E-12*EXP(290./TEMP) ;*
1.0NO2 + 1.0CH3CO3 = 1.0PAN : TROE(2.591E-28,0.,-6.87,1.125E-11,0.,-1.105,0.3);
1.0NO3 + 1.0CH3CO3 = 1.0NO2 + 1.0CH3OO + 1.0CO2 : 4.0E-12 ;
1.0CH3OO + 1.0CH3CO3 = 0.7HO2 + 1.0HCHO + 0.7CO2 + 0.7CH3OO + 0.3CH3CO2H :
*2.9E-12*EXP(500./TEMP) ;*
NCH2CO3 + HO2 = 0.5HCHO + 0.5NO2 + 0.5OH + 0.5CO2 + 0.13O3 + 0.13NCH2CO2H +
*0.37NCH2CO3H : 3.14E-12*EXP(580./TEMP);*
*NCH2CO3 + NO = CO2 + HCHO + 2NO2 : 7.5E-12*EXP(290./TEMP) ;*
NCH2CO3 + NO2 = PNAN : TROE(2.591E-28,0.,-6.87,1.125E-11,0.,-1.105,0.3);
*NCH2CO3 + NO3 = 2NO2 + HCHO + CO2 : 2.3E-12*1.74 ;*
NCH2CO3 + CH3OO = 1.7HCHO + 0.7NO2 + 0.7CO2 + 0.7HO2 + 0.3NCH2CO2H : 1.0E-11 ;
PHAN = NO2 + HOCH2CO3 : TROE(3.871E-3,-12100.,0.,5.4E16,-13830.,0.,0.3);
PHAN + OH = HCHO + CO + NO2 : 1.12E-12 ;
NCH2CO3H + OH = NCH2CO3 : 3.63E-12 ;
*NCH2CO3H = HCHO + NO2 + OH + CO2 : SUN*3.0E-5 ;*
PNAN = NCH2CO3 + NO2 : TROE(3.871E-3,-12100.,0.,5.4E16,-13830.,0.,0.3);
PNAN + OH = HCHO + CO + 2NO2 : 1.12E-14 ;
1.0OH + 1.0ISOP1CO2N3OOH4OH = 1.0CO + 1.0NO2 + 0.75MVK3OOH4OH4OH +
0.25MACR2OOH3OH : 1.0E-11 ;
1.0OH + 1.0ISOP1CO2OOH3N4OH = 1.0CO + 1.0NO2 + 0.75MVK3OOH4OH4OH +
0.25MACR2OOH3OH : 1.0E-11 ;
1.0OH + 1.0ISOP1OH2OOH3N4CO = 1.0CO + 1.0NO2 + 0.75MVK3OOH4OH4OH +
0.25MACR2OOH3OH : 1.0E-11 ;
1.0OH + 1.0ISOP1OH2N3OOH4CO = 1.0CO + 1.0NO2 + 0.75MVK3OOH4OH4OH +
0.25MACR2OOH3OH : 1.0E-11 ;
1.0OH + 1.0ISOP1CO2N3OOH4OOH = 1.0CO + 1.0NO2 + 0.75MVK3OOH4OH4OH +
0.25MACR2OOH3OH : 1.0E-11 ;
1.0OH + 1.0ISOP1CO2OOH3N4OOH = 1.0CO + 1.0NO2 + 0.75MVK3OOH4OH4OH +
0.25MACR2OOH3OH : 1.0E-11 ;
1.0OH + 1.0ISOP1CO2OOH3OOH4N = 1.0CO + 1.0NO2 + 0.75MVK3OOH4OH4OH +
0.25MACR2OOH3OH : 1.0E-11 ;
1.0OH + 1.0ISOP1OOH2OOH3N4CO = 1.0CO + 1.0NO2 + 0.75MVK3OOH4OH4OH +
0.25MACR2OOH3OH : 1.0E-11 ;
1.0OH + 1.0ISOP1OOH2N3OOH4CO = 1.0CO + 1.0NO2 + 0.75MVK3OOH4OH4OH +
0.25MACR2OOH3OH : 1.0E-11 ;
1.0OH + 1.0ISOP1N2OOH3OOH4CO = 1.0CO + 1.0NO2 + 0.75MVK3OOH4OH4OH +
0.25MACR2OOH3OH : 1.0E-11 ;

1.0OH + 1.0ISOP1N2OOH3OH4CO = 1.0CO + 1.0NO2 + 0.75MVK3OOH4OH4OH + 0.25MACR2OOH3OH : 1.0E-11 ;
1.0OH + 1.0ISOP1CO2OH3OOH4N = 1.0CO + 1.0NO2 + 0.75MVK3OOH4OH4OH + 0.25MACR2OOH3OH : 1.0E-11 ;
1.0OH + 1.0ISOP1OOH2OH3CO4N = 1.0CO + 1.0NO2 + 0.75MVK3OOH4OH4OH + 0.25MACR2OOH3OH : 1.0E-11 ;
1.0OH + 1.0ISOP1CO2N3OH4OOH = 1.0CO + 1.0NO2 + 0.75MVK3OOH4OH4OH + 0.25MACR2OOH3OH : 1.0E-11 ;
1.0OH + 1.0ISOP1OOH2OH3N4CO = 1.0CO + 1.0NO2 + 0.75MVK3OOH4OH4OH + 0.25MACR2OOH3OH : 1.0E-11 ;
1.0OH + 1.0ISOP1N2OH3CO4OOH = 1.0CO + 1.0NO2 + 0.75MVK3OOH4OH4OH + 0.25MACR2OOH3OH : 1.0E-11 ;
1.0OH + 1.0ISOP1OH2OOH3CO4N = 1.0CO + 1.0NO2 + 0.75MVK3OOH4OH4OH + 0.25MACR2OOH3OH : 1.0E-11 ;
1.0OH + 1.0ISOP1N4CO4OOH = 1.0CO + 1.0NO2 + 0.75MVK3OOH4OH4OH + 0.25MACR2OOH3OH : 1.0E-11 ;
1.0OH + 1.0ISOP1N4CO4OH = 1.0CO + 1.0NO2 + 0.75MVK3OOH4OH4OH + 0.25MACR2OOH3OH : 1.0E-11 ;
1.0OH + 1.0ISOP1CO1OOH4N = 1.0CO + 1.0NO2 + 0.75MVK3OOH4OH4OH + 0.25MACR2OOH3OH : 1.0E-11 ;
1.0OH + 1.0ISOP1CO1OH4N = 1.0CO + 1.0NO2 + 0.75MVK3OOH4OH4OH + 0.25MACR2OOH3OH : 1.0E-11 ;
1.0OH + 1.0ISOP1N2OH3OOH4CO = 1.0CO + 1.0NO2 + 0.75MVK3OOH4OH4OH + 0.25MACR2OOH3OH : 1.0E-11 ;
1.0OH + 1.0ISOP1CO2OOH3OH4N = 1.0CO + 1.0NO2 + 0.75MVK3OOH4OH4OH + 0.25MACR2OOH3OH : 1.0E-11 ;
1.0ISOP1CO2N3OOH4OH = 1.0MGLY + 1.0OH + 1.0NO2 + 1.0GLYC : SUN(1.15E-5) ;*
1.0ISOP1CO2OOH3N4OH = 1.0MGLY + 1.0OH + 1.0NO2 + 1.0GLYC : SUN(1.15E-5) ;*
1.0ISOP1OH2OOH3N4CO = 1.0MGLY + 1.0OH + 1.0NO2 + 1.0GLYC : SUN(1.15E-5) ;*
1.0ISOP1OH2N3OOH4CO = 1.0MGLY + 1.0OH + 1.0NO2 + 1.0GLYC : SUN(1.15E-5) ;*
1.0ISOP1CO2N3OOH4OOH = 1.0MGLY + 1.0OH + 1.0NO2 + 1.0GLYC : SUN(1.15E-5) ;*
1.0ISOP1CO2OOH3N4OOH = 1.0MGLY + 1.0OH + 1.0NO2 + 1.0GLYC : SUN(1.15E-5) ;*
1.0ISOP1CO2OOH3OOH4N = 1.0MGLY + 1.0OH + 1.0NO2 + 1.0GLYC : SUN(1.15E-5) ;*
1.0ISOP1OOH2OOH3N4CO = 1.0MGLY + 1.0OH + 1.0NO2 + 1.0GLYC : SUN(1.15E-5) ;*
1.0ISOP1OOH2N3OOH4CO = 1.0MGLY + 1.0OH + 1.0NO2 + 1.0GLYC : SUN(1.15E-5) ;*
1.0ISOP1N2OOH3OOH4CO = 1.0MGLY + 1.0OH + 1.0NO2 + 1.0GLYC : SUN(1.15E-5) ;*
1.0ISOP1N2OOH3OH4CO = 1.0MGLY + 1.0OH + 1.0NO2 + 1.0GLYC : SUN(1.15E-5) ;*
1.0ISOP1CO2OH3OOH4N = 1.0MGLY + 1.0OH + 1.0NO2 + 1.0GLYC : SUN(1.15E-5) ;*
1.0ISOP1OOH2OH3CO4N = 1.0MGLY + 1.0OH + 1.0NO2 + 1.0GLYC : SUN(1.15E-5) ;*
1.0ISOP1CO2N3OH4OOH = 1.0MGLY + 1.0OH + 1.0NO2 + 1.0GLYC : SUN(1.15E-5) ;*
1.0ISOP1OOH2OH3N4CO = 1.0MGLY + 1.0OH + 1.0NO2 + 1.0GLYC : SUN(1.15E-5) ;*
1.0ISOP1N2OH3CO4OOH = 1.0MGLY + 1.0OH + 1.0NO2 + 1.0GLYC : SUN(1.15E-5) ;*
1.0ISOP1OH2OOH3CO4N = 1.0MGLY + 1.0OH + 1.0NO2 + 1.0GLYC : SUN(1.15E-5) ;*
1.0ISOP1N4CO4OOH = 1.0MGLY + 1.0OH + 1.0NO2 + 1.0GLYC : SUN(1.15E-5) ;*
1.0ISOP1N4CO4OH = 1.0MGLY + 1.0OH + 1.0NO2 + 1.0GLYC : SUN(1.15E-5) ;*
1.0ISOP1CO1OOH4N = 1.0MGLY + 1.0OH + 1.0NO2 + 1.0GLYC : SUN(1.15E-5) ;*
1.0ISOP1CO1OH4N = 1.0MGLY + 1.0OH + 1.0NO2 + 1.0GLYC : SUN(1.15E-5) ;*
1.0ISOP1N2OH3OOH4CO = 1.0MGLY + 1.0OH + 1.0NO2 + 1.0GLYC : SUN(1.15E-5) ;*
1.0ISOP1CO2OOH3OH4N = 1.0MGLY + 1.0OH + 1.0NO2 + 1.0GLYC : SUN(1.15E-5) ;*

*1.0ISOP1CO2N3OOH4OH = 0.5MVK3OOH4OH4OH + 0.5MACR2OOH3OH + 1.0CO + 1.0NO2 + 1.0HO2 : SUN*3.0E-5 ;*
*1.0ISOP1CO2OOH3N4OH = 0.5MVK3OOH4OH4OH + 0.5MACR2OOH3OH + 1.0CO + 1.0NO2 + 1.0HO2 : SUN*3.0E-5 ;*
*1.0ISOP1OH2OOH3N4CO = 0.5MVK3OOH4OH4OH + 0.5MACR2OOH3OH + 1.0CO + 1.0NO2 + 1.0HO2 : SUN*3.0E-5 ;*
*1.0ISOP1OH2N3OOH4CO = 0.5MVK3OOH4OH4OH + 0.5MACR2OOH3OH + 1.0CO + 1.0NO2 + 1.0HO2 : SUN*3.0E-5 ;*
*1.0ISOP1CO2N3OOH4OOH = 0.5MVK3OOH4OH4OH + 0.5MACR2OOH3OH + 1.0CO + 1.0NO2 + 1.0HO2 : SUN*3.0E-5 ;*
*1.0ISOP1CO2OOH3N4OOH = 0.5MVK3OOH4OH4OH + 0.5MACR2OOH3OH + 1.0CO + 1.0NO2 + 1.0HO2 : SUN*3.0E-5 ;*
*1.0ISOP1CO2OOH3OOH4N = 0.5MVK3OOH4OH4OH + 0.5MACR2OOH3OH + 1.0CO + 1.0NO2 + 1.0HO2 : SUN*3.0E-5 ;*
*1.0ISOP1OOH2OOH3N4CO = 0.5MVK3OOH4OH4OH + 0.5MACR2OOH3OH + 1.0CO + 1.0NO2 + 1.0HO2 : SUN*3.0E-5 ;*
*1.0ISOP1OOH2N3OOH4CO = 0.5MVK3OOH4OH4OH + 0.5MACR2OOH3OH + 1.0CO + 1.0NO2 + 1.0HO2 : SUN*3.0E-5 ;*
*1.0ISOP1N2OOH3OOH4CO = 0.5MVK3OOH4OH4OH + 0.5MACR2OOH3OH + 1.0CO + 1.0NO2 + 1.0HO2 : SUN*3.0E-5 ;*
*1.0ISOP1N2OOH3OH4CO = 0.5MVK3OOH4OH4OH + 0.5MACR2OOH3OH + 1.0CO + 1.0NO2 + 1.0HO2 : SUN*3.0E-5 ;*
*1.0ISOP1CO2OH3OOH4N = 0.5MVK3OOH4OH4OH + 0.5MACR2OOH3OH + 1.0CO + 1.0NO2 + 1.0HO2 : SUN*3.0E-5 ;*
*1.0ISOP1OOH2OH3CO4N = 0.5MVK3OOH4OH4OH + 0.5MACR2OOH3OH + 1.0CO + 1.0NO2 + 1.0HO2 : SUN*3.0E-5 ;*
*1.0ISOP1CO2N3OH4OOH = 0.5MVK3OOH4OH4OH + 0.5MACR2OOH3OH + 1.0CO + 1.0NO2 + 1.0HO2 : SUN*3.0E-5 ;*
*1.0ISOP1OOH2OH3N4CO = 0.5MVK3OOH4OH4OH + 0.5MACR2OOH3OH + 1.0CO + 1.0NO2 + 1.0HO2 : SUN*3.0E-5 ;*
*1.0ISOP1N2OH3CO4OOH = 0.5MVK3OOH4OH4OH + 0.5MACR2OOH3OH + 1.0CO + 1.0NO2 + 1.0HO2 : SUN*3.0E-5 ;*
*1.0ISOP1OH2OOH3CO4N = 0.5MVK3OOH4OH4OH + 0.5MACR2OOH3OH + 1.0CO + 1.0NO2 + 1.0HO2 : SUN*3.0E-5 ;*
*1.0ISOP1N4CO4OOH = 0.5MVK3OOH4OH4OH + 0.5MACR2OOH3OH + 1.0CO + 1.0NO2 + 1.0HO2 : SUN*3.0E-5 ;*
*1.0ISOP1N4CO4OH = 0.5MVK3OOH4OH4OH + 0.5MACR2OOH3OH + 1.0CO + 1.0NO2 + 1.0HO2 : SUN*3.0E-5 ;*
*1.0ISOP1CO1OOH4N = 0.5MVK3OOH4OH4OH + 0.5MACR2OOH3OH + 1.0CO + 1.0NO2 + 1.0HO2 : SUN*3.0E-5 ;*
*1.0ISOP1CO1OH4N = 0.5MVK3OOH4OH4OH + 0.5MACR2OOH3OH + 1.0CO + 1.0NO2 + 1.0HO2 : SUN*3.0E-5 ;*
*1.0ISOP1N2OH3OOH4CO = 0.5MVK3OOH4OH4OH + 0.5MACR2OOH3OH + 1.0CO + 1.0NO2 + 1.0HO2 : SUN*3.0E-5 ;*
*1.0ISOP1CO2OOH3OH4N = 0.5MVK3OOH4OH4OH + 0.5MACR2OOH3OH + 1.0CO + 1.0NO2 + 1.0HO2 : SUN*3.0E-5 ;*
1.0OH + 1.0ISOP1N2OOH3OOH4OH = 1.0OH + 0.0435ISOP1CO2N3OOH4OH + 0.0435ISOP1CO2OOH3N4OH + 0.0435ISOP1OH2OOH3N4CO + 0.0435ISOP1OH2N3OOH4CO + 0.0435ISOP1CO2N3OOH4OOH + 0.0435ISOP1CO2OOH3N4OOH + 0.0435ISOP1CO2OOH3OOH4N +

0.0435ISOP1OOH2OOH3N4CO + 0.0435ISOP1OOH2N3OOH4CO + 0.0435ISOP1N2OOH3OOH4CO + 0.0435ISOP1N2OOH3OH4CO + 0.0435ISOP1CO2OH3OOH4N + 0.0435ISOP1OOH2OH3CO4N + 0.0435ISOP1OOH2OH3N4CO + 0.0435ISOP1N2OH3CO4OOH + 0.0435ISOP1OH2OOH3CO4N + 0.0435ISOP1N4CO4OOH + 0.0435ISOP1N4CO4OH + 0.0435ISOP1CO1OOH4N + 0.0435ISOP1CO1OH4N + 0.0435ISOP1N2OH3OOH4CO + 0.0435ISOP1CO2OOH3OH4N + 0.0435ISOP1CO2N3OH4OOH : 2.0E-12 ;
1.0OH + 1.0ISOP1N2OOH3OH4OOH = 1.0OH + 0.0435ISOP1CO2N3OOH4OH + 0.0435ISOP1CO2OOH3N4OH + 0.0435ISOP1OH2OOH3N4CO + 0.0435ISOP1OH2N3OOH4CO + 0.0435ISOP1CO2N3OOH4OOH + 0.0435ISOP1CO2OOH3N4OOH + 0.0435ISOP1CO2OOH3OOH4N + 0.0435ISOP1OOH2OOH3N4CO + 0.0435ISOP1OOH2N3OOH4CO + 0.0435ISOP1N2OOH3OOH4CO + 0.0435ISOP1N2OOH3OH4CO + 0.0435ISOP1CO2OH3OOH4N + 0.0435ISOP1OOH2OH3CO4N + 0.0435ISOP1OOH2OH3N4CO + 0.0435ISOP1N2OH3CO4OOH + 0.0435ISOP1OH2OOH3CO4N + 0.0435ISOP1N4CO4OOH + 0.0435ISOP1N4CO4OH + 0.0435ISOP1CO1OOH4N + 0.0435ISOP1CO1OH4N + 0.0435ISOP1N2OH3OOH4CO + 0.0435ISOP1CO2OOH3OH4N + 0.0435ISOP1CO2N3OH4OOH : 2.0E-12 ;
1.0OH + 1.0ISOP1N2OH3OOH4OOH = 1.0OH + 0.0435ISOP1CO2N3OOH4OH + 0.0435ISOP1CO2OOH3N4OH + 0.0435ISOP1OH2OOH3N4CO + 0.0435ISOP1OH2N3OOH4CO + 0.0435ISOP1CO2N3OOH4OOH + 0.0435ISOP1CO2OOH3N4OOH + 0.0435ISOP1CO2OOH3OOH4N + 0.0435ISOP1OOH2OOH3N4CO + 0.0435ISOP1OOH2N3OOH4CO + 0.0435ISOP1N2OOH3OOH4CO + 0.0435ISOP1N2OOH3OH4CO + 0.0435ISOP1CO2OH3OOH4N + 0.0435ISOP1OOH2OH3CO4N + 0.0435ISOP1OOH2OH3N4CO + 0.0435ISOP1N2OH3CO4OOH + 0.0435ISOP1OH2OOH3CO4N + 0.0435ISOP1N4CO4OOH + 0.0435ISOP1N4CO4OH + 0.0435ISOP1CO1OOH4N + 0.0435ISOP1CO1OH4N + 0.0435ISOP1N2OH3OOH4CO + 0.0435ISOP1CO2OOH3OH4N + 0.0435ISOP1CO2N3OH4OOH : 2.0E-12 ;
1.0OH + 1.0ISOP1OH2OOH3OOH4N = 1.0OH + 0.0435ISOP1CO2N3OOH4OH + 0.0435ISOP1CO2OOH3N4OH + 0.0435ISOP1OH2OOH3N4CO + 0.0435ISOP1OH2N3OOH4CO + 0.0435ISOP1CO2N3OOH4OOH + 0.0435ISOP1CO2OOH3N4OOH + 0.0435ISOP1CO2OOH3OOH4N + 0.0435ISOP1OOH2OOH3N4CO + 0.0435ISOP1OOH2N3OOH4CO + 0.0435ISOP1N2OOH3OOH4CO + 0.0435ISOP1N2OOH3OH4CO + 0.0435ISOP1CO2OH3OOH4N + 0.0435ISOP1OOH2OH3CO4N + 0.0435ISOP1OOH2OH3N4CO + 0.0435ISOP1N2OH3CO4OOH + 0.0435ISOP1OH2OOH3CO4N + 0.0435ISOP1N4CO4OOH + 0.0435ISOP1N4CO4OH + 0.0435ISOP1CO1OOH4N + 0.0435ISOP1CO1OH4N + 0.0435ISOP1N2OH3OOH4CO + 0.0435ISOP1CO2OOH3OH4N + 0.0435ISOP1CO2N3OH4OOH : 2.0E-12 ;
1.0OH + 1.0ISOP1OOH2OH3OOH4N = 1.0OH + 0.0435ISOP1CO2N3OOH4OH + 0.0435ISOP1CO2OOH3N4OH + 0.0435ISOP1OH2OOH3N4CO + 0.0435ISOP1OH2N3OOH4CO + 0.0435ISOP1CO2N3OOH4OOH + 0.0435ISOP1CO2OOH3N4OOH + 0.0435ISOP1CO2OOH3OOH4N + 0.0435ISOP1OOH2OOH3N4CO + 0.0435ISOP1OOH2N3OOH4CO + 0.0435ISOP1N2OOH3OOH4CO + 0.0435ISOP1N2OOH3OH4CO + 0.0435ISOP1CO2OH3OOH4N + 0.0435ISOP1OOH2OH3CO4N + 0.0435ISOP1OOH2OH3N4CO + 0.0435ISOP1N2OH3CO4OOH + 0.0435ISOP1OH2OOH3CO4N + 0.0435ISOP1N4CO4OOH + 0.0435ISOP1N4CO4OH + 0.0435ISOP1CO1OOH4N + 0.0435ISOP1CO1OH4N + 0.0435ISOP1N2OH3OOH4CO + 0.0435ISOP1CO2OOH3OH4N + 0.0435ISOP1CO2N3OH4OOH : 2.0E-12 ;
1.0OH + 1.0ISOP1OOH2OOH3OH4N = 1.0OH + 0.0435ISOP1CO2N3OOH4OH + 0.0435ISOP1CO2OOH3N4OH + 0.0435ISOP1OH2OOH3N4CO + 0.0435ISOP1OH2N3OOH4CO + 0.0435ISOP1CO2N3OOH4OOH + 0.0435ISOP1CO2OOH3N4OOH + 0.0435ISOP1CO2OOH3OOH4N + 0.0435ISOP1OOH2OOH3N4CO + 0.0435ISOP1OOH2N3OOH4CO + 0.0435ISOP1N2OOH3OOH4CO + 0.0435ISOP1N2OOH3OH4CO + 0.0435ISOP1CO2OH3OOH4N + 0.0435ISOP1OOH2OH3CO4N + 0.0435ISOP1OOH2OH3N4CO + 0.0435ISOP1N2OH3CO4OOH + 0.0435ISOP1OH2OOH3CO4N + 0.0435ISOP1N4CO4OOH + 0.0435ISOP1N4CO4OH + 0.0435ISOP1CO1OOH4N +

0.0435ISOP1CO1OH4N + 0.0435ISOP1N2OH3OOH4CO + 0.0435ISOP1CO2OOH3OH4N + 0.0435ISOP1CO2N3OH4OOH : 2.0E-12 ;
*1.0ISOP1N2OOH3OOH4OH = 1.0NO2 + 0.5ISOP1OH2OOH3OH4OO + 0.5ISOP1OH2OO3OH4OOH : SUN*5.0E-6 ;*
*1.0ISOP1N2OOH3OH4OOH = 1.0NO2 + 0.5ISOP1OH2OOH3OH4OO + 0.5ISOP1OH2OO3OH4OOH : SUN*5.0E-6 ;*
*1.0ISOP1N2OH3OOH4OOH = 1.0NO2 + 0.5ISOP1OH2OOH3OH4OO + 0.5ISOP1OH2OO3OH4OOH : SUN*5.0E-6 ;*
*1.0ISOP1OH2OOH3OOH4N = 1.0NO2 + 0.5ISOP1OH2OOH3OH4OO + 0.5ISOP1OH2OO3OH4OOH : SUN*5.0E-6 ;*
*1.0ISOP1OOH2OH3OOH4N = 1.0NO2 + 0.5ISOP1OH2OOH3OH4OO + 0.5ISOP1OH2OO3OH4OOH : SUN*5.0E-6 ;*
*1.0ISOP1OOH2OOH3OH4N = 1.0NO2 + 0.5ISOP1OH2OOH3OH4OO + 0.5ISOP1OH2OO3OH4OOH : SUN*5.0E-6 ;*
*1.0ISOP1N2OOH3OOH4OH = 1.0OH + 0.25ISOP1N2OH3OO4OH + 0.25ISOP1N2OO3OH4OH + 0.5IDHNBOO : SUN*1.3E-5 ;*
*1.0ISOP1N2OOH3OH4OOH = 1.0OH + 0.25ISOP1N2OH3OO4OH + 0.25ISOP1N2OO3OH4OH + 0.5IDHNBOO : SUN*1.3E-5 ;*
*1.0ISOP1N2OH3OOH4OOH = 1.0OH + 0.25ISOP1N2OH3OO4OH + 0.25ISOP1N2OO3OH4OH + 0.5IDHNBOO : SUN*1.3E-5 ;*
*1.0ISOP1OH2OOH3OOH4N = 1.0OH + 0.25ISOP1N2OH3OO4OH + 0.25ISOP1N2OO3OH4OH + 0.5IDHNBOO : SUN*1.3E-5 ;*
*1.0ISOP1OOH2OH3OOH4N = 1.0OH + 0.25ISOP1N2OH3OO4OH + 0.25ISOP1N2OO3OH4OH + 0.5IDHNBOO : SUN*1.3E-5 ;*
*1.0ISOP1OOH2OOH3OH4N = 1.0OH + 0.25ISOP1N2OH3OO4OH + 0.25ISOP1N2OO3OH4OH + 0.5IDHNBOO : SUN*1.3E-5 ;*
1.0OH + 1.0ISOP1OH2N3OH4N = 0.167ISOP1CO2N3OH4OH + 0.167ISOP1N2OH3CO4N + 0.167ISOP1OH2N3CO4N + 0.167ISOP1N2N3CO4OH + 0.167ISOP1N2OH3N4CO + 0.167ISOP1CO2N3OH4N : 2.0E-12 ;
1.0OH + 1.0ISOP1OH2N3N4OH = 0.167ISOP1CO2N3OH4OH + 0.167ISOP1N2OH3CO4N + 0.167ISOP1OH2N3CO4N + 0.167ISOP1N2N3CO4OH + 0.167ISOP1N2OH3N4CO + 0.167ISOP1CO2N3OH4N : 2.0E-12 ;
1.0OH + 1.0ISOP1N2OH3N4OH = 0.167ISOP1CO2N3OH4OH + 0.167ISOP1N2OH3CO4N + 0.167ISOP1OH2N3CO4N + 0.167ISOP1N2N3CO4OH + 0.167ISOP1N2OH3N4CO + 0.167ISOP1CO2N3OH4N : 2.0E-12 ;
1.0OH + 1.0ISOP1N2N3OH4OH = 0.167ISOP1CO2N3OH4OH + 0.167ISOP1N2OH3CO4N + 0.167ISOP1OH2N3CO4N + 0.167ISOP1N2N3CO4OH + 0.167ISOP1N2OH3N4CO + 0.167ISOP1CO2N3OH4N : 2.0E-12 ;
1.0OH + 1.0ISOP1OH2OH3N4N = 0.167ISOP1CO2N3OH4OH + 0.167ISOP1N2OH3CO4N + 0.167ISOP1OH2N3CO4N + 0.167ISOP1N2N3CO4OH + 0.167ISOP1N2OH3N4CO + 0.167ISOP1CO2N3OH4N : 2.0E-12 ;
1.0OH + 1.0ISOP1N2OH3OH4N = 0.167ISOP1CO2N3OH4OH + 0.167ISOP1N2OH3CO4N + 0.167ISOP1OH2N3CO4N + 0.167ISOP1N2N3CO4OH + 0.167ISOP1N2OH3N4CO + 0.167ISOP1CO2N3OH4N : 2.0E-12 ;
*1.0ISOP1OH2N3OH4N = 2.0NO2 + 1.0GLYC + 1.0HAC : SUN*5.0E-6*2 ;*
*1.0ISOP1OH2N3N4OH = 2.0NO2 + 1.0GLYC + 1.0HAC : SUN*5.0E-6*2 ;*
*1.0ISOP1N2OH3N4OH = 2.0NO2 + 1.0GLYC + 1.0HAC : SUN*5.0E-6*2 ;*
*1.0ISOP1N2N3OH4OH = 2.0NO2 + 1.0GLYC + 1.0HAC : SUN*5.0E-6*2 ;*
*1.0ISOP1OH2OH3N4N = 2.0NO2 + 1.0GLYC + 1.0HAC : SUN*5.0E-6*2 ;*

*1.0ISOP1N2OH3OH4N = 2.0NO2 + 1.0GLYC + 1.0HAC : SUN*5.0E-6*2 ;*
1.0OH + 1.0ISOP1OH2OOH3OH4N = 0.33OH + 0.67HO2 + 0.165ISOP1OH2N3CO4OH + 0.165ISOP1OH2OH3N4CO + 0.0291ISOP1CO2N3OOH4OH + 0.0291ISOP1CO2OOH3N4OH + 0.0291ISOP1OH2OOH3N4CO + 0.0291ISOP1OH2N3OOH4CO + 0.0291ISOP1CO2N3OOH4OOH + 0.0291ISOP1CO2OOH3N4OOH + 0.0291ISOP1CO2OOH3OOH4N + 0.0291ISOP1OOH2OOH3N4CO + 0.0291ISOP1OOH2N3OOH4CO + 0.0291ISOP1N2OOH3OOH4CO + 0.0291ISOP1N2OOH3OH4CO + 0.0291ISOP1CO2OH3OOH4N + 0.0291ISOP1OOH2OH3CO4N + 0.0291ISOP1OOH2OH3N4CO + 0.0291ISOP1N2OH3CO4OOH + 0.0291ISOP1OH2OOH3CO4N + 0.0291ISOP1N4CO4OOH + 0.0291ISOP1N4CO4OH + 0.0291ISOP1CO1OOH4N + 0.0291ISOP1CO1OH4N + 0.0291ISOP1N2OH3OOH4CO + 0.0291ISOP1CO2OOH3OH4N + 0.0291ISOP1CO2N3OH4OOH : 3.0E-12 ;
1.0OH + 1.0ISOP1OH2N3OH4OOH = 0.33OH + 0.67HO2 + 0.165ISOP1OH2N3CO4OH + 0.165ISOP1OH2OH3N4CO + 0.0291ISOP1CO2N3OOH4OH + 0.0291ISOP1CO2OOH3N4OH + 0.0291ISOP1OH2OOH3N4CO + 0.0291ISOP1OH2N3OOH4CO + 0.0291ISOP1CO2N3OOH4OOH + 0.0291ISOP1CO2OOH3N4OOH + 0.0291ISOP1CO2OOH3OOH4N + 0.0291ISOP1OOH2OOH3N4CO + 0.0291ISOP1OOH2N3OOH4CO + 0.0291ISOP1N2OOH3OOH4CO + 0.0291ISOP1N2OOH3OH4CO + 0.0291ISOP1CO2OH3OOH4N + 0.0291ISOP1OOH2OH3CO4N + 0.0291ISOP1OOH2OH3N4CO + 0.0291ISOP1N2OH3CO4OOH + 0.0291ISOP1OH2OOH3CO4N + 0.0291ISOP1N4CO4OOH + 0.0291ISOP1N4CO4OH + 0.0291ISOP1CO1OOH4N + 0.0291ISOP1CO1OH4N + 0.0291ISOP1N2OH3OOH4CO + 0.0291ISOP1CO2OOH3OH4N + 0.0291ISOP1CO2N3OH4OOH : 3.0E-12 ;
1.0OH + 1.0ISOP1N2OH3OOH4OH = 0.33OH + 0.67HO2 + 0.165ISOP1OH2N3CO4OH + 0.165ISOP1OH2OH3N4CO + 0.0291ISOP1CO2N3OOH4OH + 0.0291ISOP1CO2OOH3N4OH + 0.0291ISOP1OH2OOH3N4CO + 0.0291ISOP1OH2N3OOH4CO + 0.0291ISOP1CO2N3OOH4OOH + 0.0291ISOP1CO2OOH3N4OOH + 0.0291ISOP1CO2OOH3OOH4N + 0.0291ISOP1OOH2OOH3N4CO + 0.0291ISOP1OOH2N3OOH4CO + 0.0291ISOP1N2OOH3OOH4CO + 0.0291ISOP1N2OOH3OH4CO + 0.0291ISOP1CO2OH3OOH4N + 0.0291ISOP1OOH2OH3CO4N + 0.0291ISOP1OOH2OH3N4CO + 0.0291ISOP1N2OH3CO4OOH + 0.0291ISOP1OH2OOH3CO4N + 0.0291ISOP1N4CO4OOH + 0.0291ISOP1N4CO4OH + 0.0291ISOP1CO1OOH4N + 0.0291ISOP1CO1OH4N + 0.0291ISOP1N2OH3OOH4CO + 0.0291ISOP1CO2OOH3OH4N + 0.0291ISOP1CO2N3OH4OOH : 3.0E-12 ;
1.0OH + 1.0ISOP1OOH2OH3N4OH = 0.33OH + 0.67HO2 + 0.165ISOP1OH2N3CO4OH + 0.165ISOP1OH2OH3N4CO + 0.0291ISOP1CO2N3OOH4OH + 0.0291ISOP1CO2OOH3N4OH + 0.0291ISOP1OH2OOH3N4CO + 0.0291ISOP1OH2N3OOH4CO + 0.0291ISOP1CO2N3OOH4OOH + 0.0291ISOP1CO2OOH3N4OOH + 0.0291ISOP1CO2OOH3OOH4N + 0.0291ISOP1OOH2OOH3N4CO + 0.0291ISOP1OOH2N3OOH4CO + 0.0291ISOP1N2OOH3OOH4CO + 0.0291ISOP1N2OOH3OH4CO + 0.0291ISOP1CO2OH3OOH4N + 0.0291ISOP1OOH2OH3CO4N + 0.0291ISOP1OOH2OH3N4CO + 0.0291ISOP1N2OH3CO4OOH + 0.0291ISOP1OH2OOH3CO4N + 0.0291ISOP1N4CO4OOH + 0.0291ISOP1N4CO4OH + 0.0291ISOP1CO1OOH4N + 0.0291ISOP1CO1OH4N + 0.0291ISOP1N2OH3OOH4CO + 0.0291ISOP1CO2OOH3OH4N + 0.0291ISOP1CO2N3OH4OOH : 3.0E-12 ;
1.0OH + 1.0ISOP1OH2OOH3N4OH = 0.33OH + 0.67HO2 + 0.165ISOP1OH2N3CO4OH + 0.165ISOP1OH2OH3N4CO + 0.0291ISOP1CO2N3OOH4OH + 0.0291ISOP1CO2OOH3N4OH + 0.0291ISOP1OH2OOH3N4CO + 0.0291ISOP1OH2N3OOH4CO + 0.0291ISOP1CO2N3OOH4OOH + 0.0291ISOP1CO2OOH3N4OOH + 0.0291ISOP1CO2OOH3OOH4N + 0.0291ISOP1OOH2OOH3N4CO + 0.0291ISOP1OOH2N3OOH4CO + 0.0291ISOP1N2OOH3OOH4CO + 0.0291ISOP1N2OOH3OH4CO + 0.0291ISOP1CO2OH3OOH4N + 0.0291ISOP1OOH2OH3CO4N + 0.0291ISOP1OOH2OH3N4CO + 0.0291ISOP1N2OH3CO4OOH + 0.0291ISOP1OH2OOH3CO4N + 0.0291ISOP1N4CO4OOH + 0.0291ISOP1N4CO4OH + 0.0291ISOP1CO1OOH4N + 0.0291ISOP1CO1OH4N +

0.0291ISOP1N2OH3OOH4CO + 0.0291ISOP1CO2OOH3OH4N + 0.0291ISOP1CO2N3OH4OOH : 3.0E-12 ;
1.0OH + 1.0ISOP1OH2N3OOH4OH = 0.33OH + 0.67HO2 + 0.165ISOP1OH2N3CO4OH + 0.165ISOP1OH2OH3N4CO + 0.0291ISOP1CO2N3OOH4OH + 0.0291ISOP1CO2OOH3N4OH + 0.0291ISOP1OH2OOH3N4CO + 0.0291ISOP1OH2N3OOH4CO + 0.0291ISOP1CO2N3OOH4OOH + 0.0291ISOP1CO2OOH3N4OOH + 0.0291ISOP1CO2OOH3OOH4N + 0.0291ISOP1OOH2OOH3N4CO + 0.0291ISOP1OOH2N3OOH4CO + 0.0291ISOP1N2OOH3OOH4CO + 0.0291ISOP1N2OOH3OH4CO + 0.0291ISOP1CO2OH3OOH4N + 0.0291ISOP1OOH2OH3CO4N + 0.0291ISOP1OOH2OH3N4CO + 0.0291ISOP1N2OH3CO4OOH + 0.0291ISOP1OH2OOH3CO4N + 0.0291ISOP1N4CO4OOH + 0.0291ISOP1N4CO4OH + 0.0291ISOP1CO1OOH4N + 0.0291ISOP1CO1OH4N + 0.0291ISOP1N2OH3OOH4CO + 0.0291ISOP1CO2OOH3OH4N + 0.0291ISOP1CO2N3OH4OOH : 3.0E-12 ;
1.0OH + 1.0ISOP1N2OOH3OH4OH = 0.33OH + 0.67HO2 + 0.165ISOP1OH2N3CO4OH + 0.165ISOP1OH2OH3N4CO + 0.0291ISOP1CO2N3OOH4OH + 0.0291ISOP1CO2OOH3N4OH + 0.0291ISOP1OH2OOH3N4CO + 0.0291ISOP1OH2N3OOH4CO + 0.0291ISOP1CO2N3OOH4OOH + 0.0291ISOP1CO2OOH3N4OOH + 0.0291ISOP1CO2OOH3OOH4N + 0.0291ISOP1OOH2OOH3N4CO + 0.0291ISOP1OOH2N3OOH4CO + 0.0291ISOP1N2OOH3OOH4CO + 0.0291ISOP1N2OOH3OH4CO + 0.0291ISOP1CO2OH3OOH4N + 0.0291ISOP1OOH2OH3CO4N + 0.0291ISOP1OOH2OH3N4CO + 0.0291ISOP1N2OH3CO4OOH + 0.0291ISOP1OH2OOH3CO4N + 0.0291ISOP1N4CO4OOH + 0.0291ISOP1N4CO4OH + 0.0291ISOP1CO1OOH4N + 0.0291ISOP1CO1OH4N + 0.0291ISOP1N2OH3OOH4CO + 0.0291ISOP1CO2OOH3OH4N + 0.0291ISOP1CO2N3OH4OOH : 3.0E-12 ;
1.0OH + 1.0ISOP1OH2OH3OOH4N = 0.33OH + 0.67HO2 + 0.165ISOP1OH2N3CO4OH + 0.165ISOP1OH2OH3N4CO + 0.0291ISOP1CO2N3OOH4OH + 0.0291ISOP1CO2OOH3N4OH + 0.0291ISOP1OH2OOH3N4CO + 0.0291ISOP1OH2N3OOH4CO + 0.0291ISOP1CO2N3OOH4OOH + 0.0291ISOP1CO2OOH3N4OOH + 0.0291ISOP1CO2OOH3OOH4N + 0.0291ISOP1OOH2OOH3N4CO + 0.0291ISOP1OOH2N3OOH4CO + 0.0291ISOP1N2OOH3OOH4CO + 0.0291ISOP1N2OOH3OH4CO + 0.0291ISOP1CO2OH3OOH4N + 0.0291ISOP1OOH2OH3CO4N + 0.0291ISOP1OOH2OH3N4CO + 0.0291ISOP1N2OH3CO4OOH + 0.0291ISOP1OH2OOH3CO4N + 0.0291ISOP1N4CO4OOH + 0.0291ISOP1N4CO4OH + 0.0291ISOP1CO1OOH4N + 0.0291ISOP1CO1OH4N + 0.0291ISOP1N2OH3OOH4CO + 0.0291ISOP1CO2OOH3OH4N + 0.0291ISOP1CO2N3OH4OOH : 3.0E-12 ;
1.0OH + 1.0ISOP1N2OH3OH4OOH = 0.33OH + 0.67HO2 + 0.165ISOP1OH2N3CO4OH + 0.165ISOP1OH2OH3N4CO + 0.0291ISOP1CO2N3OOH4OH + 0.0291ISOP1CO2OOH3N4OH + 0.0291ISOP1OH2OOH3N4CO + 0.0291ISOP1OH2N3OOH4CO + 0.0291ISOP1CO2N3OOH4OOH + 0.0291ISOP1CO2OOH3N4OOH + 0.0291ISOP1CO2OOH3OOH4N + 0.0291ISOP1OOH2OOH3N4CO + 0.0291ISOP1OOH2N3OOH4CO + 0.0291ISOP1N2OOH3OOH4CO + 0.0291ISOP1N2OOH3OH4CO + 0.0291ISOP1CO2OH3OOH4N + 0.0291ISOP1OOH2OH3CO4N + 0.0291ISOP1OOH2OH3N4CO + 0.0291ISOP1N2OH3CO4OOH + 0.0291ISOP1OH2OOH3CO4N + 0.0291ISOP1N4CO4OOH + 0.0291ISOP1N4CO4OH + 0.0291ISOP1CO1OOH4N + 0.0291ISOP1CO1OH4N + 0.0291ISOP1N2OH3OOH4CO + 0.0291ISOP1CO2OOH3OH4N + 0.0291ISOP1CO2N3OH4OOH : 3.0E-12 ;
1.0OH + 1.0ISOP1OOH2OH3OH4N = 0.33OH + 0.67HO2 + 0.165ISOP1OH2N3CO4OH + 0.165ISOP1OH2OH3N4CO + 0.0291ISOP1CO2N3OOH4OH + 0.0291ISOP1CO2OOH3N4OH + 0.0291ISOP1OH2OOH3N4CO + 0.0291ISOP1OH2N3OOH4CO + 0.0291ISOP1CO2N3OOH4OOH + 0.0291ISOP1CO2OOH3N4OOH + 0.0291ISOP1CO2OOH3OOH4N + 0.0291ISOP1OOH2OOH3N4CO + 0.0291ISOP1OOH2N3OOH4CO + 0.0291ISOP1N2OOH3OOH4CO + 0.0291ISOP1N2OOH3OH4CO + 0.0291ISOP1CO2OH3OOH4N + 0.0291ISOP1OOH2OH3CO4N + 0.0291ISOP1OOH2OH3N4CO + 0.0291ISOP1N2OH3CO4OOH + 0.0291ISOP1OH2OOH3CO4N + 0.0291ISOP1N4CO4OOH +

0.0291ISOP1N4CO4OH + 0.0291ISOP1CO1OOH4N + 0.0291ISOP1CO1OH4N + 0.0291ISOP1N2OH3OOH4CO + 0.0291ISOP1CO2OOH3OH4N + 0.0291ISOP1CO2N3OH4OOH : 3.0E-12 ;
1.0ISOP1OH2OOH3OH4N = 1.0OH + 0.7HO2 + 0.55HCHO + 0.5MACR2N3OH + 0.3GLYC + 0.45HAC + 0.3NO2 + 0.15ETHLN + 0.025MVK3N4OH4OH + 0.025MVK3N4OH4OH : SUN(6.5E-6) ;*
1.0ISOP1OH2N3OH4OOH = 1.0OH + 0.7HO2 + 0.55HCHO + 0.5MACR2N3OH + 0.3GLYC + 0.45HAC + 0.3NO2 + 0.15ETHLN + 0.025MVK3N4OH4OH + 0.025MVK3N4OH4OH : SUN(6.5E-6) ;*
1.0ISOP1N2OH3OOH4OH = 1.0OH + 0.7HO2 + 0.55HCHO + 0.5MACR2N3OH + 0.3GLYC + 0.45HAC + 0.3NO2 + 0.15ETHLN + 0.025MVK3N4OH4OH + 0.025MVK3N4OH4OH : SUN(6.5E-6) ;*
1.0ISOP1OOH2OH3N4OH = 1.0OH + 0.7HO2 + 0.55HCHO + 0.5MACR2N3OH + 0.3GLYC + 0.45HAC + 0.3NO2 + 0.15ETHLN + 0.025MVK3N4OH4OH + 0.025MVK3N4OH4OH : SUN(6.5E-6) ;*
1.0ISOP1OH2OOH3N4OH = 1.0OH + 0.7HO2 + 0.55HCHO + 0.5MACR2N3OH + 0.3GLYC + 0.45HAC + 0.3NO2 + 0.15ETHLN + 0.025MVK3N4OH4OH + 0.025MVK3N4OH4OH : SUN(6.5E-6) ;*
1.0ISOP1OH2N3OOH4OH = 1.0OH + 0.7HO2 + 0.55HCHO + 0.5MACR2N3OH + 0.3GLYC + 0.45HAC + 0.3NO2 + 0.15ETHLN + 0.025MVK3N4OH4OH + 0.025MVK3N4OH4OH : SUN(6.5E-6) ;*
1.0ISOP1N2OOH3OH4OH = 1.0OH + 0.7HO2 + 0.55HCHO + 0.5MACR2N3OH + 0.3GLYC + 0.45HAC + 0.3NO2 + 0.15ETHLN + 0.025MVK3N4OH4OH + 0.025MVK3N4OH4OH : SUN(6.5E-6) ;*
1.0ISOP1OH2OH3OOH4N = 1.0OH + 0.7HO2 + 0.55HCHO + 0.5MACR2N3OH + 0.3GLYC + 0.45HAC + 0.3NO2 + 0.15ETHLN + 0.025MVK3N4OH4OH + 0.025MVK3N4OH4OH : SUN(6.5E-6) ;*
1.0ISOP1N2OH3OH4OOH = 1.0OH + 0.7HO2 + 0.55HCHO + 0.5MACR2N3OH + 0.3GLYC + 0.45HAC + 0.3NO2 + 0.15ETHLN + 0.025MVK3N4OH4OH + 0.025MVK3N4OH4OH : SUN(6.5E-6) ;*
1.0ISOP1OOH2OH3OH4N = 1.0OH + 0.7HO2 + 0.55HCHO + 0.5MACR2N3OH + 0.3GLYC + 0.45HAC + 0.3NO2 + 0.15ETHLN + 0.025MVK3N4OH4OH + 0.025MVK3N4OH4OH : SUN(6.5E-6) ;*
1.0ISOP1OH2OOH3OH4N = 1.0NO2 + 0.8HAC + 0.7HO2 + 0.5HPETHNL + 0.3OH + 0.35GLYC + 0.15HCHO + 0.15MACR2OOH3OH + 0.05HPAC : SUN(5E-6) ;*
1.0ISOP1OH2N3OH4OOH = 1.0NO2 + 0.8HAC + 0.7HO2 + 0.5HPETHNL + 0.3OH + 0.35GLYC + 0.15HCHO + 0.15MACR2OOH3OH + 0.05HPAC : SUN(5E-6) ;*
1.0ISOP1N2OH3OOH4OH = 1.0NO2 + 0.8HAC + 0.7HO2 + 0.5HPETHNL + 0.3OH + 0.35GLYC + 0.15HCHO + 0.15MACR2OOH3OH + 0.05HPAC : SUN(5E-6) ;*
1.0ISOP1OOH2OH3N4OH = 1.0NO2 + 0.8HAC + 0.7HO2 + 0.5HPETHNL + 0.3OH + 0.35GLYC + 0.15HCHO + 0.15MACR2OOH3OH + 0.05HPAC : SUN(5E-6) ;*
1.0ISOP1OH2OOH3N4OH = 1.0NO2 + 0.8HAC + 0.7HO2 + 0.5HPETHNL + 0.3OH + 0.35GLYC + 0.15HCHO + 0.15MACR2OOH3OH + 0.05HPAC : SUN(5E-6) ;*
1.0ISOP1OH2N3OOH4OH = 1.0NO2 + 0.8HAC + 0.7HO2 + 0.5HPETHNL + 0.3OH + 0.35GLYC + 0.15HCHO + 0.15MACR2OOH3OH + 0.05HPAC : SUN(5E-6) ;*
1.0ISOP1N2OOH3OH4OH = 1.0NO2 + 0.8HAC + 0.7HO2 + 0.5HPETHNL + 0.3OH + 0.35GLYC + 0.15HCHO + 0.15MACR2OOH3OH + 0.05HPAC : SUN(5E-6) ;*
1.0ISOP1OH2OH3OOH4N = 1.0NO2 + 0.8HAC + 0.7HO2 + 0.5HPETHNL + 0.3OH + 0.35GLYC + 0.15HCHO + 0.15MACR2OOH3OH + 0.05HPAC : SUN(5E-6) ;*
1.0ISOP1N2OH3OH4OOH = 1.0NO2 + 0.8HAC + 0.7HO2 + 0.5HPETHNL + 0.3OH + 0.35GLYC + 0.15HCHO + 0.15MACR2OOH3OH + 0.05HPAC : SUN(5E-6) ;*
1.0ISOP1OOH2OH3OH4N = 1.0NO2 + 0.8HAC + 0.7HO2 + 0.5HPETHNL + 0.3OH + 0.35GLYC + 0.15HCHO + 0.15MACR2OOH3OH + 0.05HPAC : SUN(5E-6) ;*
1.0OH + 1.0ISOP1OH2N3CO4OH = 1.0CO + 1.0NO2 + 0.75MVK3OH4OH + 0.25MACR2OH3OH : 1.0E-11 ;
1.0OH + 1.0ISOP1OH2OH3N4CO = 1.0CO + 1.0NO2 + 0.75MVK3OH4OH + 0.25MACR2OH3OH : 1.0E-11 ;
*1.0ISOP1OH2N3CO4OH = 0.5HO2 + 1.0NO2 + 0.5CO + 0.375MVK3OH4OH + 0.125MACR2OH3OH + 0.5HOCH2CO3 + 0.5HAC : SUN*3.0E-5 ;*

*1.0ISOP1OH2OH3N4CO = 0.5HO2 + 1.0NO2 + 0.5CO + 0.375MVK3OH4OH + 0.125MACR2OH3OH + 0.5HOCH2CO3 + 0.5HAC : SUN*3.0E-5 ;*
*1.0ISOP1OH2N3CO4OH = 1.0NO2 + 0.5HO2 + 0.375GLYC + 0.375MGLY + 0.125GLYX + 0.625HAC + 0.5HOCH2CO3 : SUN*5.0E-6 ;*
*1.0ISOP1OH2OH3N4CO = 1.0NO2 + 0.5HO2 + 0.375GLYC + 0.375MGLY + 0.125GLYX + 0.625HAC + 0.5HOCH2CO3 : SUN*5.0E-6 ;*
1.0OH + 1.0ISOP1OH2OOH3OOH4CO = 1.0CO + 0.5HO2 + 0.5OH + 0.5MACR2OOH3OH + 0.35MVK3OH4OH + 0.15MACR2OH3OH : 1.0E-11 ;
1.0OH + 1.0ISOP1CO2OOH3OOH4OH = 1.0CO + 0.5HO2 + 0.5OH + 0.5MACR2OOH3OH + 0.35MVK3OH4OH + 0.15MACR2OH3OH : 1.0E-11 ;
1.0OH + 1.0ISOP1OH2OOH3CO4OH = 1.0CO + 0.5HO2 + 0.5OH + 0.5MACR2OOH3OH + 0.35MVK3OH4OH + 0.15MACR2OH3OH : 1.0E-11 ;
1.0OH + 1.0ISOP1CO2OOH3OOH4OOH = 1.0CO + 0.5HO2 + 0.5OH + 0.5MACR2OOH3OH + 0.35MVK3OH4OH + 0.15MACR2OH3OH : 1.0E-11 ;
1.0OH + 1.0ISOP1OOH2OOH3OOH4CO = 1.0CO + 0.5HO2 + 0.5OH + 0.5MACR2OOH3OH + 0.35MVK3OH4OH + 0.15MACR2OH3OH : 1.0E-11 ;
1.0OH + 1.0ISOP1OH2OOH3OH4CO = 1.0CO + 0.5HO2 + 0.5OH + 0.5MACR2OOH3OH + 0.35MVK3OH4OH + 0.15MACR2OH3OH : 1.0E-11 ;
1.0OH + 1.0ISOP1OOH2OH3CO4OH = 1.0CO + 0.5HO2 + 0.5OH + 0.5MACR2OOH3OH + 0.35MVK3OH4OH + 0.15MACR2OH3OH : 1.0E-11 ;
1.0OH + 1.0ISOP1CO2OH3OOH4OH = 1.0CO + 0.5HO2 + 0.5OH + 0.5MACR2OOH3OH + 0.35MVK3OH4OH + 0.15MACR2OH3OH : 1.0E-11 ;
1.0OH + 1.0ISOP1OH3OH4CO = 1.0CO + 0.5HO2 + 0.5OH + 0.5MACR2OOH3OH + 0.35MVK3OH4OH + 0.15MACR2OH3OH : 1.0E-11 ;
1.0OH + 1.0ISOP1CO3OH4OH = 1.0CO + 0.5HO2 + 0.5OH + 0.5MACR2OOH3OH + 0.35MVK3OH4OH + 0.15MACR2OH3OH : 1.0E-11 ;
1.0OH + 1.0ISOP1OH2OH3OOH4CO = 1.0CO + 0.5HO2 + 0.5OH + 0.5MACR2OOH3OH + 0.35MVK3OH4OH + 0.15MACR2OH3OH : 1.0E-11 ;
1.0OH + 1.0ISOP1CO2OOH3OH4OH = 1.0CO + 0.5HO2 + 0.5OH + 0.5MACR2OOH3OH + 0.35MVK3OH4OH + 0.15MACR2OH3OH : 1.0E-11 ;
1.0OH + 1.0ISOP1CO1OH4OH = 1.0CO + 0.5HO2 + 0.5OH + 0.5MACR2OOH3OH + 0.35MVK3OH4OH + 0.15MACR2OH3OH : 1.0E-11 ;
1.0OH + 1.0ISOP1OH4CO4OH = 1.0CO + 0.5HO2 + 0.5OH + 0.5MACR2OOH3OH + 0.35MVK3OH4OH + 0.15MACR2OH3OH : 1.0E-11 ;
1.0OH + 1.0ISOP1CO1OOH4OH = 1.0CO + 0.5HO2 + 0.5OH + 0.5MACR2OOH3OH + 0.35MVK3OH4OH + 0.15MACR2OH3OH : 1.0E-11 ;
1.0OH + 1.0ISOP1OH4CO4OOH = 1.0CO + 0.5HO2 + 0.5OH + 0.5MACR2OOH3OH + 0.35MVK3OH4OH + 0.15MACR2OH3OH : 1.0E-11 ;
*1.0ISOP1OH2OOH3OOH4CO = 1.0CO + 1.5HO2 + 0.5OH + 0.5MACR2OOH3OH + 0.35MVK3OH4OH + 0.15MACR2OH3OH : SUN*3.0E-5 ;*
*1.0ISOP1CO2OOH3OOH4OH = 1.0CO + 1.5HO2 + 0.5OH + 0.5MACR2OOH3OH + 0.35MVK3OH4OH + 0.15MACR2OH3OH : SUN*3.0E-5 ;*
*1.0ISOP1OH2OOH3CO4OH = 1.0CO + 1.5HO2 + 0.5OH + 0.5MACR2OOH3OH + 0.35MVK3OH4OH + 0.15MACR2OH3OH : SUN*3.0E-5 ;*
*1.0ISOP1CO2OOH3OOH4OOH = 1.0CO + 1.5HO2 + 0.5OH + 0.5MACR2OOH3OH + 0.35MVK3OH4OH + 0.15MACR2OH3OH : SUN*3.0E-5 ;*
*1.0ISOP1OOH2OOH3OOH4CO = 1.0CO + 1.5HO2 + 0.5OH + 0.5MACR2OOH3OH + 0.35MVK3OH4OH + 0.15MACR2OH3OH : SUN*3.0E-5 ;*

*1.0ISOP1OH2OOH3OH4CO = 1.0CO + 1.5HO2 + 0.5OH + 0.5MACR2OOH3OH + 0.35MVK3OH4OH + 0.15MACR2OH3OH : SUN*3.0E-5 ;*
*1.0ISOP1OOH2OH3CO4OH = 1.0CO + 1.5HO2 + 0.5OH + 0.5MACR2OOH3OH + 0.35MVK3OH4OH + 0.15MACR2OH3OH : SUN*3.0E-5 ;*
*1.0ISOP1CO2OH3OOH4OH = 1.0CO + 1.5HO2 + 0.5OH + 0.5MACR2OOH3OH + 0.35MVK3OH4OH + 0.15MACR2OH3OH : SUN*3.0E-5 ;*
*1.0ISOP1OH3OH4CO = 1.0CO + 1.5HO2 + 0.5OH + 0.5MACR2OOH3OH + 0.35MVK3OH4OH + 0.15MACR2OH3OH : SUN*3.0E-5 ;*
*1.0ISOP1CO3OH4OH = 1.0CO + 1.5HO2 + 0.5OH + 0.5MACR2OOH3OH + 0.35MVK3OH4OH + 0.15MACR2OH3OH : SUN*3.0E-5 ;*
*1.0ISOP1OH2OH3OOH4CO = 1.0CO + 1.5HO2 + 0.5OH + 0.5MACR2OOH3OH + 0.35MVK3OH4OH + 0.15MACR2OH3OH : SUN*3.0E-5 ;*
*1.0ISOP1CO2OOH3OH4OH = 1.0CO + 1.5HO2 + 0.5OH + 0.5MACR2OOH3OH + 0.35MVK3OH4OH + 0.15MACR2OH3OH : SUN*3.0E-5 ;*
*1.0ISOP1CO1OH4OH = 1.0CO + 1.5HO2 + 0.5OH + 0.5MACR2OOH3OH + 0.35MVK3OH4OH + 0.15MACR2OH3OH : SUN*3.0E-5 ;*
*1.0ISOP1OH4CO4OH = 1.0CO + 1.5HO2 + 0.5OH + 0.5MACR2OOH3OH + 0.35MVK3OH4OH + 0.15MACR2OH3OH : SUN*3.0E-5 ;*
*1.0ISOP1CO1OOH4OH = 1.0CO + 1.5HO2 + 0.5OH + 0.5MACR2OOH3OH + 0.35MVK3OH4OH + 0.15MACR2OH3OH : SUN*3.0E-5 ;*
*1.0ISOP1OH4CO4OOH = 1.0CO + 1.5HO2 + 0.5OH + 0.5MACR2OOH3OH + 0.35MVK3OH4OH + 0.15MACR2OH3OH : SUN*3.0E-5 ;*
*1.0ISOP1OH2OOH3OOH4CO = 1.0OH + 1.0HO2 + 0.1HCHO + 0.1MVK3OOH4CO4OOH + 0.438HAC + 0.438GLYX + 0.088GLYC + 0.088MGLY + 0.122CO + 0.122MACR2OH3OH : SUN*6.5E-6 ;*
*1.0ISOP1CO2OOH3OOH4OH = 1.0OH + 1.0HO2 + 0.1HCHO + 0.1MVK3OOH4CO4OOH + 0.438HAC + 0.438GLYX + 0.088GLYC + 0.088MGLY + 0.122CO + 0.122MACR2OH3OH : SUN*6.5E-6 ;*
*1.0ISOP1OH2OOH3CO4OH = 1.0OH + 1.0HO2 + 0.1HCHO + 0.1MVK3OOH4CO4OOH + 0.438HAC + 0.438GLYX + 0.088GLYC + 0.088MGLY + 0.122CO + 0.122MACR2OH3OH : SUN*6.5E-6 ;*
*1.0ISOP1CO2OOH3OOH4OOH = 1.0OH + 1.0HO2 + 0.1HCHO + 0.1MVK3OOH4CO4OOH + 0.438HAC + 0.438GLYX + 0.088GLYC + 0.088MGLY + 0.122CO + 0.122MACR2OH3OH : SUN*6.5E-6 ;*
*1.0ISOP1OOH2OOH3OOH4CO = 1.0OH + 1.0HO2 + 0.1HCHO + 0.1MVK3OOH4CO4OOH + 0.438HAC + 0.438GLYX + 0.088GLYC + 0.088MGLY + 0.122CO + 0.122MACR2OH3OH : SUN*6.5E-6 ;*
*1.0ISOP1OH2OOH3OH4CO = 1.0OH + 1.0HO2 + 0.1HCHO + 0.1MVK3OOH4CO4OOH + 0.438HAC + 0.438GLYX + 0.088GLYC + 0.088MGLY + 0.122CO + 0.122MACR2OH3OH : SUN*6.5E-6 ;*
*1.0ISOP1OOH2OH3CO4OH = 1.0OH + 1.0HO2 + 0.1HCHO + 0.1MVK3OOH4CO4OOH + 0.438HAC + 0.438GLYX + 0.088GLYC + 0.088MGLY + 0.122CO + 0.122MACR2OH3OH : SUN*6.5E-6 ;*
*1.0ISOP1CO2OH3OOH4OH = 1.0OH + 1.0HO2 + 0.1HCHO + 0.1MVK3OOH4CO4OOH + 0.438HAC + 0.438GLYX + 0.088GLYC + 0.088MGLY + 0.122CO + 0.122MACR2OH3OH : SUN*6.5E-6 ;*
*1.0ISOP1OH3OH4CO = 1.0OH + 1.0HO2 + 0.1HCHO + 0.1MVK3OOH4CO4OOH + 0.438HAC + 0.438GLYX + 0.088GLYC + 0.088MGLY + 0.122CO + 0.122MACR2OH3OH : SUN*6.5E-6 ;*
*1.0ISOP1CO3OH4OH = 1.0OH + 1.0HO2 + 0.1HCHO + 0.1MVK3OOH4CO4OOH + 0.438HAC + 0.438GLYX + 0.088GLYC + 0.088MGLY + 0.122CO + 0.122MACR2OH3OH : SUN*6.5E-6 ;*
*1.0ISOP1OH2OH3OOH4CO = 1.0OH + 1.0HO2 + 0.1HCHO + 0.1MVK3OOH4CO4OOH + 0.438HAC + 0.438GLYX + 0.088GLYC + 0.088MGLY + 0.122CO + 0.122MACR2OH3OH : SUN*6.5E-6 ;*
*1.0ISOP1CO2OOH3OH4OH = 1.0OH + 1.0HO2 + 0.1HCHO + 0.1MVK3OOH4CO4OOH + 0.438HAC + 0.438GLYX + 0.088GLYC + 0.088MGLY + 0.122CO + 0.122MACR2OH3OH : SUN*6.5E-6 ;*
*1.0ISOP1CO1OH4OH = 1.0OH + 1.0HO2 + 0.1HCHO + 0.1MVK3OOH4CO4OOH + 0.438HAC + 0.438GLYX + 0.088GLYC + 0.088MGLY + 0.122CO + 0.122MACR2OH3OH : SUN*6.5E-6 ;*

1.0ISOP1OH4CO4OH = 1.0OH + 1.0HO2 + 0.1HCHO + 0.1MVK3OOH4CO4OOH + 0.438HAC + 0.438GLYX + 0.088GLYC + 0.088MGLY + 0.122CO + 0.122MACR2OH3OH : SUN*6.5E-6 ;
1.0ISOP1CO1OOH4OH = 1.0OH + 1.0HO2 + 0.1HCHO + 0.1MVK3OOH4CO4OOH + 0.438HAC + 0.438GLYX + 0.088GLYC + 0.088MGLY + 0.122CO + 0.122MACR2OH3OH : SUN*6.5E-6 ;
1.0ISOP1OH4CO4OOH = 1.0OH + 1.0HO2 + 0.1HCHO + 0.1MVK3OOH4CO4OOH + 0.438HAC + 0.438GLYX + 0.088GLYC + 0.088MGLY + 0.122CO + 0.122MACR2OH3OH : SUN*6.5E-6 ;
1.0OH + 1.0ISOP1OH2OOH3OH4OOH = 1.0OH + 0.0208ISOP1OH2OOH3OOH4CO + 0.0208ISOP1CO2OOH3OOH4OH + 0.0208ISOP1OH2OOH3CO4OH + 0.0208ISOP1CO2OOH3OOH4OOH + 0.0208ISOP1OOH2OOH3OOH4CO + 0.0208ISOP1OH2OOH3OH4CO + 0.0208ISOP1OOH2OH3CO4OH + 0.0208ISOP1CO2OH3OOH4OH + 0.0208ISOP1OH3OH4CO + 0.0208ISOP1CO3OH4OH + 0.0208ISOP1OH2OH3OOH4CO + 0.0208ISOP1CO2OOH3OH4OH + 0.0208ISOP1CO1OH4OH + 0.0208ISOP1OH4CO4OH + 0.0208ISOP1CO1OOH4OH + 0.0208ISOP1OH4CO4OOH + 0.111ISOP1CO2OOH34O4OH + 0.111ISOP1OH12O3OOH4CO + 0.111ISOP1OOH2OH34O4OH + 0.111ISOP1OH12O3OOH4OH + 0.111ISOP1OH12O3OH4OOH + 0.111ISOP1OH2OOH34O4OH : 3.0E-12 ;
1.0OH + 1.0ISOP1OOH2OH3OOH4OH = 1.0OH + 0.0208ISOP1OH2OOH3OOH4CO + 0.0208ISOP1CO2OOH3OOH4OH + 0.0208ISOP1OH2OOH3CO4OH + 0.0208ISOP1CO2OOH3OOH4OOH + 0.0208ISOP1OOH2OOH3OOH4CO + 0.0208ISOP1OH2OOH3OH4CO + 0.0208ISOP1OOH2OH3CO4OH + 0.0208ISOP1CO2OH3OOH4OH + 0.0208ISOP1OH3OH4CO + 0.0208ISOP1CO3OH4OH + 0.0208ISOP1OH2OH3OOH4CO + 0.0208ISOP1CO2OOH3OH4OH + 0.0208ISOP1CO1OH4OH + 0.0208ISOP1OH4CO4OH + 0.0208ISOP1CO1OOH4OH + 0.0208ISOP1OH4CO4OOH + 0.111ISOP1CO2OOH34O4OH + 0.111ISOP1OH12O3OOH4CO + 0.111ISOP1OOH2OH34O4OH + 0.111ISOP1OH12O3OOH4OH + 0.111ISOP1OH12O3OH4OOH + 0.111ISOP1OH2OOH34O4OH : 3.0E-12 ;
1.0OH + 1.0ISOP1OH2OOH3OOH4OH = 1.0OH + 0.0208ISOP1OH2OOH3OOH4CO + 0.0208ISOP1CO2OOH3OOH4OH + 0.0208ISOP1OH2OOH3CO4OH + 0.0208ISOP1CO2OOH3OOH4OOH + 0.0208ISOP1OOH2OOH3OOH4CO + 0.0208ISOP1OH2OOH3OH4CO + 0.0208ISOP1OOH2OH3CO4OH + 0.0208ISOP1CO2OH3OOH4OH + 0.0208ISOP1OH3OH4CO + 0.0208ISOP1CO3OH4OH + 0.0208ISOP1OH2OH3OOH4CO + 0.0208ISOP1CO2OOH3OH4OH + 0.0208ISOP1CO1OH4OH + 0.0208ISOP1OH4CO4OH + 0.0208ISOP1CO1OOH4OH + 0.0208ISOP1OH4CO4OOH + 0.111ISOP1CO2OOH34O4OH + 0.111ISOP1OH12O3OOH4CO + 0.111ISOP1OOH2OH34O4OH + 0.111ISOP1OH12O3OOH4OH + 0.111ISOP1OH12O3OH4OOH + 0.111ISOP1OH2OOH34O4OH : 3.0E-12 ;
1.0ISOP1OH2OOH3OH4OOH = 1.25OH + 0.25GLYC + 0.25HAC + 0.0469ISOP1OH2OOH3OOH4CO + 0.0469ISOP1CO2OOH3OOH4OH + 0.0469ISOP1OH2OOH3CO4OH + 0.0469ISOP1CO2OOH3OOH4OOH + 0.0469ISOP1OOH2OOH3OOH4CO + 0.0469ISOP1OH2OOH3OH4CO + 0.0469ISOP1OOH2OH3CO4OH + 0.0469ISOP1CO2OH3OOH4OH + 0.0469ISOP1OH3OH4CO + 0.0469ISOP1CO3OH4OH + 0.0469ISOP1OH2OH3OOH4CO + 0.0469ISOP1CO2OOH3OH4OH + 0.0469ISOP1CO1OH4OH + 0.0469ISOP1OH4CO4OH + 0.0469ISOP1CO1OOH4OH + 0.0469ISOP1OH4CO4OOH + 0.75HO2 : SUN*1.3E-5 ;
1.0ISOP1OOH2OH3OOH4OH = 1.25OH + 0.25GLYC + 0.25HAC + 0.0469ISOP1OH2OOH3OOH4CO + 0.0469ISOP1CO2OOH3OOH4OH + 0.0469ISOP1OH2OOH3CO4OH + 0.0469ISOP1CO2OOH3OOH4OOH + 0.0469ISOP1OOH2OOH3OOH4CO + 0.0469ISOP1OH2OOH3OH4CO + 0.0469ISOP1OOH2OH3CO4OH + 0.0469ISOP1CO2OH3OOH4OH + 0.0469ISOP1OH3OH4CO + 0.0469ISOP1CO3OH4OH + 0.0469ISOP1OH2OH3OOH4CO + 0.0469ISOP1CO2OOH3OH4OH + 0.0469ISOP1CO1OH4OH + 0.0469ISOP1OH4CO4OH + 0.0469ISOP1CO1OOH4OH + 0.0469ISOP1OH4CO4OOH + 0.75HO2 : SUN*1.3E-5 ;
1.0ISOP1OH2OOH3OOH4OH = 1.25OH + 0.25GLYC + 0.25HAC + 0.0469ISOP1OH2OOH3OOH4CO + 0.0469ISOP1CO2OOH3OOH4OH + 0.0469ISOP1OH2OOH3CO4OH +

*0.0469ISOP1CO2OOH3OOH4OOH + 0.0469ISOP1OOH2OOH3OOH4CO +
0.0469ISOP1OH2OOH3OH4CO + 0.0469ISOP1OOH2OH3CO4OH + 0.0469ISOP1CO2OH3OOH4OH +
0.0469ISOP1OH3OH4CO + 0.0469ISOP1CO3OH4OH + 0.0469ISOP1OH2OH3OOH4CO +
0.0469ISOP1CO2OOH3OH4OH + 0.0469ISOP1CO1OH4OH + 0.0469ISOP1OH4CO4OH +
0.0469ISOP1CO1OOH4OH + 0.0469ISOP1OH4CO4OOH + 0.75HO2 : SUN*1.3E-5 ;
1.0OH + 1.0ISOP1CO2OOH34O4OH = 1.0OH + 1.0CO2 + 0.571MACR2OOH3OH +
0.429MVK3OOH4OH4OH : 3.0E-12 ;
1.0OH + 1.0ISOP1OH12O3OOH4CO = 1.0OH + 1.0CO2 + 0.571MACR2OOH3OH +
0.429MVK3OOH4OH4OH : 3.0E-12 ;
1.0OH + 1.0ISOP1OOH2OH34O4OH = 1.0OH + 1.0CO2 + 0.571MACR2OOH3OH +
0.429MVK3OOH4OH4OH : 3.0E-12 ;
1.0OH + 1.0ISOP1OH12O3OOH4OH = 1.0OH + 1.0CO2 + 0.571MACR2OOH3OH +
0.429MVK3OOH4OH4OH : 3.0E-12 ;
1.0OH + 1.0ISOP1OH12O3OH4OOH = 1.0OH + 1.0CO2 + 0.571MACR2OOH3OH +
0.429MVK3OOH4OH4OH : 3.0E-12 ;
1.0OH + 1.0ISOP1OH2OOH34O4OH = 1.0OH + 1.0CO2 + 0.571MACR2OOH3OH +
0.429MVK3OOH4OH4OH : 3.0E-12 ;
1.0ISOP1CO2OOH34O4OH = 1.0OH + 1.0HO2 + 0.429MGLY + 0.429GLYC + 0.571GLYX + 0.571HAC :
SUN*6.49E-6 ;
1.0ISOP1OH12O3OOH4CO = 1.0OH + 1.0HO2 + 0.429MGLY + 0.429GLYC + 0.571GLYX + 0.571HAC :
SUN*6.49E-6 ;
1.0ISOP1OOH2OH34O4OH = 1.0OH + 1.0HO2 + 0.429MGLY + 0.429GLYC + 0.571GLYX + 0.571HAC :
SUN*6.49E-6 ;
1.0ISOP1OH12O3OOH4OH = 1.0OH + 1.0HO2 + 0.429MGLY + 0.429GLYC + 0.571GLYX + 0.571HAC :
SUN*6.49E-6 ;
1.0ISOP1OH12O3OH4OOH = 1.0OH + 1.0HO2 + 0.429MGLY + 0.429GLYC + 0.571GLYX + 0.571HAC :
SUN*6.49E-6 ;
1.0ISOP1OH2OOH34O4OH = 1.0OH + 1.0HO2 + 0.429MGLY + 0.429GLYC + 0.571GLYX + 0.571HAC :
SUN*6.49E-6 ;
1.0OH + 1.0ISOP1OOH23O3OH4N = 0.5HO2 + 1.0CO + 1.0OH + 0.5PROPNN + 0.5CO2 +
0.5MACR2OOH3N : 3.0E-12*EXP(20./TEMP);
1.0OH + 1.0ISOP1N2OOH34O4OH = 0.5HO2 + 1.0CO + 1.0OH + 0.5PROPNN + 0.5CO2 +
0.5MACR2OOH3N : 3.0E-12*EXP(20./TEMP);
1.0OH + 1.0ISOP1OH12O3OOH4N = 0.5HO2 + 1.0CO + 1.0OH + 0.5PROPNN + 0.5CO2 +
0.5MACR2OOH3N : 3.0E-12*EXP(20./TEMP);
1.0ISOP1OOH23O3OH4N = 1.0OH + 1.0PROPNN + 1.0HO2 + 1.0GLYX : SUN*6.49E-6;
1.0ISOP1N2OOH34O4OH = 1.0OH + 1.0PROPNN + 1.0HO2 + 1.0GLYX : SUN*6.49E-6;
1.0ISOP1OH12O3OOH4N = 1.0OH + 1.0PROPNN + 1.0HO2 + 1.0GLYX : SUN*6.49E-6;
1.0OH + 1.0ISOP1N2OOH3N4OH = 0.875IDNOO + 0.125OH + 0.0208ISOP1CO2N3OH4OH +
0.0208ISOP1N2OH3CO4N + 0.0208ISOP1OH2N3CO4N + 0.0208ISOP1N2N3CO4OH +
0.0208ISOP1N2OH3N4CO + 0.0208ISOP1CO2N3OH4N : 1.0E-12 ;
1.0OH + 1.0ISOP1N2OOH3OH4N = 0.875IDNOO + 0.125OH + 0.0208ISOP1CO2N3OH4OH +
0.0208ISOP1N2OH3CO4N + 0.0208ISOP1OH2N3CO4N + 0.0208ISOP1N2N3CO4OH +
0.0208ISOP1N2OH3N4CO + 0.0208ISOP1CO2N3OH4N : 1.0E-12 ;
1.0OH + 1.0ISOP1N2OH3N4OOH = 0.875IDNOO + 0.125OH + 0.0208ISOP1CO2N3OH4OH +
0.0208ISOP1N2OH3CO4N + 0.0208ISOP1OH2N3CO4N + 0.0208ISOP1N2N3CO4OH +
0.0208ISOP1N2OH3N4CO + 0.0208ISOP1CO2N3OH4N : 1.0E-12 ;*

1.0OH + 1.0ISOP1N2N3OH4OOH = 0.875IDNOO + 0.125OH + 0.0208ISOP1CO2N3OH4OH + 0.0208ISOP1N2OH3CO4N + 0.0208ISOP1OH2N3CO4N + 0.0208ISOP1N2N3CO4OH + 0.0208ISOP1N2OH3N4CO + 0.0208ISOP1CO2N3OH4N : 1.0E-12 ;
1.0OH + 1.0ISOP1OH2N3OOH4N = 0.875IDNOO + 0.125OH + 0.0208ISOP1CO2N3OH4OH + 0.0208ISOP1N2OH3CO4N + 0.0208ISOP1OH2N3CO4N + 0.0208ISOP1N2N3CO4OH + 0.0208ISOP1N2OH3N4CO + 0.0208ISOP1CO2N3OH4N : 1.0E-12 ;
1.0OH + 1.0ISOP1N2OH3OOH4N = 0.875IDNOO + 0.125OH + 0.0208ISOP1CO2N3OH4OH + 0.0208ISOP1N2OH3CO4N + 0.0208ISOP1OH2N3CO4N + 0.0208ISOP1N2N3CO4OH + 0.0208ISOP1N2OH3N4CO + 0.0208ISOP1CO2N3OH4N : 1.0E-12 ;
1.0OH + 1.0ISOP1OOH2N3OH4N = 0.875IDNOO + 0.125OH + 0.0208ISOP1CO2N3OH4OH + 0.0208ISOP1N2OH3CO4N + 0.0208ISOP1OH2N3CO4N + 0.0208ISOP1N2N3CO4OH + 0.0208ISOP1N2OH3N4CO + 0.0208ISOP1CO2N3OH4N : 1.0E-12 ;
1.0OH + 1.0ISOP1OOH2OH3N4N = 0.875IDNOO + 0.125OH + 0.0208ISOP1CO2N3OH4OH + 0.0208ISOP1N2OH3CO4N + 0.0208ISOP1OH2N3CO4N + 0.0208ISOP1N2N3CO4OH + 0.0208ISOP1N2OH3N4CO + 0.0208ISOP1CO2N3OH4N : 1.0E-12 ;
1.0OH + 1.0ISOP1N2N3OOH4OH = 0.875IDNOO + 0.125OH + 0.0208ISOP1CO2N3OH4OH + 0.0208ISOP1N2OH3CO4N + 0.0208ISOP1OH2N3CO4N + 0.0208ISOP1N2N3CO4OH + 0.0208ISOP1N2OH3N4CO + 0.0208ISOP1CO2N3OH4N : 1.0E-12 ;
1.0OH + 1.0ISOP1OH2OOH3N4N = 0.875IDNOO + 0.125OH + 0.0208ISOP1CO2N3OH4OH + 0.0208ISOP1N2OH3CO4N + 0.0208ISOP1OH2N3CO4N + 0.0208ISOP1N2N3CO4OH + 0.0208ISOP1N2OH3N4CO + 0.0208ISOP1CO2N3OH4N : 1.0E-12 ;
*1.0ISOP1N2OOH3N4OH = 1.0OH + 0.82PROPNN + 0.68HO2 + 0.32GLYC + 0.32NO2 + 0.03ISOP1CO2N3OH4OH + 0.03ISOP1N2OH3CO4N + 0.03ISOP1OH2N3CO4N + 0.03ISOP1N2N3CO4OH + 0.03ISOP1N2OH3N4CO + 0.03ISOP1CO2N3OH4N + 0.5ETHLN : SUN*6.5E-6 ;*
*1.0ISOP1N2OOH3OH4N = 1.0OH + 0.82PROPNN + 0.68HO2 + 0.32GLYC + 0.32NO2 + 0.03ISOP1CO2N3OH4OH + 0.03ISOP1N2OH3CO4N + 0.03ISOP1OH2N3CO4N + 0.03ISOP1N2N3CO4OH + 0.03ISOP1N2OH3N4CO + 0.03ISOP1CO2N3OH4N + 0.5ETHLN : SUN*6.5E-6 ;*
*1.0ISOP1N2OH3N4OOH = 1.0OH + 0.82PROPNN + 0.68HO2 + 0.32GLYC + 0.32NO2 + 0.03ISOP1CO2N3OH4OH + 0.03ISOP1N2OH3CO4N + 0.03ISOP1OH2N3CO4N + 0.03ISOP1N2N3CO4OH + 0.03ISOP1N2OH3N4CO + 0.03ISOP1CO2N3OH4N + 0.5ETHLN : SUN*6.5E-6 ;*
*1.0ISOP1N2N3OH4OOH = 1.0OH + 0.82PROPNN + 0.68HO2 + 0.32GLYC + 0.32NO2 + 0.03ISOP1CO2N3OH4OH + 0.03ISOP1N2OH3CO4N + 0.03ISOP1OH2N3CO4N + 0.03ISOP1N2N3CO4OH + 0.03ISOP1N2OH3N4CO + 0.03ISOP1CO2N3OH4N + 0.5ETHLN : SUN*6.5E-6 ;*
*1.0ISOP1OH2N3OOH4N = 1.0OH + 0.82PROPNN + 0.68HO2 + 0.32GLYC + 0.32NO2 + 0.03ISOP1CO2N3OH4OH + 0.03ISOP1N2OH3CO4N + 0.03ISOP1OH2N3CO4N + 0.03ISOP1N2N3CO4OH + 0.03ISOP1N2OH3N4CO + 0.03ISOP1CO2N3OH4N + 0.5ETHLN : SUN*6.5E-6 ;*
*1.0ISOP1N2OH3OOH4N = 1.0OH + 0.82PROPNN + 0.68HO2 + 0.32GLYC + 0.32NO2 + 0.03ISOP1CO2N3OH4OH + 0.03ISOP1N2OH3CO4N + 0.03ISOP1OH2N3CO4N + 0.03ISOP1N2N3CO4OH + 0.03ISOP1N2OH3N4CO + 0.03ISOP1CO2N3OH4N + 0.5ETHLN : SUN*6.5E-6 ;*
*1.0ISOP1OOH2N3OH4N = 1.0OH + 0.82PROPNN + 0.68HO2 + 0.32GLYC + 0.32NO2 + 0.03ISOP1CO2N3OH4OH + 0.03ISOP1N2OH3CO4N + 0.03ISOP1OH2N3CO4N + 0.03ISOP1N2N3CO4OH + 0.03ISOP1N2OH3N4CO + 0.03ISOP1CO2N3OH4N + 0.5ETHLN : SUN*6.5E-6 ;*

*1.0ISOP1OOH2OH3N4N = 1.0OH + 0.82PROPNN + 0.68HO2 + 0.32GLYC + 0.32NO2 + 0.03ISOP1CO2N3OH4OH + 0.03ISOP1N2OH3CO4N + 0.03ISOP1OH2N3CO4N + 0.03ISOP1N2N3CO4OH + 0.03ISOP1N2OH3N4CO + 0.03ISOP1CO2N3OH4N + 0.5ETHLN : SUN*6.5E-6 ;*
*1.0ISOP1N2N3OOH4OH = 1.0OH + 0.82PROPNN + 0.68HO2 + 0.32GLYC + 0.32NO2 + 0.03ISOP1CO2N3OH4OH + 0.03ISOP1N2OH3CO4N + 0.03ISOP1OH2N3CO4N + 0.03ISOP1N2N3CO4OH + 0.03ISOP1N2OH3N4CO + 0.03ISOP1CO2N3OH4N + 0.5ETHLN : SUN*6.5E-6 ;*
*1.0ISOP1OH2OOH3N4N = 1.0OH + 0.82PROPNN + 0.68HO2 + 0.32GLYC + 0.32NO2 + 0.03ISOP1CO2N3OH4OH + 0.03ISOP1N2OH3CO4N + 0.03ISOP1OH2N3CO4N + 0.03ISOP1N2N3CO4OH + 0.03ISOP1N2OH3N4CO + 0.03ISOP1CO2N3OH4N + 0.5ETHLN : SUN*6.5E-6 ;*
*1.0ISOP1N2OOH3N4OH = 1.0NO2 + 0.125ISOP1N2OH3OO4OH + 0.125ISOP1N2OO3OH4OH + 0.125IDHNBOO + 0.25PROPNN + 0.25MVK3OH4N + 0.0625MVK3N4OH4OH + 0.0625MVK3N4N + 0.375HCHO + 0.535OH + 0.09HO2 + 0.09HPETHNL + 0.16GLYC : SUN*5.0E-6*2.0 ;*
*1.0ISOP1N2OOH3OH4N = 1.0NO2 + 0.125ISOP1N2OH3OO4OH + 0.125ISOP1N2OO3OH4OH + 0.125IDHNBOO + 0.25PROPNN + 0.25MVK3OH4N + 0.0625MVK3N4OH4OH + 0.0625MVK3N4N + 0.375HCHO + 0.535OH + 0.09HO2 + 0.09HPETHNL + 0.16GLYC : SUN*5.0E-6*2.0 ;*
*1.0ISOP1N2OH3N4OOH = 1.0NO2 + 0.125ISOP1N2OH3OO4OH + 0.125ISOP1N2OO3OH4OH + 0.125IDHNBOO + 0.25PROPNN + 0.25MVK3OH4N + 0.0625MVK3N4OH4OH + 0.0625MVK3N4N + 0.375HCHO + 0.535OH + 0.09HO2 + 0.09HPETHNL + 0.16GLYC : SUN*5.0E-6*2.0 ;*
*1.0ISOP1N2N3OH4OOH = 1.0NO2 + 0.125ISOP1N2OH3OO4OH + 0.125ISOP1N2OO3OH4OH + 0.125IDHNBOO + 0.25PROPNN + 0.25MVK3OH4N + 0.0625MVK3N4OH4OH + 0.0625MVK3N4N + 0.375HCHO + 0.535OH + 0.09HO2 + 0.09HPETHNL + 0.16GLYC : SUN*5.0E-6*2.0 ;*
*1.0ISOP1OH2N3OOH4N = 1.0NO2 + 0.125ISOP1N2OH3OO4OH + 0.125ISOP1N2OO3OH4OH + 0.125IDHNBOO + 0.25PROPNN + 0.25MVK3OH4N + 0.0625MVK3N4OH4OH + 0.0625MVK3N4N + 0.375HCHO + 0.535OH + 0.09HO2 + 0.09HPETHNL + 0.16GLYC : SUN*5.0E-6*2.0 ;*
*1.0ISOP1N2OH3OOH4N = 1.0NO2 + 0.125ISOP1N2OH3OO4OH + 0.125ISOP1N2OO3OH4OH + 0.125IDHNBOO + 0.25PROPNN + 0.25MVK3OH4N + 0.0625MVK3N4OH4OH + 0.0625MVK3N4N + 0.375HCHO + 0.535OH + 0.09HO2 + 0.09HPETHNL + 0.16GLYC : SUN*5.0E-6*2.0 ;*
*1.0ISOP1OOH2N3OH4N = 1.0NO2 + 0.125ISOP1N2OH3OO4OH + 0.125ISOP1N2OO3OH4OH + 0.125IDHNBOO + 0.25PROPNN + 0.25MVK3OH4N + 0.0625MVK3N4OH4OH + 0.0625MVK3N4N + 0.375HCHO + 0.535OH + 0.09HO2 + 0.09HPETHNL + 0.16GLYC : SUN*5.0E-6*2.0 ;*
*1.0ISOP1OOH2OH3N4N = 1.0NO2 + 0.125ISOP1N2OH3OO4OH + 0.125ISOP1N2OO3OH4OH + 0.125IDHNBOO + 0.25PROPNN + 0.25MVK3OH4N + 0.0625MVK3N4OH4OH + 0.0625MVK3N4N + 0.375HCHO + 0.535OH + 0.09HO2 + 0.09HPETHNL + 0.16GLYC : SUN*5.0E-6*2.0 ;*
*1.0ISOP1N2N3OOH4OH = 1.0NO2 + 0.125ISOP1N2OH3OO4OH + 0.125ISOP1N2OO3OH4OH + 0.125IDHNBOO + 0.25PROPNN + 0.25MVK3OH4N + 0.0625MVK3N4OH4OH + 0.0625MVK3N4N + 0.375HCHO + 0.535OH + 0.09HO2 + 0.09HPETHNL + 0.16GLYC : SUN*5.0E-6*2.0 ;*
*1.0ISOP1OH2OOH3N4N = 1.0NO2 + 0.125ISOP1N2OH3OO4OH + 0.125ISOP1N2OO3OH4OH + 0.125IDHNBOO + 0.25PROPNN + 0.25MVK3OH4N + 0.0625MVK3N4OH4OH + 0.0625MVK3N4N + 0.375HCHO + 0.535OH + 0.09HO2 + 0.09HPETHNL + 0.16GLYC : SUN*5.0E-6*2.0 ;*
*1.0OH + 1.0ISOP1CO2N3OH4OH = 1.0CO + 1.0NO2 + 0.167MACR2OH3N + 0.167MACR2N3N + 0.167MACR4N + 0.5MVK3OH4N : 1.0E-11*0.4 ;*
*1.0OH + 1.0ISOP1N2OH3CO4N = 1.0CO + 1.0NO2 + 0.167MACR2OH3N + 0.167 MACR2N3N + 0.167MACR4N + 0.5MVK3OH4N : 1.0E-11*0.4 ;*
*1.0OH + 1.0ISOP1OH2N3CO4N = 1.0CO + 1.0NO2 + 0.167MACR2OH3N + 0.167MACR2N3N + 0.167MACR4N + 0.5MVK3OH4N : 1.0E-11*0.4 ;*

1.0OH + 1.0ISOP1N2N3CO4OH = 1.0CO + 1.0NO2 + 0.167MACR2OH3N + 0.167MACR2N3N +
0.167MACR4N + 0.5MVK3OH4N : 1.0E-11*0.4 ;
1.0OH + 1.0ISOP1N2OH3N4CO = 1.0CO + 1.0NO2 + 0.167MACR2OH3N + 0.167MACR2N3N +
0.167MACR4N + 0.5MVK3OH4N : 1.0E-11*0.4 ;
1.0OH + 1.0ISOP1CO2N3OH4N = 1.0CO + 1.0NO2 + 0.167MACR2OH3N + 0.167MACR2N3N +
0.167MACR4N + 0.5MVK3OH4N : 1.0E-11*0.4 ;
1.0ISOP1CO2N3OH4OH = 1.5NO2 + 0.65CO + 0.1MACR2OH3OH + 0.1MVK3OH4OH +
0.075MVK3OH4N + 0.025MACR2OH3N + 0.025MACR4N + 0.025MACR2OH3N + 0.275HO2 +
0.025GLYX + 0.025PROPNN + 0.025MGLY + 0.025ETHLN + 0.525HAC + 0.375HCHO +
0.075MVK3CO4N + 0.225NCH2CO3 : SUN*5.0E-6*2.0 ;
1.0ISOP1N2OH3CO4N = 1.5NO2 + 0.65CO + 0.1MACR2OH3OH + 0.1MVK3OH4OH +
0.075MVK3OH4N + 0.025MACR2OH3N + 0.025MACR4N + 0.025MACR2N3N + 0.275HO2 +
0.025GLYX + 0.025PROPNN + 0.025MGLY + 0.025ETHLN + 0.525HAC + 0.375HCHO +
0.075MVK3CO4N + 0.225NCH2CO3 : SUN*5.0E-6*2.0 ;
1.0ISOP1OH2N3CO4N = 1.5NO2 + 0.65CO + 0.1MACR2OH3OH + 0.1MVK3OH4OH +
0.075MVK3OH4N + 0.025MACR2OH3N + 0.025MACR4N + 0.025MACR2N3N + 0.275HO2 +
0.025GLYX + 0.025PROPNN + 0.025MGLY + 0.025ETHLN + 0.525HAC + 0.375HCHO +
0.075MVK3CO4N + 0.225NCH2CO3 : SUN*5.0E-6*2.0 ;
1.0ISOP1N2N3CO4OH = 1.5NO2 + 0.65CO + 0.1MACR2OH3OH + 0.1MVK3OH4OH +
0.075MVK3OH4N + 0.025MACR2OH3N + 0.025MACR4N + 0.025MACR2N3N + 0.275HO2 +
0.025GLYX + 0.025PROPNN + 0.025MGLY + 0.025ETHLN + 0.525HAC + 0.375HCHO +
0.075MVK3CO4N + 0.225NCH2CO3 : SUN*5.0E-6*2.0 ;
1.0ISOP1N2OH3N4CO = 1.5NO2 + 0.65CO + 0.1MACR2OH3OH + 0.1MVK3OH4OH +
0.075MVK3OH4N + 0.025MACR2OH3N + 0.025MACR4N + 0.025MACR2N3N + 0.275HO2 +
0.025GLYX + 0.025PROPNN + 0.025MGLY + 0.025ETHLN + 0.525HAC + 0.375HCHO +
0.075MVK3CO4N + 0.225NCH2CO3 : SUN*5.0E-6*2.0 ;
1.0ISOP1CO2N3OH4N = 1.5NO2 + 0.65CO + 0.1MACR2OH3OH + 0.1MVK3OH4OH +
0.075MVK3OH4N + 0.025MACR2OH3N + 0.025MACR4N + 0.025MACR2N3N + 0.275HO2 +
0.025GLYX + 0.025PROPNN + 0.025MGLY + 0.025ETHLN + 0.525HAC + 0.375HCHO +
0.075MVK3CO4N + 0.225NCH2CO3 : SUN*5.0E-6*2.0 ;
1.0ISOP1CO2N3OH4OH = 1.0NO2 + 0.6HAC + 0.6NCH2CO3 + 0.4HO2 + 0.4CO +
0.0667MACR2OH3N + 0.0667MACR2N3N + 0.0667 MACR4N + 0.2MVK3OH4N : SUN*3.0E-5 ;
1.0ISOP1N2OH3CO4N = 1.0NO2 + 0.6HAC + 0.6NCH2CO3 + 0.4HO2 + 0.4CO + 0.0667MACR2OH3N
+ 0.0667MACR2N3N + 0.0667MACR4N + 0.2MVK3OH4N : SUN*3.0E-5 ;
1.0ISOP1OH2N3CO4N = 1.0NO2 + 0.6HAC + 0.6NCH2CO3 + 0.4HO2 + 0.4CO + 0.0667MACR2OH3N
+ 0.0667MACR2N3N + 0.0667MACR4N + 0.2MVK3OH4N : SUN*3.0E-5 ;
1.0ISOP1N2N3CO4OH = 1.0NO2 + 0.6HAC + 0.6NCH2CO3 + 0.4HO2 + 0.4CO + 0.0667MACR2OH3N
+ 0.0667MACR2N3N + 0.0667MACR4N + 0.2MVK3OH4N : SUN*3.0E-5 ;
1.0ISOP1N2OH3N4CO = 1.0NO2 + 0.6HAC + 0.6NCH2CO3 + 0.4HO2 + 0.4CO + 0.0667MACR2OH3N
+ 0.0667MACR2N3N + 0.0667MACR4N + 0.2MVK3OH4N : SUN*3.0E-5 ;
1.0ISOP1CO2N3OH4N = 1.0NO2 + 0.6HAC + 0.6NCH2CO3 + 0.4HO2 + 0.4CO + 0.0667MACR2OH3N
+ 0.0667MACR2N3N + 0.0667MACR4N + 0.2MVK3OH4N : SUN*3.0E-5 ;
1.0OH + 1.0ISOP1CO2OOH3OOH4CO = 0.888CO + 0.444OH + 0.444HO2 +
0.318MVK3OOH4CO4OOH + 0.08ISOP1CO2OO3OH4OH + 0.126MACR2OOH3CO3OOH +
0.444MVK3OOH4CO + 0.032ISOP1OH2OH3OO4CO : 2.25E-11 ;
1.0OH + 1.0ISOP1OOH2OOH3CO4CO = 0.888CO + 0.444OH + 0.444HO2 +
0.318MVK3OOH4CO4OOH + 0.08ISOP1CO2OO3OH4OH + 0.126MACR2OOH3CO3OOH +
0.444MVK3OOH4CO + 0.032ISOP1OH2OH3OO4CO : 2.25E-11 ;

1.0OH + 1.0ISOP1CO2OOH3OH4CO = 0.888CO + 0.444OH + 0.444HO2 + 0.318MVK3OOH4CO4OOH + 0.08ISOP1CO2OO3OH4OH + 0.126MACR2OOH3CO3OOH + 0.444MVK3OOH4CO + 0.032ISOP1OH2OH3OO4CO : 2.25E-11 ;
1.0OH + 1.0ISOP1OH2OOH3CO4CO = 0.888CO + 0.444OH + 0.444HO2 + 0.318MVK3OOH4CO4OOH + 0.08ISOP1CO2OO3OH4OH + 0.126MACR2OOH3CO3OOH + 0.444MVK3OOH4CO + 0.032ISOP1OH2OH3OO4CO : 2.25E-11 ;
1.0OH + 1.0ISOP1CO2OH3OOH4CO = 0.888CO + 0.444OH + 0.444HO2 + 0.318MVK3OOH4CO4OOH + 0.08ISOP1CO2OO3OH4OH + 0.126MACR2OOH3CO3OOH + 0.444MVK3OOH4CO + 0.032ISOP1OH2OH3OO4CO : 2.25E-11 ;
1.0OH + 1.0ISOP1OOH2OH3CO4CO = 0.888CO + 0.444OH + 0.444HO2 + 0.318MVK3OOH4CO4OOH + 0.08ISOP1CO2OO3OH4OH + 0.126MACR2OOH3CO3OOH + 0.444MVK3OOH4CO + 0.032ISOP1OH2OH3OO4CO : 2.25E-11 ;
*1.0ISOP1CO2OOH3OOH4CO = 0.546OH + 1.0CO + 1.45HO2 + 0.391MVK3OOH4CO4OOH + 0.155MACR2OOH3CO3OOH + 0.454MVK3OOH4CO : SUN*6.65E-5 ;*
*1.0ISOP1OOH2OOH3CO4CO = 0.546OH + 1.0CO + 1.45HO2 + 0.391MVK3OOH4CO4OOH + 0.155MACR2OOH3CO3OOH + 0.454MVK3OOH4CO : SUN*6.65E-5 ;*
*1.0ISOP1CO2OOH3OH4CO = 0.546OH + 1.0CO + 1.45HO2 + 0.391MVK3OOH4CO4OOH + 0.155MACR2OOH3CO3OOH + 0.454MVK3OOH4CO : SUN*6.65E-5 ;*
*1.0ISOP1OH2OOH3CO4CO = 0.546OH + 1.0CO + 1.45HO2 + 0.391MVK3OOH4CO4OOH + 0.155MACR2OOH3CO3OOH + 0.454MVK3OOH4CO : SUN*6.65E-5 ;*
*1.0ISOP1CO2OH3OOH4CO = 0.546OH + 1.0CO + 1.45HO2 + 0.391MVK3OOH4CO4OOH + 0.155MACR2OOH3CO3OOH + 0.454MVK3OOH4CO : SUN*6.65E-5 ;*
*1.0ISOP1OOH2OH3CO4CO = 0.546OH + 1.0CO + 1.45HO2 + 0.391MVK3OOH4CO4OOH + 0.155MACR2OOH3CO3OOH + 0.454MVK3OOH4CO : SUN*6.65E-5 ;*
CH3COOCH2 + OH = HOCH2COCHO + HO2 : 5.25e-12 ;
CH3COOCH2 = HOCH2CO3 + HCHO + HO2 : J(22) ;
*NPA + HO2 = HCHO + NO2 + OH : KAPHO2(TEMP)*0.44 ;*
*NPA + HO2 = NO3CH2CO2H + O3 : KAPHO2(TEMP)*0.15 ;*
*NPA + HO2 = NPAH : KAPHO2(TEMP)*0.41 ;*
NPA + NO = HCHO + NO2 + NO2 : KAPNO(TEMP) ;
NPA + NO2 = NO3CH2PAN : KFPAN(TEMP,M) ;
*NPA + NO3 = HCHO + NO2 + NO2 : KRO2NO3()*1.74 ;*
*NPA = HCHO + NO2 : 1.00e-11*0.7*0.1 ;*
*NPA = NO3CH2CO2H : 1.00e-11*0.3*0.1 ;*
HOCH2COCHO = HOCH2CO3 + CO + HO2 : J(34) ;
*NO3 + HOCH2COCHO = HOCH2CO3 + CO + HNO3 : KNO3AL(TEMP)*2.4 ;*
OH + HOCH2COCHO = HOCH2CO3 + CO : 1.44e-11 ;
*HOCH2CO3 + HO2 = HO2 + HCHO + OH : KAPHO2(TEMP)*0.44 ;*
*HOCH2CO3 + HO2 = HOCH2CO2H + O3 : KAPHO2(TEMP)*0.15 ;*
*HOCH2CO3 + HO2 = HOCH2CO3H : KAPHO2(TEMP)*0.41 ;*
HOCH2CO3 + NO = NO2 + HO2 + HCHO : KAPNO(TEMP) ;
HOCH2CO3 + NO2 = PHAN : KFPAN(TEMP,M) ;
*HOCH2CO3 + NO3 = NO2 + HO2 + HCHO : KRO2NO3()*1.74 ;*
*HOCH2CO3 = HCHO + HO2 : 1.00e-11*0.7*0.1 ;*
*HOCH2CO3 = HOCH2CO2H : 1.00e-11*0.3*0.1 ;*
NO3CH2CO2H + OH = HCHO + NO2 : 1.68e-13 ;
NPAH + OH = NPA : 3.63e-12 ;
NPAH = HCHO + NO2 + OH : J(41) ;
NO3CH2PAN + OH = HCHO + CO + NO2 + NO2 : 1.12e-14 ;

NO3CH2PAN = NPA + NO2 : KBPAN(TEMP,M) ;
HOCH2CO2H + OH = HCHO + HO2 : 2.73e-12 ;
HOCH2CO3H + OH = HOCH2CO3 : 6.19e-12 ;
HOCH2CO3H = HCHO + HO2 + OH : J(41) ;
*HAC + OH = MGLYOX + HO2 : 1.6E-12*EXP(305/TEMP) ;*
HAC = CH3CO3 + HCHO + HO2 : J(22) ;

References:

1. Bates, K. H. and Jacob, D. J.: A new model mechanism for atmospheric oxidation of isoprene: global effects on oxidants, nitrogen oxides, organic products, and secondary organic aerosol, Atmospheric Chemistry and Physics, 19, 9613–9640, https://doi.org/10.5194/acp-19-9613-2019, 2019.
2. Muñoz, A.: Isoprene+NO+hydrogen peroxide + OH - Gas-phase oxidation - product study, Tech. rep., CEAM, url https://data.eurochamp.org/data-access/chamber-experiments/25320384-8599-4bc9-a35d-993a77cec7c, 2021a.
3. Muñoz, A.: Isoprene+ozone+carbon monoxide + O3 - Gas-phase oxidation - product study, Tech. rep., CEAM, url https://data.eurochamp.org/data-access/chamber-experiments/25320384-8599-4bc9-a35d-993a77cec7c, 2021b.
4. Muñoz, A. and Gómez-Alvarez, E.: Overview Of The Eurochamp Database Of European Atmosphere Simulation Chambers, in: Simulation and Assessment of Chemical Processes in a Multiphase Environment, pp. 61–70, Springer, 2008.
5. Nguyen, T. B., Crounse, J. D., Schwantes, R. H., Teng, A. P., Bates, K. H., Zhang, X., St. Clair, J. M., Brune, W. H., Tyndall, G. S., Keutsch, F. N., Seinfeld, J. H., and Wennberg, P. O.: Overview of the Focused Isoprene eXperiment at the California Institute of Technology (FIXCIT): mechanistic chamber studies on the oxidation of biogenic compounds, Atmospheric Chemistry and Physics, 14, 13 531–13 549, https://doi.org/10.5194/acp-14-13531-2014, 2014.
6. Vasquez, K. T., Crounse, J. D., Schulze, B. C., Bates, K. H., Teng, A. P., Xu, L., Allen, H. M., and Wennberg, P. O.: Rapid hydrolysis of tertiary isoprene nitrate efficiently removes NO_x from the atmosphere, Proceedings of the National Academy of Sciences, 117, 33 011–33 016, https://doi.org/10.1073/pnas.2017442117, 2020.
7. Wennberg, P. O., Bates, K. H., Crounse, J. D., Dodson, L. G., McVay, R. C., Mertens, L. A., Nguyen, T. B., Praske, E., Schwantes, R. H., Smarte, M. D., St Clair, J. M., Teng, A. P., Zhang, X., and Seinfeld, J. H.: Gas-Phase Reactions of Isoprene and Its Major Oxidation Products, Chemical Reviews, 118, 3337–3390, https://doi.org/10.1021/acs.chemrev.7b00439, pMID: 29522327, 2018.
